# Nanopore detection of single-nucleotide RNA mutations and modifications with programmable nanolatches

**DOI:** 10.1038/s41565-025-01965-6

**Published:** 2025-06-27

**Authors:** Yunxuan Li, Siong Chen Meng, Yesheng Wang, Casey M. Platnich, Max K. Earle, Elli Mylona, Plamena Naydenova, Stephen Baker, Jinbo Zhu, Ulrich F. Keyser

**Affiliations:** 1 Cavendish Laboratory, University of Cambridge, Cambridge, UK; 2 School of Biomedical Engineering, Faculty of Medicine, Dalian University of Technology, Dalian, China; 3 Cambridge Institute of Therapeutic Immunology and Infectious Disease, Jeffery Cheah Biomedical Centre, Cambridge Biomedical Campus, University of Cambridge, Cambridge, UK; 4 A*STAR Infectious Diseases Labs, Agency for Science, Technology and Research, Singapore, Singapore

**Keywords:** Biosensors, RNA nanotechnology, Nanopores

## Abstract

RNA mutations and modifications have been implicated in a wide range of pathophysiologies. However, current RNA detection methods are hindered by data complexity and error-prone protocols, restricting their widespread use. Here we present a solid-state nanopore-based approach, RNA single-nucleotide characterization and analysis nanolatch (RNA-SCAN) system, which simplifies the detection of nucleotide mutations and modifications in RNA with high resolution. Using phage RNA as a template, we tested multiple sequences and chemical modifications on nanolatches, allowing the detection of mismatches caused by nucleotide mutations through significant changes in positive event ratios using single-molecule nanopore measurements. This approach is also sensitive to modifications that either strengthen or weaken the interaction between the target RNA sequence and the nanolatch. As a proof-of-concept, we demonstrate successful discrimination of *Escherichia coli* and *Salmonella* spp. from total RNA based on nucleotide variations in their 16S rRNA, as well as quantification of different *Salmonella* spp. and detection of m^5^C1407 modification on *E. coli* 16S rRNA. The RNA-SCAN approach demonstrates the feasibility of combining RNA/DNA hybrid nanotechnology with nanopore sensing and diagnosing RNA-related health conditions.

## Abstract (web summary)

The hybridization thermodynamics of programmable DNA nanostructures forming metastable loop structures on RNA generate distinct electrical signals during translocation through solid-state nanopores, enabling the identification of single-base alterations.

## Main

Nucleic acids, including both DNA and RNA, frequently undergo mutations and modifications that can lead to human diseases^1–4^. RNA, specifically, not only transmits genetic information but also regulates gene expression, making it highly susceptible to dysfunction when altered^3,4^. Such RNA changes also occur in bacteria and viruses, contributing to antimicrobial resistance and virulence and complicating therapeutic development^5,6^. Therefore, it is essential to develop analytical methods to reliably identify RNA single-nucleotide mutations and modifications—the most common form of genetic variation^7,8^.

However, detecting these subtle structural variations remains challenging due to their minimal impact on the physicochemical properties of RNA. Conventional methods such as single-strand conformation polymorphism, denaturing high-performance liquid chromatography, liquid chromatography–mass spectrometry and protein-assisted assays are constrained by limited sensitivity, indirect readouts, complex workflows and the requirement for prior amplification or labelling^9,10^. More recently, next-generation sequencing^11,12^ has emerged as the gold standard for mapping single-nucleotide polymorphisms, with biological nanopore-based direct RNA sequencing^13,14^ demonstrating impressive capabilities. Nevertheless, broader adoption of nanopore sequencing faces several challenges, including high error rates, the need for large input quantities, RNA degradation and reliance on sophisticated computational pipelines^13,14^. These considerations become particularly substantial when redundant full-length sequence information is not necessary, sample material is scarce, native quantification is desired or time and cost efficiency are critical.

In response to these challenges, solid-state glass nanopore-based single-molecule sensing has emerged as a promising complementary approach^15–18^. During measurement, analyte translocation through the nanopore temporarily blocks the flow of ions, generating characteristic current responses that reveal the analyte’s properties (Supplementary Note 1). This technology not only offers operational simplicity and efficient data analysis but also enables reliable performance from minimal sample input through single-molecule detection. Importantly, it achieves versatility without requiring enzymatic interventions or amplification processes, thereby maintaining the intrinsic characteristics of native RNA samples. However, the relatively large pore size compared with individual nucleotides often causes small signals from single-base variations to be masked by thermal noise from the RNA strand, necessitating signal enhancement strategies to achieve sensitive and specific identification.

**Fig. 1:**
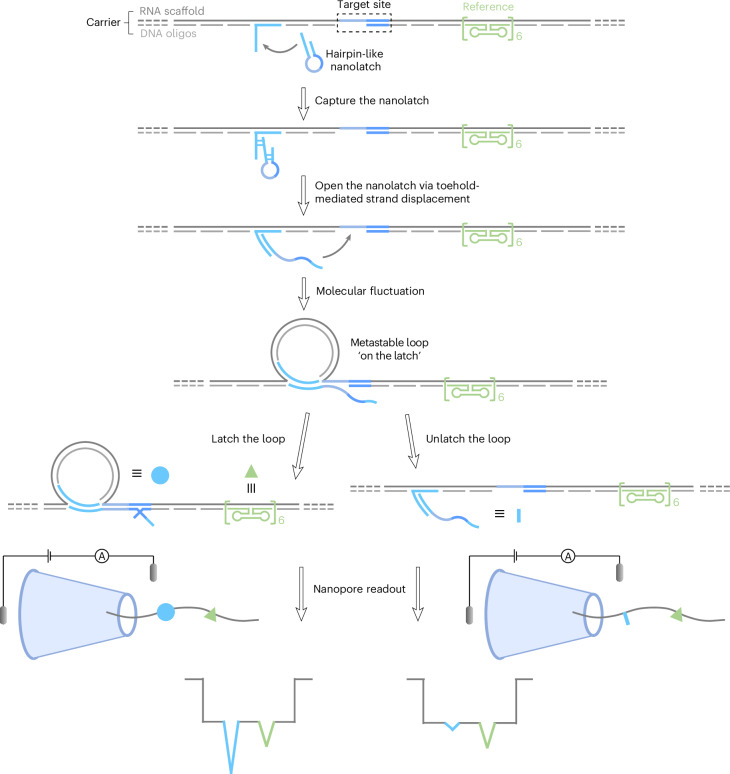
RNA-SCAN: a nanolatch-nanopore sensor for nucleic acid characterization. The RNA-SCAN mechanism proceeds as follows (where the complementary strand regions indicated by matching colours). A hairpin-like nanolatch is first captured and opened by an overhang on the RNA/DNA carrier (self-assembled from a long RNA scaffold and short DNA oligos) through toehold-mediated strand displacement, exposing its target site-complementary segment. Molecular fluctuations in solution then bring the nanolatch’s free end into proximity to the target site, forming a metastable loop structure referred to as the ‘on the latch’ state, a configuration that is formed but not yet fastened. The subsequent stability of the loop depends on base-pairing strength between the nanolatch and target site, resulting in either a ‘latched’ state (where strong complementarity fastens the loop) or an ‘unlatched’ state (where weak binding leads to loop opening). During nanopore detection, the translocation of the carrier generates a characteristic baseline current drop. A six-dumbbell structure (marked in green) positioned next to the target site consistently produces a small spike of approximately half the amplitude of the first-level current drop generated by the carrier alone, serving as a reference. In comparison, a latched loop typically generates a spike larger than the reference spike, approaching the magnitude of the first-level current drop, while an unlatched loop yields minimal current fluctuation beyond the plateau.

Here, we introduce RNA single-nucleotide characterization and analysis nanolatch, RNA-SCAN (Fig. 1), a strategy combining RNA/DNA origami nanotechnology with glass nanopore sensing for single-nucleotide resolution in complex RNA structures. In this approach, the target RNA molecule (‘scaffold’) is hybridized with designed DNA oligonucleotides (oligos) to form a stable RNA/DNA duplex, known as the ‘carrier’. This duplex protects the RNA from degradation^19,20^ and helps unfold its native structure, making the sequence more accessible. The programmable nature of DNA oligos also enables precise control over the carrier architecture and facilitates spatially-resolved sensing.

**Extended Data Fig. 1:**
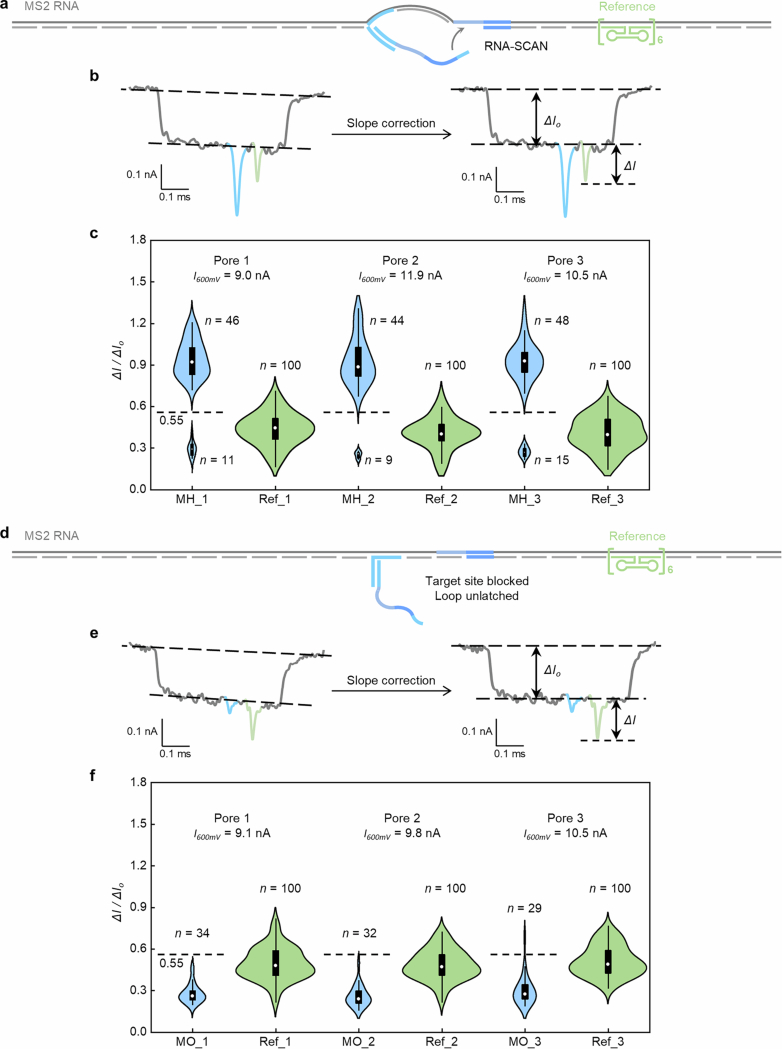
Comparison of nanopore current signals produced by latched loop and unlatched overhang structures in RNA-SCAN and target-blocked MS2 carrier designs. **a**, Schematic of the RNA-SCAN MS2 carrier design, featuring a reference structure and an open target site capable of forming a loop with the nanolatch. **b**, Representative nanopore translocation events of the RNA-SCAN carrier. A minor slope correction is applied to the baseline, which is likely due to slight changes in buffer concentration during the measurements. $\Delta I$ denotes the secondary current drop caused by structures on the carrier, while $\Delta {I}_{0}$ denotes the first-level current drop from the carrier backbone. $\Delta I/\Delta {I}_{0}$ is calculated to analyze the significance of nanopore signals generated by additional structures on the carrier. **c**, Analysis of $\Delta I/\Delta {I}_{0}$ values for the loop and reference structures on the RNA-SCAN carrier from three independent nanopore measurements. Box plots show the median (center line), the 25th and 75th percentiles (box bounds), and whiskers extending to 1.5× IQR; minima and maxima refer to the lowest and highest data points within this range. About 46 events in each measurement exhibit a distinct loop spike, with $\Delta I/\Delta {I}_{0}$ values mostly exceeding the 0.55 threshold used to distinguish positive events from negative events. $\Delta I/\Delta {I}_{0}$ values of the reference structure are consistently around 0.5. **d**, Schematic of the target-blocked MS2 carrier design, featuring a reference structure and a DNA overhang at the central. **e**, Representative nanopore translocation events of the target-blocked carrier, showing only small spikes or no spike at the target site. **f**, Analysis of $\Delta I/\Delta {I}_{0}$ values for the overhang and loop structures on the target-blocked carrier from three independent nanopore measurements. Only about 30 events exhibit an identifiable spike at the target site, with $\Delta I/\Delta {I}_{0}$ values generally below 0.55, though occasional outliers are observed. Source data

**Extended Data Fig. 2:**
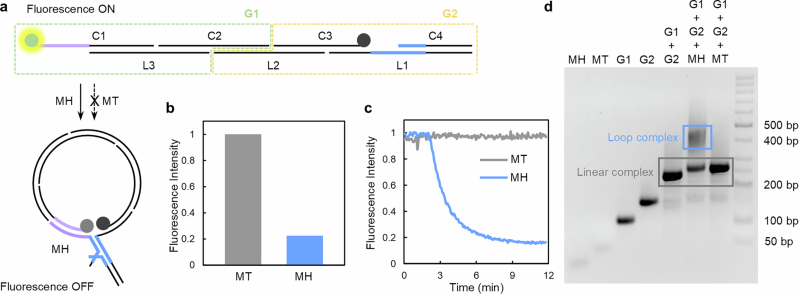
Verification of the nanolatch design using bulk methods. **a**, Schematic of the nanolatch design incorporating fluorophore and quencher pairs, independent of the MS2 carrier context. The positive control nanolatch (MH) induces loop formation by connecting the two ends of the linear complex, bringing the fluorophore and quencher into close proximity and resulting in a non-fluorescent ‘Fluorescence OFF’ state. In contrast, the negative control nanolatch (MT) fails to consistently latch the loop, allowing fluorescence emission and maintaining a ‘Fluorescence ON’ state. **b**,**c**, Fluorescence measurements comparing the MT and MH nanolatches. The significantly reduced fluorescence intensity observed for the fully complementary MH nanolatch, relative to the mismatched MT nanolatch, demonstrates both the selectivity (**b**) and rapid kinetics (**c**) of loop formation. **d**, Agarose gel electrophoresis analysis confirming loop formation using the nanolatch strategy. A distinct loop complex band is observed only with a fully complementary nanolatch. Source data

To detect sequence variations, each oligo pool includes a short overhang adjacent to the target site, which recruits a DNA hairpin probe or ‘nanolatch’ via a toehold-mediated strand displacement. The hairpin structure blocks simultaneous binding of multiple nanolatches to the overhang and target site, thus preventing competing interactions that could disrupt target recognition. Once captured by the overhang, the nanolatch unfolds to expose a complementary sequence that dynamically approaches the target site, enabling the formation of a metastable loop. Depending on the strength of base pairing between the nanolatch and the target site, the loop exists in either a latched state (where strong complementarity fastens the loop) or an unlatched state (where weak binding leads to loop opening). As the carrier translocates through a ~10 nm nanopore, these states produce distinct ionic current patterns: a pronounced spike comparable in depth to the carrier signal drop (Extended Data Fig. 1c) indicates successful loop formation, whereas its absence signals failure of loop formation (Extended Data Fig. 1f). A separate, smaller signal (green) from built-in reference structures aids in event alignment and interpretation (Extended Data Fig. 1 and Supplementary Fig. 1). The formation of the loop structure was also verified by bulk fluorescence experiments and gel electrophoresis (Extended Data Fig. 2).

## Results

### Detection of nucleotide mutations

**Fig. 2:**
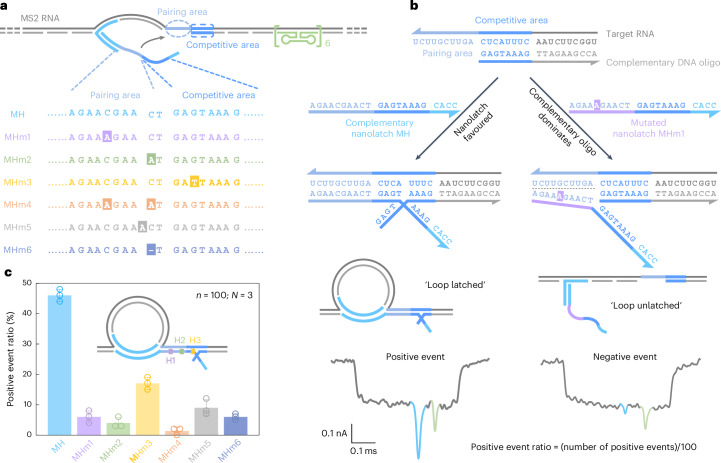
Detection of nucleotide mutations on engineered nanolatches. **a**, A sketch of the MS2 carrier design and sequence comparison between the complementary nanolatch (MH) and its six mutated variants (MHm1–MHm6), highlighting nucleotide changes in both pairing and competitive areas that interact with the target site on the MS2 carrier. **b**, The mechanism of the competitive interaction between the MS2 RNA target site and nanolatches/complementary DNA oligo, illustrated using the fully complementary nanolatch MH and single-nucleotide mutated nanolatch MHm1. The representative nanopore current traces exhibit distinct signatures corresponding to the ‘loop latched’ (positive event) and ‘loop unlatched’ (negative event) states. The positive event ratio is defined as the number of positive events divided by the total number of events per measurement (*n* = 100). **c**, An analysis of positive event ratios for nanolatches MH–MHm6. The bar charts present positive event ratios from three independent measurements (*N* = 3) for each nanolatch. The data are shown as the mean ± standard deviation. Source data

Due to the limited availability of long RNA samples with defined mutations, we first validated RNA-SCAN using MS2 RNA as a model carrier scaffold (Supplementary Fig. 2) and synthetic nanolatches carrying specific base changes (Fig. 2a). Specifically, we designed six nanolatch variants targeting the MS2 RNA site, each carrying a distinct sequence alteration: three with single-nucleotide substitutions at different positions (MHm1–MHm3), one with two substitutions (MHm4), one with an insertion (MHm5) and one with a deletion (MHm6). Each nanolatch was incubated separately with the carrier at a tenfold stoichiometric excess (Supplementary Fig. 3). The resulting constructs were analysed using nanopore measurements.

**Extended Data Fig. 3:**
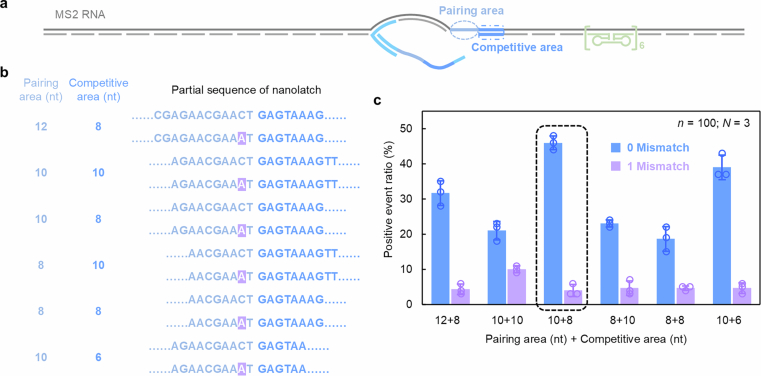
Optimization of pairing and competitive area lengths at the target site. **a**, Schematic of the MS2 carrier design. **b**, Design parameters for each target site, including pairing and competitive area lengths and the corresponding nanolatch sequences. **c**, Nanopore measurement results for all target site designs, each measured with a fully complementary and a mismatched nanolatch, respectively. The greatest discrimination between non-mismatched and mismatched sequences is observed with a pairing area of 10 nt and a competitive area of 8 nt. Data are presented as mean ± standard deviation from three independent measurements. Source data

The sensitivity of RNA-SCAN to single-nucleotide variations stems from the finely tuned competitive hybridization mechanism at the target site (Fig. 2b). Specifically, each target site is engineered with a 10-nt pairing area and an adjacent 8-nt competitive area. When assembling the carrier, the competitive area of the RNA scaffold is complemented by a DNA oligo, while the pairing area is intentionally left single-stranded to allow full nanolatch binding. This strategic design establishes a delicate competition where the target RNA can transiently hybridize with either the nanolatch or the complementary DNA oligo. When the RNA sequence fully matches the nanolatch, their interaction is thermodynamically favourable, yielding latched loop structures, which manifest as characteristic spike signatures in nanopore measurements (Fig. 2b, left). However, even a single-nucleotide mismatch weakens the nanolatch-RNA binding affinity, tipping the balance in favour of the complementary oligo, which forms a more stable duplex with the RNA scaffold. This competitive displacement of nanolatch increases the likelihood of an unlatched state, characterized by the absence of the large current spike associated with the latched configuration (Fig. 2b, right). We classify translocation events as ‘positive’ when the characteristic spike is present and ‘negative’ when it is absent. A current threshold of 0.55 (Extended Data Fig. 1) was applied to facilitate classification. The positive event ratio is defined as the number of positive events out of 100 total recorded events per nanopore measurement. Optimization of the pairing area and competitive area is shown in Extended Data Fig. 3.

**Extended Data Fig. 4:**
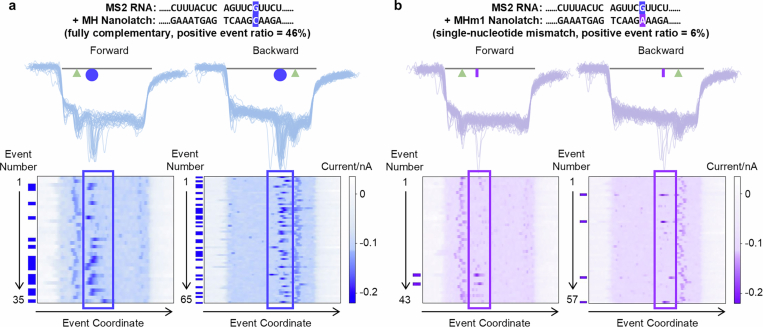
Overlaid nanopore current traces and two-dimensional current intensity plots from the first 100 linear events recorded for MS2 carriers interacting with the fully complementary nanolatch (MH, a) and a mismatched nanolatch (taking MHm1 as the example, b). Data are selected from the three independent measurements of each design whose positive event ratio is closest to the average value. All events are divided into forward and backward translocation groups and are normalized separately by event duration for presentation. All events are aligned to the initial current drop, indicating the entry of the carrier into the nanopore. In the carrier schematic, the same colors as those in Fig. 2 are used to present different structures. The grey line indicates the carrier backbone, the green triangle represents the reference structure, the blue circle refers to a latched loop formed on MS2 carrier with a fully complementary nanolatch, and the short purple line refers to an unlatched sensing overhang without a loop formed. In the two-dimensional plots, colors indicate the magnitude of current drops. The small reference structure usually appears as a light band, while the large spike resulting from loop formation produces a dark-colored band. Negative (unlatched) events lack the loop-associated dark bands. The rectangles on the left of each plot represent the positive events where loops are formed. The clear signature of an additional large spike in the MH group compared to the MHm1 group in the overlaid current traces confirms the more stable loop formation with fully complementary MH than with mismatched MHm1. This is further supported by a greater number of dark bands in the two-dimensional plots of MH. The asymmetric arrangement of carrier structures likely results in the more backward translocation events than forward translocation events. Source data

Figure 2c shows the positive event ratios calculated from the first 100 linear nanopore events (*n* = 100) in each experiment, with error bars from three independent repeats (*N* = 3). This standardized quantification ensures reliable yet efficient analysis across conditions (Supplementary Fig. 4). When the nanolatch sequence was fully complementary to the MS2 RNA target site (MH), the positive event ratio approached 50%. This level reflects the dynamic nature of loop formation and represents a balance optimized for single-nucleotide sensitivity. In the presence of a single-nucleotide mismatch, the positive event ratio consistently dropped, though the magnitude of reduction depended on the mutation’s position. Mutations in the pairing area (MHm1 and MHm2) led to an approximate tenfold decrease, with positive event ratios falling to ~5%, while the change in the competitive area (MHm3) resulted in a higher average ratio of ~17%. This is probably because mutations in the competitive area primarily affect the competition between the nanolatch and the complementary oligo without disrupting the initial nanolatch-RNA pairing, thereby allowing loop formation to proceed more readily. To ensure high sensitivity and specificity of RNA-SCAN, we strategically positioned mutated or chemically modified nucleotides within the pairing area in subsequent designs. Two mismatches (MHm4) further suppressed loop formation, almost approaching the detection limit. Nucleotide insertion (MHm5) or deletion (MHm6) in the nanolatch also disrupted its interaction with the carrier, reducing the positive event ratio to below 10%. These results demonstrate that RNA-SCAN is highly sensitive to subtle sequence variations and can discriminate single-base differences through easily interpretable nanopore signatures (Extended Data Fig. 4).

### Detection of nucleotide modifications

Having demonstrated the ability of RNA-SCAN to detect single-nucleotide mutations, we next applied this methodology to epigenetic nucleotide modifications, which play a critical role in many fundamental metabolic functions^3,4^.

**Fig. 3:**
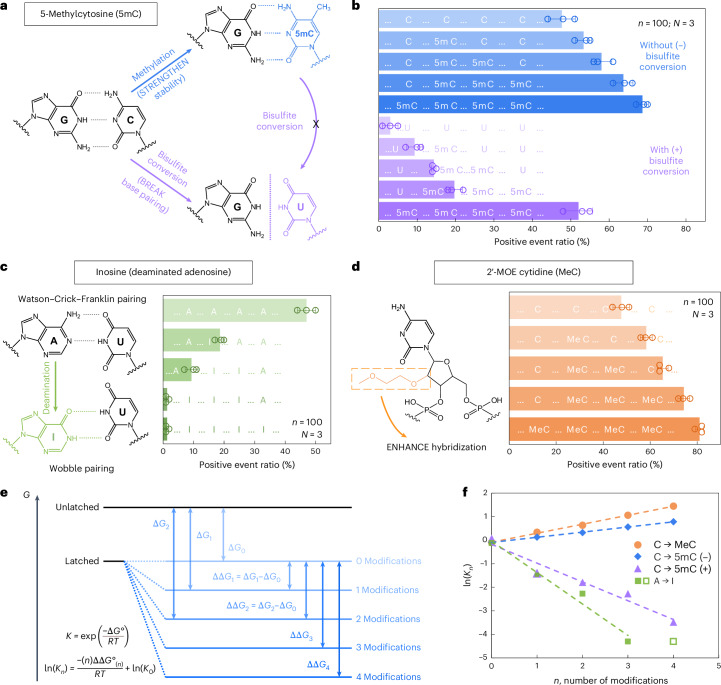
Detection of modifications on nanolatches. **a**, A schematic depicting how 5mC modification influences C/G base pairing before and after bisulfite conversion. The MS2 carrier design is identical to that in Fig. 2. **b**, The nanopore measurement results of MS2 carriers incubated with nanolatches containing 0–4 5mC modifications, with (+) and without (−) bisulfite treatment. **c**, A schematic of how inosine modification affects A/U base pairing, along with nanopore measurement results for nanolatches containing 0–4 inosines in the sequence. **d**, A schematic of MeC modification and nanopore measurement results for nanolatches possessing 0–4 MeCs. The data in **b**–**d** are shown as the mean ± standard deviation from three independent measurements. **e**, A theoretical model of the nanolatch system for detecting modifications. The presence of modifications can either strengthen or weaken the stability of the duplex between the nanolatch and the RNA scaffold, altering the energy difference $\Delta G$ between ‘latched’ and ‘unlatched’ energy levels. *R* is the ideal gas constant, *T* is the absolute temperature at which the reaction occurs, ${K}_{n}$ is the equilibrium constant of loop latching, and $n$ is the number of modifications. **f**, The linear fitting results of $\mathrm{ln}({K}_{n})$ against $n$ for each type of modifications studied in this work. 5mC (+) and 5mC (−) refer to cytosine methylations with (+) and without (−) bisulfite treatment, respectively. The data point for four inosines (open square) was excluded from fitting, as the positive event ratio had already dropped to nearly 0% with three inosines. Source data

We first examined 5-methylcytosine (denoted as 5mC in DNA and m^5^C in RNA), a key epigenetic marker frequently observed in cancer^21,22^. To investigate its presence and abundance in nucleic acids, we evaluated the RNA-SCAN system from two complementary directions. First, the covalent introduction of a methyl group to cytosine alters the van der Waals radii around the C5 position and modifies base stacking potential. Prior studies have shown that such methylations can enhance duplex stability by influencing thermodynamic properties^23,24^. Alternatively, bisulfite treatment can selectively deaminate unmethylated cytosines to uracil while leaving 5-methylcytosines intact^25,26^. This chemical conversion alters the base-pairing preference of cytosine from guanine to adenine, thereby disrupting hybridization patterns (Fig. 3a). Following these principles, we designed five nanolatch variants containing 0–4 5mC modifications and measured loop formation with and without bisulfite conversion. The nanopore measurement results are summarized in Fig. 3b. Without bisulfite conversion, increasing the number of 5mC modifications led to higher positive event ratios, reaching ~69% for the four-modification nanolatch, which is nearly 1.5 times the ratio observed with the unmodified control (~48%). With bisulfite conversion, the positive event ratio for the four-methylated nanolatch remained relatively high at ~52%, whereas nanolatches containing fewer 5mC modifications exhibited a substantial decrease in positive events. This pattern suggests that bisulfite-induced deamination of unmethylated cytosines disrupted their pairing with guanines, destabilizing loop formation and leading to unlatching.

We next tested the ability of RNA-SCAN to detect inosine, a product of adenosine deamination that is associated with RNA editing and post-transcriptional gene regulation^27,28^. Chemically, this amino-to-keto conversion alters the base’s hydrogen bonding properties, transforming adenosine from a hydrogen bond donor into an acceptor. As a result, canonical Watson–Crick–Franklin base pairing with uridine is replaced by two weaker, non-canonical wobble hydrogen bonds^29,30^ (Fig. 3c, left). This change destabilizes the RNA/DNA duplex and alters loop formation within the carrier. To evaluate this effect, we designed five nanolatches containing 0–4 inosine substitutions. Nanopore measurements (Fig. 3c, right) showed that even a single inosine substitution reduced the positive event ratio from ~47% to ~19%. With three inosines present, loop formation was almost completely disrupted, and the signal dropped to near zero. These results confirm that RNA-SCAN can sensitively detect the structural consequences of inosine incorporation.

To assess RNA-SCAN’s ability to detect sugar modifications, we incorporated 2′-*O*-methoxyethyl (2′-MOE) groups into cytidines (2′-MOE cytidine, MeC), which are known to enhance duplex stability in antisense oligo therapeutics^31,32^ (Fig. 3d, left). Substituting 5mC with MeC in nanolatches led to even greater positive signals (Fig. 3d, right): four MeC modifications yielded ~81% positive events, compared with ~69% with 5mC and ~48% with unmodified sequences. Even a single MeC increased signal by over 10%, highlighting this modification’s strong stabilizing effect on base pairing.

To interpret these findings, we modelled loop formation as a two-state energy system: latched versus unlatched (Fig. 3e). Modifications shift the energy of the latched state, altering its equilibrium. Based on the nearest-neighbour model^33,34^, which assumes additive energetic contributions from adjacent base pairs, we observed strong linear correlations between the number of modifications and the equilibrium constant (Fig. 3f). These trends aligned with our experimental results and support the thermodynamic basis for modification detection. Further details are available in Supplementary Note 2.

### Discrimination of *E. coli* and *S*. Typhi by RNA-SCAN

To evaluate the performance of RNA-SCAN on naturally occurring long RNA, we applied it to differentiate between *Escherichia coli* and *Salmonella enterica* serovar Typhi (*S*. Typhi), two closely related Gram-negative bacteria of clinical relevance. While most *E. coli* strains are harmless gut commensals, some pathogenic variants cause severe gastrointestinal illness^35,36^. *S*. Typhi, a human-specific pathogen, is responsible for enteric (typhoid) fever and presents distinct antimicrobial resistance profiles compared with *E. coli*^37,38^.

**Fig. 4:**
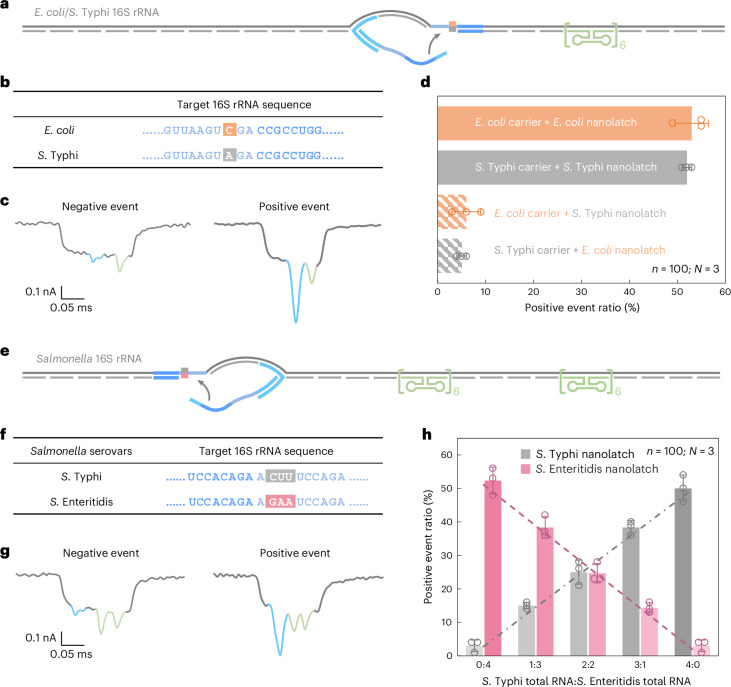
Detection and quantification of *E. coli* and *Salmonella* species based on nucleotide variations in their 16S rRNA sequences. **a**, A schematic of the *E. coli*/*S*. Typhi 16S rRNA carrier design with a reference structure positioned on one side of the carrier and a target site located at the centre. **b**, The sequences of *E. coli* and *S*. Typhi 16S rRNA at the target site, highlighting a single-nucleotide variation. **c**, Representative nanopore current traces of the *E. coli*/*S*. Typhi 16S rRNA carrier. **d**, The analysis of the positive event ratio for the four combinations of *E. coli*/*S*. Typhi carrier with *E. coli*/*S*. Typhi nanolatch. The carrier concentration was set at 0.3 nM at the start of nanopore measurements and the nanolatch concentration at 3 nM for both *E. coli* and *S*. Typhi. **e**, A schematic of the *Salmonella* 16S rRNA carrier design with two reference structures placed on one side and a target site on the other side. **f**, The sequences of *S*. Typhi and *S*. Enteritidis 16S rRNA at the target site, highlighting three continuous different bases. **g**, Representative nanopore current traces of the *Salmonella* 16S rRNA carrier. **h**, An analysis of the positive event ratio for total RNA mixtures of *S*. Typhi and *S*. Enteritidis at defined input ratios, using either the *S*. Typhi or *S*. Enteritidis nanolatch. The combined concentration of *S*. Typhi and *S*. Enteritidis 16S rRNA carriers was maintained at 0.3 nM in all nanopore measurements, with a constant nanolatch concentration of 3 nM. The data in **d** and **h** are shown as mean ± standard deviation from three independent measurements. Source data

To enable species-level discrimination, we focused on 16S ribosomal RNA (rRNA), a highly conserved but diagnostically informative bacterial identity marker. Sequence alignment across all 14 operons from both species revealed >99% intraspecies similarity and 97–98% interspecies similarity (Supplementary Fig. 5). Within this context, we identified an 18-nt region containing a consistent single-nucleotide difference between *E. coli* and *S*. Typhi 16S rRNA and selected it as the target for RNA-SCAN detection (Fig. 4a,b). Separate oligo pools were designed to assemble RNA carriers from the total 16S rRNA of each species, while nanolatches were tailored to match the species-specific variant of the target sequence. Each carrier was incubated with either a matched or mismatched nanolatch, creating four experimental combinations.

**Extended Data Fig. 5:**
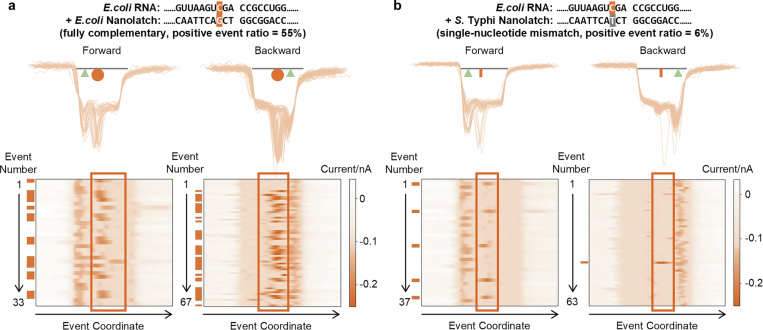
Overlaid nanopore current traces and two-dimensional current intensity plots from the first 100 linear events recorded for *E. coli* carriers interacting with the fully complementary *E. coli* nanolatch (a) and the mismatched *S*. Typhi nanolatch (b). The orange circle refers to the latched loop formed on the *E. coli* carrier with the fully complementary *E. coli* nanolatch, while the short orange line refers to the sensing overhang in the absence of loop formation when using the mismatched *S*. Typhi nanolatch. Source data

Nanopore measurements were conducted using total RNA directly extracted from *E. coli* and *S*. Typhi cultures, without additional purification or enrichment. In matched combinations—*E. coli* carrier with *E. coli* nanolatch or *S*. Typhi carrier with *S*. Typhi nanolatch—the positive event ratio exceeded 50%, indicating successful loop formation (Fig. 4d). By contrast, the mismatched combinations yielded positive event ratios of around 5%, demonstrating a clear loss of nanolatch binding. This nearly tenfold difference in signal underscores the high sensitivity and specificity of RNA-SCAN in distinguishing single-nucleotide differences within long, native RNA molecules (Extended Data Fig. 5). Importantly, the assay requires no enzymatic processing, amplification or fragmentation and works directly with total bacterial RNA, making it highly suitable for practical applications such as microbial identification and resistance profiling.

### Quantification of *Salmonella* 16S rRNA variants

While the high sequence similarity of 16S rRNA between *E. coli* and *S*. Typhi already poses a challenge for conventional assays, different serovars of *Salmonella enterica*, such as *S*. Typhi and *S*. Enteritidis^39^, are even more difficult to distinguish, with 16S rRNA sequence identities exceeding 99.5% overlap (Supplementary Fig. 6).

**Extended Data Fig. 6:**
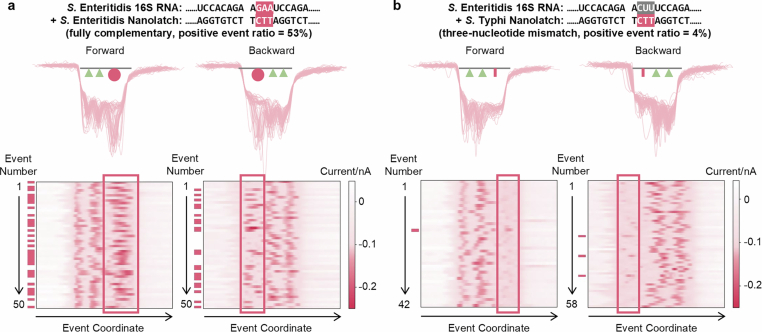
Overlaid nanopore current traces and two-dimensional current intensity plots from the first 100 linear events recorded for *S*. Enteritidis carriers interacting with the fully complementary *S*. Enteritidis nanolatch (a) and the mismatched *S*. Typhi nanolatch (b). These events feature two small reference spikes in the current trace. The pink circle refers to the latched loop formed on the *S*. Enteritidis carrier with the fully complementary *S*. Enteritidis nanolatch, while the short pink line refers to the sensing overhang in the absence of loop formation when using the mismatched *S*. Typhi nanolatch. Source data

To address this, we designed a shared oligo pool that binds conserved regions in both *S*. Typhi and *S*. Enteritidis 16S rRNA, using consensus nucleotides at polymorphic sites. The carrier design included two reference structures on one side to distinguish this construct from earlier *E. coli*/*S*. Typhi carriers (Fig. 4e and Extended Data Fig. 6) and a nanolatch binding site on the other side where three consecutive nucleotides differ between the two serovars (Fig. 4f). To mimic clinical conditions where RNA from multiple bacterial species may coexist, we mixed total RNA extracted from *S*. Typhi and *S*. Enteritidis in defined ratios. After hybridizing the mixture with the oligo pool to generate carriers for both serovars, each mixture was split and incubated with either a *S*. Typhi-specific or *S*. Enteritidis-specific nanolatch.

The nanopore measurements showed that the positive event ratio increased linearly with the proportion of each target RNA in the mixture when probed with its matching nanolatch (Fig. 4h). This linear relationship confirms that RNA-SCAN can not only distinguish between highly similar sequences but also quantify their relative abundance in complex RNA backgrounds. The small error bars and consistent fits further indicate that other minor sequence variations or intraspecies polymorphisms did not interfere with detection. These results highlight the potential of RNA-SCAN for multiplexed pathogen quantification in mixed samples, with single-nucleotide resolution and without requiring prior sample purification or amplification.

### m^5^C detection in 16S rRNA from *E. coli* and *A. baumannii*

**Fig. 5:**
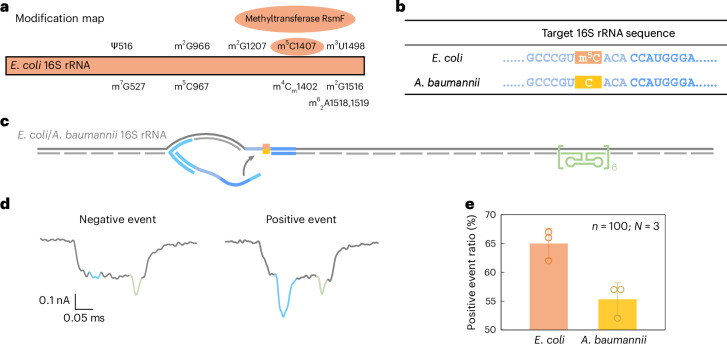
Detection of m^5^C modification on *E. coli* 16S rRNA compared with *A. baumannii* 16S rRNA. **a**, A modification map of *E. coli* 16S rRNA, highlighting the essential role of the methyltransferase RsmF in catalysing methylation at cytosine 1407. **b**, The sequences of *E. coli* and *A. baumannii* 16S rRNA at the target site. The cytosine at position 1407 is methylated in *E. coli* 16S rRNA but remains unmethylated in *A. baumannii* 16S rRNA. **c**, A schematic of the *E. coli*/*A. baumannii* 16S rRNA carrier design, featuring a reference structure on one side of the carrier and the target site at the opposite end. **d**, Representative nanopore current traces of the *E. coli*/*A. baumannii* 16S rRNA carrier. **e**, The analysis of the positive event ratio for *E. coli* and *A. baumannii* 16S rRNA carriers using the same nanolatch. The carrier concentration was maintained at 0.3 nM and the nanolatch concentration at 3 nM. The higher positive event ratio observed for *E. coli* can be attributed to the stabilizing effect of the m^5^C modification at the target site. The data are shown as mean ± standard deviation from three independent measurements. Source data

Beyond sequence variation, we further evaluated the ability of RNA-SCAN ability to detect base modifications in endogenous long RNA. In *E. coli*, rRNAs contain at least 24 known methylation sites essential for ribosomal function^40^. Among these, three are m^5^C modifications located at positions C967 and C1407 in 16S rRNA (Fig. 5a) and at C1962 in 23S rRNA. Notably, methylation at C1407 is highly conserved in *E. coli* and has been implicated in translation fidelity^40,41^. By contrast, *Acinetobacter baumannii*, a common cause of healthcare-associated opportunistic infections, completely lacks the methyltransferase gene responsible for C1407 methylation^42,43^. This makes *E. coli* and *A. baumannii* ideal comparative models for studying RNA methylation at this position.

Given the substantial sequence divergence of over 15% between their 16S rRNA operons (Supplementary Fig. 7), we designed species-specific oligo pools for assembling RNA carriers, while using a common nanolatch targeting the C1407 region. The resulting carrier structure included the C1407 target site on one side and a reference structure on the other (Fig. 5b,c). The total RNA extracted directly from bacterial cells was used without further processing.

**Extended Data Fig. 7:**
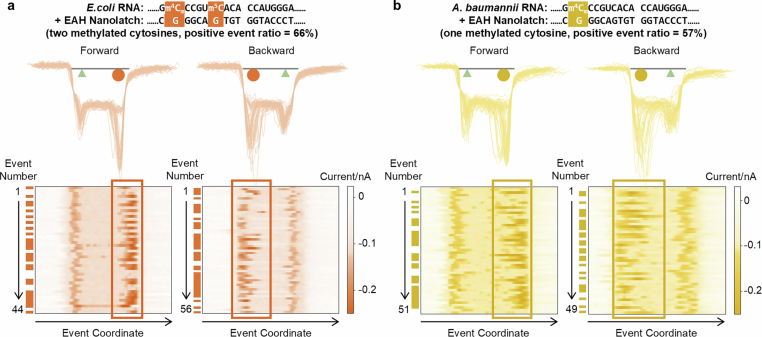
Overlaid nanopore current traces and two-dimensional current intensity plots from the first 100 linear events recorded for *E. coli* (with m^5^C modification at the target site, a) and *A. baumannii* (without modification, b) carriers, both measured with their shared nanolatch design. The orange circle refers to the latched loop formed on the *E. coli* carrier using the EAH nanolatch. Two methylated cytosines are present in the target RNA region. The yellow circle refers to the latched loop formed on the *A. baumannii* carrier using the same nanolatch. Only one methylated cytosine is present in the target RNA region. Source data

**Extended Data Fig. 8:**
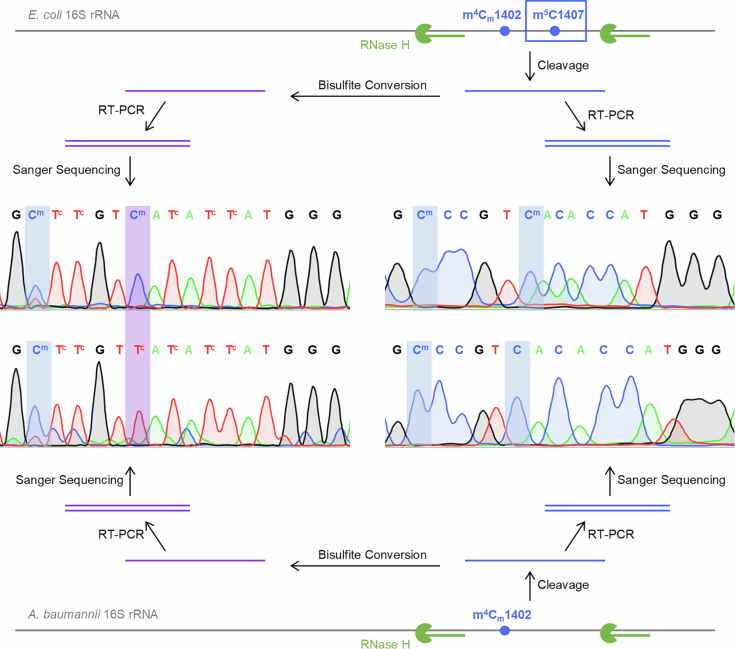
Detection of methylated cytosines in *E. coli* and *A. baumannii* 16S rRNA by bisulfite-treated RNA-seq and Sanger sequencing. 16S rRNA from *E. coli* and *A. baumannii* are first cleaved by RNase H enzyme near the target site. The cleavage products are then divided into two groups, with one group subjected to bisulfite conversion and the other retaining the native sequence. Subsequently, both groups undergo reverse transcription followed by forty cycles of PCR amplification and are sent for Sanger sequencing. The sequencing results confirm the presence of the m^5^C1407 modification on *E. coli* 16S rRNA, which is absent on *A. baumannii* 16S rRNA. However, both species contain the m^4^C_m_1402 modification on their 16S rRNA. C^m^ denotes methylated cytosines, and T^c^ refers to thymines converted from unmethylated cytosines during bisulfite treatment. Source data

Nanopore measurements showed a statistically significant difference in loop formation between species. *E. coli* produced a positive event ratio of 65% ± 2%, compared with 55% ± 2% for *A. baumannii* (Fig. 5e and Extended Data Fig. 7), with a *t*-test yielding *P* = 0.01289. These results are consistent with our earlier MS2-based experiments, supporting the interpretation that m^5^C1407 enhances nanolatch binding by stabilizing C/G base pairing. Interestingly, both species exhibited higher signal levels than unmodified MS2 carriers. To investigate this, we performed bisulfite sequencing on the 16S rRNA target region. The results (Extended Data Fig. 8) suggest that another nearby modification, m^4^C_m_1402 (a 2′-*O*-methylated and *N4*-methylated cytosine found in both species), is partly resistant to bisulfite conversion and may similarly strengthen base pairing.

Together, these findings demonstrate the capability of RNA-SCAN to detect single-base RNA modifications in native, unamplified samples. Moreover, the observation of enhanced signals from nearby modified nucleotides suggests that RNA-SCAN could also serve as a tool for identifying previously unannotated modifications in functionally important RNA regions.

## Conclusion

In this study, we develop RNA-SCAN, a nanopore-based single-molecule detection strategy that enables sequence-specific analysis of long RNA molecules without amplification or labelling. By integrating programmable DNA nanostructures with glass nanopore sensing, RNA-SCAN transforms subtle RNA sequence variations into binary signal outputs, allowing direct, high-specificity detection of single-nucleotide changes in native RNA samples.

In addition to mutations, RNA-SCAN sensitively detects nucleotide modifications with distinct biophysical effects. Modifications such as 5mC and MeC enhance C/G pairing, increasing loop formation and positive event ratios. By contrast, deamination of cytosine and adenosine (to uracil and inosine) disrupts canonical base pairing, reducing signal output.

We validated mutation detection in native RNA by targeting a single-nucleotide difference in 16S rRNA between *E. coli* and *S*. Typhi, achieving a tenfold signal contrast between matched and mismatched nanolatch-carrier pairs. RNA-SCAN also enabled quantification of *Salmonella* subspecies in mixed RNA samples, with the positive event ratios linearly correlating with species abundance. Beyond sequence variation, we identified endogenous m^5^C at position 1407 in *E. coli* 16S rRNA, using *A. baumannii* as an unmethylated control. Higher signal levels in both species compared with MS2 suggested additional stabilizing modifications, such as m^4^C_m_1402, potentially contributing to loop formation. These findings demonstrate the ability of RNA-SCAN to detect and even reveal unknown base modifications in long, complex RNA molecules.

Unlike electro-optical nanopore platforms that require synchronized fluorescence and electrical readouts^44,45^, RNA-SCAN achieves sequence-specific detection using purely resistive pulse signals. Compared with previous glass nanopore methods^16,18^, it operates without pore modification and relies on a binary current change, thereby avoiding reliance on variable parameters such as spike depth or duration. In contrast to isoform identification approaches that require transcript processing^46^, RNA-SCAN analyses total cellular RNA directly. Compared wtih nanoswitch sensors^47,48^, which often target short fragments, our method enables direct detection of mutations and modifications in long RNA molecules by fully utilizing conserved sequences through programmable nanolatch binding. This makes RNA-SCAN more suitable for complex clinical samples, where fragmentation and background interference are common. More importantly, RNA-SCAN also provides a conceptual link between DNA nanotechnology and nanopore sensing: its modular design framework, combining spatially programmable duplex scaffolds with functional molecular switches, may be extended to other single-molecule detection applications beyond RNA. These include protein–nucleic acid interactions, RNA–RNA base-pairing dynamics or targeted sensing of RNA in non-aqueous environments. In this sense, RNA-SCAN offers not only a specific analytical solution but also a versatile design principle for nanoscale molecular interrogation.

Despite these strengths, several limitations merit attention. First, the current platform relies on single-target detection per nanopore and per run, which constrains throughput. While the assay is scalable in principle, future development of parallelized multinanopore arrays and multiplexed nanolatch designs will be necessary to enable high-content profiling of multiple RNA features within a single experiment. Second, while our current design targets predefined sites, detection of unknown mutations or modifications requires integration with broader sequence-scanning strategies or upstream enrichment. Furthermore, loop formation sensitivity varies with local sequence context and may require calibration to account for duplex thermodynamic heterogeneity.

As portable, high-throughput nanopore devices continue to mature, RNA-SCAN has potential to evolve into a point-of-care platform for detecting clinically relevant RNA sequence variations and modifications, particularly in infectious disease diagnostics, cancer monitoring and epitranscriptomic studies.

## Methods

### Materials

The DNA oligos were purchased from Integrated DNA Technologies. MS2 RNA (0.8 μg μl^−1^) was purchased from Sigma-Aldrich (#10165948001). *E. coli* DH5α total RNA (1 μg μl^−1^) was purchased from Thermo Fisher Scientific (#AM7940). *Salmonella* and *A. baumannii* total RNA were extracted from bacteria cultured in the laboratory. Specifically, the total RNA concentrations obtained were as follows: 801 ng μl^−1^ for *S*. Typhi, 697 ng μl^−1^ for *S*. Enteritidis and 592 ng μl^−1^ for *A. baumannii*. The Fast Bisulfite Conversion Kit was purchased from Abcam (#ab117127). The EZ RNA Methylation Kit was purchased from Zymo Research (#R5001). The RNase H enzyme (#M0297S), the OneTaq RT-PCR Kit (#E5310S) and the Monarch Spin DNA Gel Extraction Kit (#T1120S) were bought from New England Biolabs. The RNAprotect Cell Reagent (#76526) and the RNeasy Mini Kit (#74104) were bought from Qiagen. The commercial buffers used in this study were Tris–EDTA buffer solution 100× concentrate (Sigma-Aldrich, #T9285), UltraPure 1 M Tris–HCl (Thermo Fisher Scientific, #15568025), 1 M MgCl_2_ (Thermo Fisher Scientific, #AM9530G) and nuclease-free water (Thermo Fisher Scientific, #AM9932). The chemicals included lithium chloride for molecular biology ≥99% purity (Sigma-Aldrich, #L9650) and lysozyme (Sigma-Aldrich, #10837059001).

### Design and synthesis of MS2 carriers

A total of 84 DNA oligos, referred to as staple M_1 to staple M_84, were designed to complement the MS2 RNA sequence for linearization. Equal amounts of each staple were mixed before carrier preparation, and their sequences are listed in Supplementary Table 1. For the designs used in this work, the staples at the reference and target sensing sites were replaced with dumbbell and probe strands, as detailed in Supplementary Tables 2 and 3. Specifically, staples 64–66 were replaced by oligos MD1–MD6 to form the reference structure. To form loops, staple M_47 was replaced by probe MO47, while staples M_51 and M_52 were replaced by strands MP51* and MP52*.

To prepare the carriers, we first made staple mixtures according to the designs shown in Fig. 2 and Supplementary Tables 2 and 3. A 40 μl reaction was then prepared by combining MS2 RNA (20 nM) and the staple mixture (100 nM) in 100 mM LiCl and 1× TE buffer (10 mM Tris–HCl, 1 mM EDTA, pH 8.0), with nuclease-free water added to reach the final reaction volume. The solution was heated to 70 °C and then subjected to a linear cooling ramp to 25 °C over 45 min. The excess oligos were removed by centrifuging the solution at 9,000*g* for 10 min using Amicon Ultra 100 kDa filter units with washing buffer (10 mM Tris–HCl, 0.5 mM MgCl_2_, pH 8.0). This washing step was repeated twice. Typically, 30 μl of purified carrier solution could be collected after the washing steps. A quantification was performed using a NanoDrop 2000 spectrophotometer. The prepared carrier solutions were then stored in a fridge at 4 °C for later use.

### Bisulfite treatment of nanolatches with 5mC modifications

A total of 2 µl of 50 ng µl^−1^ nanolatch was used in each experiment. The bisulfite treatment of nanolatches was performed using the Fast Bisulfite Conversion Kit following the manufacturer’s protocol, except for that the washing step for converted DNA was repeated four times instead of the suggested two. The modified nanolatches were eluted using 15 µl of Elution Solution and stored at −20 °C.

### Extraction of *Salmonella* and *A. baumannii* total RNA

The bacterial cultures were grown overnight in Luria–Bertani medium. A 500 µl aliquot of the overnight culture was mixed with 1 ml of RNA protect reagent to stabilize the RNA. The mixture was then treated with lysozyme at a final concentration of 15 mg ml^−1^ for 20 min to digest the bacterial cell wall. The RNA extraction was subsequently performed using the RNeasy Mini Kit according to the manufacturer’s protocol. The concentration and purity of the isolated total RNA were evaluated using a Nanodrop 2000 spectrophotometer.

### Design and synthesis of bacterial 16S rRNA carriers

Two sets of 35 DNA oligos were designed for the *E. coli* and *S*. Typhi 16S rRNA oligo pools: staples E_1 to E_35 (Supplementary Table 4) and staples T_1 to T_35 (Supplementary Table 5), respectively. Although the sequences of these two oligo pools are quite similar, they exhibit a difference of approximately 2.8%. When constructing the carriers, dumbbell strands ETD1–ETD6 were used to replace staples E_7–E_9 and T_7–T_9 in the *E. coli* and *S*. Typhi oligo pools, respectively, to form the reference structure. For the loop design, staples E_20 and T_20 were replaced with probe ETO20, while E_15 and T_15 were replaced with ETP15. In addition, E_17 and T_17 were replaced with EP17 and TP17. The sequences of these oligos are listed in Supplementary Table 6. As *Salmonella* subspecies *S*. Typhi and *S*. Enteritidis have highly conserved 16S rRNA sequences, with a difference of less than 0.5%, a shared oligo pool was designed for both *S*. Typhi and *S*. Enteritidis (Supplementary Table 7). This pool includes dumbbell strands, with staples TE_18–TE_23 and TE_29–TE_34 forming two reference structures. To create the loop, staple TE_10 was replaced with probe TEO10 (Supplementary Table 8). To detect the methylation state of C1407 on *E. coli* and *A. baumannii* 16S rRNA, two separate oligo pools were designed for each of them due to the big difference in their sequences of over 15% (Supplementary Tables 9 and 10). Dumbbell structures were formed at staples 28–33, and loops were formed at staples 3–7 (Supplementary Table 11). After mixing the staples of the corresponding carriers, total RNA sample and the staple mixture were added in 100 mM LiCl and 1× TE buffer. The following carrier synthesis steps were the same as those used for MS2 carriers.

### RNA-seq library preparation

Two DNA oligos (ERH1 and ERH2 for *E. coli* or ARH1 and ARH2 for *A. baumannii*) were hybridized to the 16S rRNA near the target C1407 position by incubating the mixture at 70 °C for 5 min with a fivefold excess of oligos. RNase H enzyme was then added to cleave the RNA/DNA hybrid while leaving unhybridized single-stranded RNA intact. Following cleavage, the remaining RNA oligos were separated into two groups. One group served as the negative control, retaining the original sequence. The other group underwent bisulfite conversion using the RNA Methylation Kit adhering to the manufacturer’s protocol with a modification of performing the final washing step four times instead of two. Reverse transcription followed by polymerase chain reaction (PCR) was performed on both groups using the OneTaq RT-PCR Kit. PCR amplification was carried out for 40 cycles. Two sets of primers were used: one for bisulfite-treated RNA (EPB1 and EPB2 for *E. coli* or APB1 and APB2 for *A. baumannii*) and another for untreated RNA (EPO1 and EPO2 for *E. coli* or APO1 and APO2 for *A. baumannii*). The PCR products were analysed by 2.5% agarose gel electrophoresis. The product bands were excised from the gel under ultraviolet light and the samples were extracted using the Monarch Spin DNA Gel Extraction Kit. After assessing sample purity using a NanoDrop 2000 spectrophotometer, the PCR fragments were sent to the Cambridge Biochemistry Sequencing Team for Sanger sequencing.

### Nanopore fabrication

Glass nanopores were fabricated by laser-assisted pulling (P-2000, Sutter Instrument) of quartz capillaries (outer diameter 0.5 mm and inner diameter 0.2 mm, World Precision Instruments). The resulting nanopores were then fixed into a PDMS chip. After plasm cleaning the chip for 5 min, the I-V curves were measured for each nanopore in 4 M LiCl buffer (in 1× TE buffer, pH 9.0) to assess their sizes before the addition of samples. A previous study^16^ has demonstrated that the diameter of a nanopore and the current measured at 600 mV in 4 M LiCl are roughly equal in value. The nanopores with a current of no larger than 13 nA at 600 mV and a root mean square noise below 7 pA were selected for measurements in this work. The parameters of all the nanopores used in this work are listed in Supplementary Note 3.

### Nanopore measurement

Before conducting nanopore measurements, 0.01 pmol of the carrier, prepared using the above-mentioned protocol, was mixed with 0.1 pmol of the relevant nanolatch and incubated at room temperature for 1 h. The optimal concentration ratio of the nanolatch to the carrier is presented in Supplementary Fig. 3, while the designs and sequences of the hairpins used can be found in Supplementary Tables 3, 6, 8 and 11. Following incubation, the mixture was diluted in 4 M LiCl buffer to achieve a carrier concentration of 0.3 nM. Subsequently, 15 μl of the mixture (4.5 fmol of carrier, equivalent to approximately 5.5 ng of MS2 RNA or 2.5 ng of bacterial 16S rRNA) was added to the tip side of the glass nanopore. Two electrodes were placed on opposite sides of the nanopore, and a potential of 600 mV was applied to drive the negatively charged carrier molecules through the nanopore. This process resulted in characteristic transient changes in the ionic current trace. Using the standard sample concentration of 0.3 nM, it typically took about 1–2 h to collect sufficient useful events using a single nanopore for analysis of each measurement. For comparing two similar samples, separate nanopore measurements were required. However, these could be conducted simultaneously using two identical setups.

### Nanopore data analysis

The current signal was recorded and amplified using an Axopatch 200B patch clamp amplifier (Axon Instruments) at a sampling rate of 1 MHz and filtered by an external low-pass Bessel filter (Model 900CT, Frequency Devices) at 50 kHz. A data acquisition card (DAQ card, PCIe-6251 or PCIe-6351, National Instruments) realized the conversion from analogue to digital signal with 16-bit resolution. Analysis of the recorded current traces was performed with LabVIEW software and Python programs, which usually took about 30 min. By strategically replacing certain staples in the oligo pool for our designs, we could predict the locations of reference spike(s) and loop spike(s) along the event traces. To ensure the accuracy and efficiency of our nanopore measurements, we discarded folded events and linear events without identifiable reference spikes and limited our analysis to the first 100 events in each measurement. Events with small reference spike(s) and large loop spike(s) were classified as positive events. By contrast, events lacking the large spikes were classified as negative events. The positive event ratio in the first 100 linear events was calculated as ‘number of positive events × 100%’. More details about nanopore data processing are provided in Supplementary Note 4.

### Agarose gel electrophoresis and fluorescence measurement

The stock solutions of C1–C4 and L1–L3 were initially diluted to a concentration of 20 µM in TM buffer, where T is 10 mM Tris–HCl and M is 10 mM MgCl_2_ (pH 7.5), while strands MH and MT were diluted to 1 µM. To prepare reaction group 1 (G1), 2 µl each of C1, C2 and L3 were mixed with 34 µl of TM buffer. Similarly, reaction group 2 (G2) was created by mixing 2 µl each of L1, L2, C3 and C4 with 32 µl of TM buffer. The G1 and G2 mixtures were then separately heated to 90 °C for 5 min, followed by a linear cooling ramp to 50 °C over 24 min. Circular or linear DNA structures shown in Extended Data Fig. 2 were prepared by mixing 10 µl each of G1 and G2 with 10 µl of MH/MT and TM buffers, resulting in a final DNA concentration of 0.33 µM. All the resulting solutions were then slowly cooled to 20 °C over 21 min. For agarose gel characterization, 10 μl of each final reaction liquid were mixed with 2 µl of 6× DNA loading buffer and loaded into a 2.5% agarose gel for electrophoresis analysis. The agarose gel was prepared in 1× TAE buffer and contained 0.07 μl of GelRed per millilitre. The gel was run at 120 V for 60 min and visualized under ultraviolet light.

Fluorescence emission intensity of the DNA complexes G1 + G2 + MH and G1 + G2 + MT at 520 nm with an excitation of 495 nm in a 10 mm path-length quartz cuvette were separately collected using a Cary Eclipse fluorescence spectrophotometer (Agilent Technologies). The the two samples were diluted to 50 nM by TM buffer before the fluorescence measurement.

## Online content

Any methods, additional references, Nature Portfolio reporting summaries, source data, extended data, supplementary information, acknowledgements, peer review information; details of author contributions and competing interests; and statements of data and code availability are available at 10.1038/s41565-025-01965-6.

## Supplementary information

Supplementary Information Supplementary Figs. 1–7, Tables 1–11, Notes 1–4 and References.

Supplementary Note 1 Statistical source data for Supplementary Figs. 3 and 4.

Supplementary Note 2 Gel image for Supplementary Fig. 2.

## Source data

Source Data Fig. 2 Statistical source data.

Source Data Fig. 3 Statistical source data.

Source Data Fig. 4 Statistical source data.

Source Data Fig. 5 Statistical source data.

Source Data Extended Data Fig. 1 Statistical source data.

Source Data Extended Data Fig. 2 Fluorescence data.

Source Data Extended Data Fig. 2 Gel image.

Source Data Extended Data Fig. 3 Statistical source data.

Source Data Extended Data Fig. 4 Source data for nanopore events.

Source Data Extended Data Fig. 5 Source data for nanopore events.

Source Data Extended Data Fig. 6 Source data for nanopore events.

Source Data Extended Data Fig. 7 Source data for nanopore events.

Source data Extended Data Fig. 8 Sequencing data.

## Data Availability

The data supporting the results of this study are available in this article and its Supplementary Information. Additional raw data are available at 10.17863/CAM.118402. Source data are provided with this paper.

## Extended data

are available for this paper at 10.1038/s41565-025-01965-6.
