# Supplementary material for: Nanopore detection of single-nucleotide RNA mutations and modifications with programmable nanolatches
Source: Nat Nanotechnol. 2025 Jun 27;20(10):1473–81. doi: 10.1038/s41565-025-01965-6 (PMC12534182; doi:10.1038/s41565-025-01965-6)
Supplement: Supplementary file 1 — Supplementary Figs. 1–7, Tables 1–11, Notes 1–4 and References. [file 41565_2025_1965_MOESM1_ESM.pdf]

nature nanotechnology

Supplementary information https://doi.org/10.1038/s41565-025-01965-6

Nanopore detection of single-nucleotide RNA mutations and modifications with programmable nanolatches

In the format provided by the authors and unedited

Table of Contents

Supplementary Figures

Supplementary Figure 1. Structures and dimensions of the latched loop and the six-dumbbell reference in nanopore.
Supplementary Figure 2. Agarose gel electrophoresis showing the efficiency of RNA/DNA hybrid complex formation.
Supplementary Figure 3. Dependence of positive event ratio on the concentration of the MH nanolatch relative to the MS2 carrier, measured in nanopore.
Supplementary Figure 4. Cumulative kinetics of positive event ratio in RNA-SCAN measurement using MS2 RNA carrier and its fully complementary nanolatch MH.
Supplementary Figure 5. Sequence alignment of *E. coli* and *S.* Typhi 16S rRNA.
Supplementary Figure 6. Sequence alignment of *S.* Typhi and *S.* Enteritidis 16S rRNA.
Supplementary Figure 7. Sequence alignment of *E. coli* and *A. baumannii* 16S rRNA.

Supplementary Tables

Supplementary Table 1. MS2 RNA oligo pool.
Supplementary Table 2. Dumbbell oligos used to form the reference structure on MS2 carrier.
Supplementary Table 3. Oligos used to form loop structures on MS2 carrier.
Supplementary Table 4. *E. coli* 16S rRNA oligo pool used for the discrimination with *S.* Typhi.
Supplementary Table 5. *S.* Typhi 16S rRNA oligo pool used for the discrimination with *E. coli*.
Supplementary Table 6. Oligos used for the discrimination between *E. coli* and *S.* Typhi 16S rRNA.
Supplementary Table 7. *Salmonella* 16S rRNA oligo pool used for the quantification of *S.* Typhi and *S.* Enteritidis.
Supplementary Table 8. Oligos used for the quantification of *S.* Typhi and *S.* Enteritidis.
Supplementary Table 9. *E. coli* 16S rRNA oligo pool used for the detection of m^5C on 16S rRNA.
Supplementary Table 10. *A. baumannii* 16S rRNA oligo pool used for the detection of m^5C on 16S rRNA.
Supplementary Table 11. Oligos used for the detection of m^5C in o6S rRNA of *E. coli* and *A. baumannii*.

Supplementary Notes

1. Nanopore Sensing Principle
2. Modeling and Calculations
3. Nanopore Measurement Details
4. Nanopore Data Processing

Supplementary References

Supplementary Figures

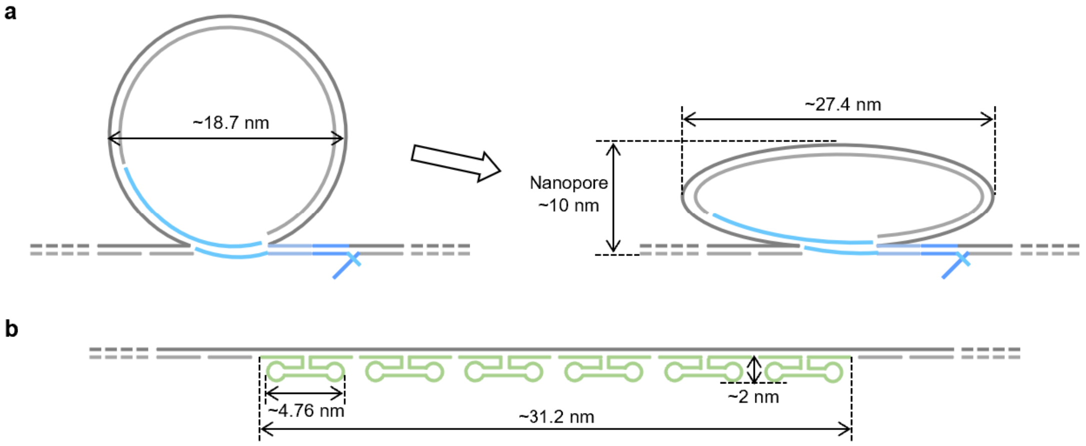

Supplementary Figure 1. Structures and dimensions of the latched loop and the six-dumbbell reference in nanopore. The RNA scaffold of the carrier forms A-form RNA/DNA hybrids when base-paired with complementary DNA oligos, overhangs, or dumbbells. In contrast, the structures within dumbbells and the overhang-nanolatch interaction regions adopt the B-form dsDNA conformation. The A-form RNA/DNA hybrid is assumed to have a helical rise of 0.26 nm per base pair along the axis, with a double-helix diameter of 2.6 nm, while the B-form dsDNA has a helical rise of 0.34 nm per base pair and a double-helix diameter of 2.0 nm. **a**, The latched loop structure, approximately 190 bp in length, behaves like free circles with an estimated ~18.7 nm diameter in solution under unrestricted conditions. However, when translocating through a ~10 nm nanopore, the loop is compressed into an oval shape, with its long axis extending to approximately ~27.4 nm due to spatial constraints. This large and highly flexible structure can displace a substantial volume of solution inside the nanopore, significantly affecting the ionic flow through the nanopore and generating a pronounced current signal. **b**, The six-dumbbell reference structure spans ~31.2 nm along the carrier, with a height of the dumbbells measuring ~2 nm. The 4-dT loops in the dumbbells are simplified as 2 bp duplexes. Due to its compact nature, this structure has a much smaller impact on ionic flow within the nanopore, leading to a current signal that is approximately half that of the loop structure.

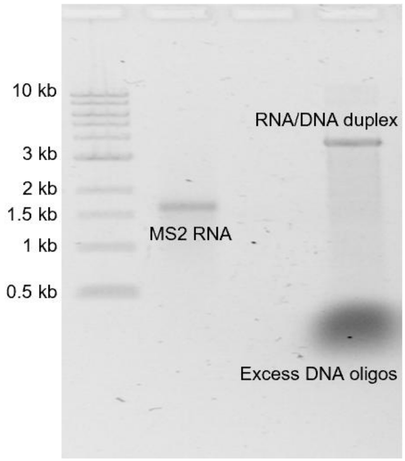

Supplementary Figure 2. Agarose gel electrophoresis showing the efficiency of RNA/DNA hybrid complex formation. The lane corresponding to the MS2 RNA carrier shows a complete disappearance of the single-stranded MS2 RNA band, indicating that the majority of the MS2 RNA has been successfully hybridized with complementary DNA oligos. A distinct band representing the double-stranded RNA/DNA duplex is visible, along with a band for excess DNA oligos. The absence of residual single-stranded RNA signal and the clear presence of the double-stranded RNA/DNA duplex confirm the high efficiency of hybridization, consistent with the designed RNA/DNA interactions.

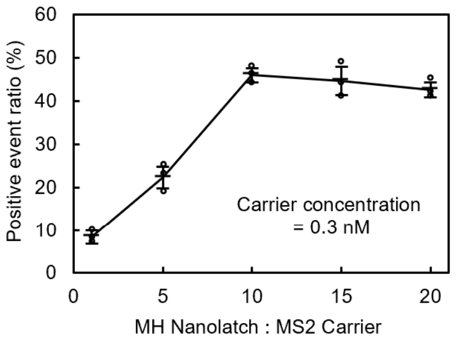

Supplementary Figure 3. Dependence of positive event ratio on the concentration of the MH nanolatch relative to the MS2 carrier, measured in nanopore. The concentration of MS2 carrier at the start of nanopore measurements remained constant at 0.3 nM. When the concentration of nanolatch is ten times of the concentration of carrier, the positive event ratio reaches its maximum value. Data are shown as mean ± SD from three independent measurements.

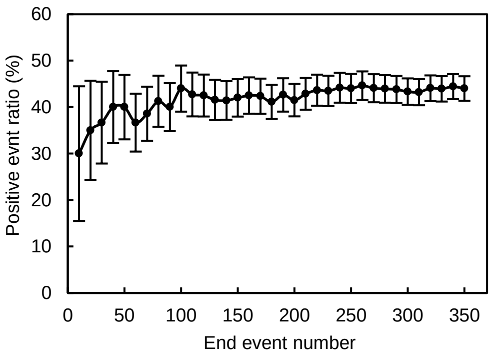

Supplementary Figure 4. Cumulative kinetics of positive event ratio in RNA-SCAN measurement using MS2 RNA carrier and its fully complementary nanolatch MH. The analysis begins with the first 10 events, and the positive event ratio is updated cumulatively every 10 additional events. Each data point in the plot represents the observed proportion of positive events at a given checkpoint, starting from event 10, 20, and so on. Error bars represent the standard error (SE) of the observed proportion at each checkpoint, calculated using the formula $SE = \sqrt{\frac{p_{obs}(1-p_{obs})}{N}}$, where p_{obs} is the observed proportion of positive events, and N is the cumulative number of events at that checkpoint. Initial fluctuations in the ratio are expected due to the limited number of events. As more data accumulates, the positive event ratio progressively stabilizes. After approximately 100 events, the ratio reaches a steady value, supporting the reliability of the selected event cutoff for downstream analysis.

a **Percent Identity Matrix – created by Clustal2.1**

1:	*E. coli* 1	100.00	99.16	99.16	99.81	99.16	99.29	99.16	97.46	97.46	97.46	97.46	97.46	97.46	97.46
2:	*E. coli* 2	99.16	100.00	100.00	98.97	100.00	99.03	99.61	97.20	97.20	97.20	97.20	97.20	97.20	97.20
3:	*E. coli* 3	99.16	100.00	100.00	98.97	100.00	99.03	99.61	97.20	97.20	97.20	97.20	97.20	97.20	97.20
4:	*E. coli* 4	99.81	98.97	98.97	100.00	98.97	99.49	99.23	97.52	97.52	97.52	97.52	97.52	97.52	97.52
5:	*E. coli* 5	99.16	100.00	100.00	98.97	100.00	99.03	99.61	97.20	97.20	97.20	97.20	97.20	97.20	97.20
6:	*E. coli* 6	99.29	99.03	99.03	99.49	99.03	100.00	99.29	98.04	98.04	98.04	98.04	98.04	98.04	98.04
7:	*E. coli* 7	99.16	99.61	99.61	99.23	99.61	99.29	100.00	97.59	97.59	97.59	97.59	97.59	97.59	97.59
8:	*S.* Typhi 1	97.46	97.20	97.20	97.52	97.20	98.04	97.59	100.00	99.87	99.87	99.87	99.87	100.00	100.00
9:	*S.* Typhi 2	97.46	97.20	97.20	97.52	97.20	98.04	97.59	99.87	100.00	100.00	100.00	100.00	99.87	99.87
10:	*S.* Typhi 3	97.46	97.20	97.20	97.52	97.20	98.04	97.59	99.87	100.00	100.00	100.00	100.00	99.87	99.87
11:	*S.* Typhi 4	97.46	97.20	97.20	97.52	97.20	98.04	97.59	99.87	100.00	100.00	100.00	100.00	99.87	99.87
12:	*S.* Typhi 5	97.46	97.20	97.20	97.52	97.20	98.04	97.59	99.87	100.00	100.00	100.00	100.00	99.87	99.87
13:	*S.* Typhi 6	97.46	97.20	97.20	97.52	97.20	98.04	97.59	100.00	99.87	99.87	99.87	99.87	100.00	100.00
14:	*S.* Typhi 7	97.46	97.20	97.20	97.52	97.20	98.04	97.59	100.00	99.87	99.87	99.87	99.87	100.00	100.00

b

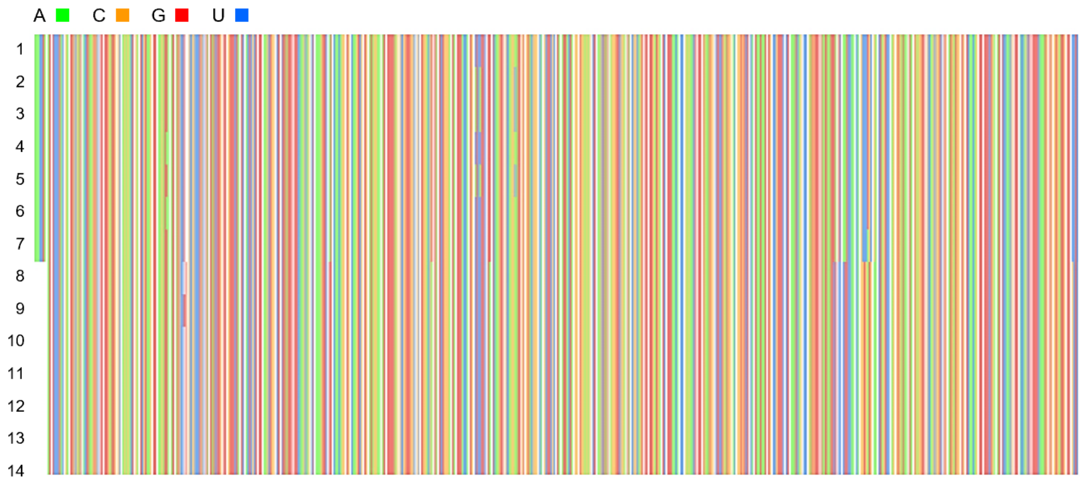

Supplementary Figure 5. Sequence alignment of *E. coli* and *S.* Typhi 16S rRNA. a, Percent Identity Matrix comparing the seven *E. coli* 16S rRNA sequences with the seven *S.* Typhi 16S rRNA sequences. **b**, Visual representation of the sequence alignment generated using Clustal2.1.

a **Percent Identity Matrix – created by Clustal2.1**

1:	*S.* Typhi 1	100.00	99.87	99.87	99.87	99.87	100.00	100.00	99.48	99.48	99.54	99.48	99.48	99.48	99.48
2:	*S.* Typhi 2	99.87	100.00	100.00	100.00	100.00	99.87	99.87	99.48	99.48	99.54	99.48	99.48	99.48	99.48
3:	*S.* Typhi 3	99.87	100.00	100.00	100.00	100.00	99.87	99.87	99.48	99.48	99.54	99.48	99.48	99.48	99.48
4:	*S.* Typhi 4	99.87	100.00	100.00	100.00	100.00	99.87	99.87	99.48	99.48	99.54	99.48	99.48	99.48	99.48
5:	*S.* Typhi 5	99.87	100.00	100.00	100.00	100.00	99.87	99.87	99.48	99.48	99.54	99.48	99.48	99.48	99.48
6:	*S.* Typhi 6	100.00	99.87	99.87	99.87	99.87	100.00	100.00	99.48	99.48	99.54	99.48	99.48	99.48	99.48
7:	*S.* Typhi 7	100.00	99.87	99.87	99.87	99.87	100.00	100.00	99.48	99.48	99.54	99.48	99.48	99.48	99.48
8:	*S.* Enteritidis 1	99.48	99.48	99.48	99.48	99.48	99.48	99.48	100.00	100.00	99.94	100.00	100.00	100.00	99.87
9:	*S.* Enteritidis 2	99.48	99.48	99.48	99.48	99.48	99.48	99.48	100.00	100.00	99.94	100.00	100.00	100.00	99.87
10:	*S.* Enteritidis 3	99.54	99.54	99.54	99.54	99.54	99.54	99.54	99.94	99.94	100.00	99.94	99.94	99.94	99.94
11:	*S.* Enteritidis 4	99.48	99.48	99.48	99.48	99.48	99.48	99.48	100.00	100.00	99.94	100.00	100.00	100.00	99.87
12:	*S.* Enteritidis 5	99.48	99.48	99.48	99.48	99.48	99.48	99.48	100.00	100.00	99.94	100.00	100.00	100.00	99.87
13:	*S.* Enteritidis 6	99.48	99.48	99.48	99.48	99.48	99.48	99.48	100.00	100.00	99.94	100.00	100.00	100.00	99.87
14:	*S.* Enteritidis 7	99.48	99.48	99.48	99.48	99.48	99.48	99.48	99.87	99.87	99.94	99.87	99.87	99.87	100.00

b

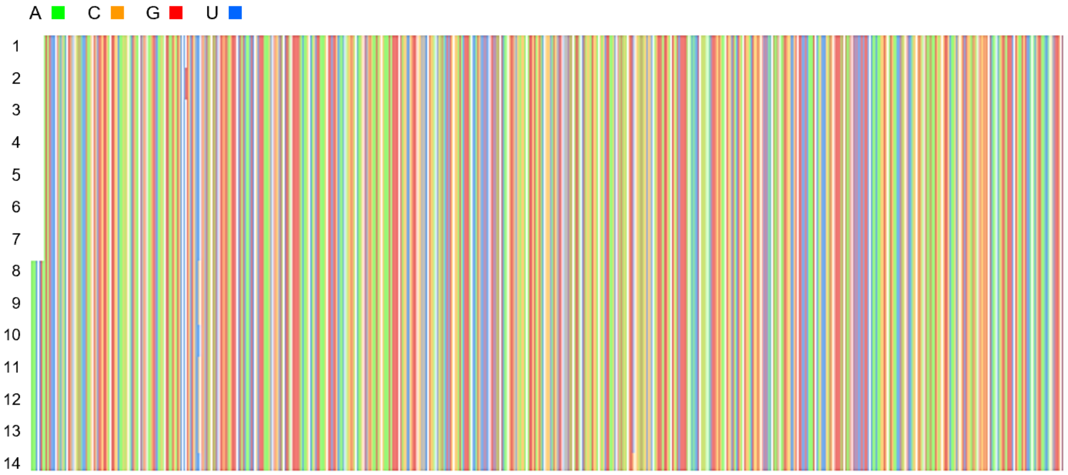

Supplementary Figure 6. Sequence alignment of *S.* Typhi and *S.* Enteritidis 16S rRNA. a, Percent Identity Matrix comparing the seven *S.* Typhi 16S rRNA sequences with the seven *S.* Enteritidis 16S rRNA sequences. **b**, Visual representation of the sequence alignment generated using Clustal2.1.

a **Percent Identity Matrix – created by Clustal2.1**

1:	*E. coli* 1	100.00	99.16	99.16	99.81	99.16	99.29	99.16	84.51	84.51	84.51	84.51	84.51	84.51
2:	*E. coli* 2	99.16	100.00	100.00	98.97	100.00	99.03	99.61	84.64	84.64	84.64	84.64	84.64	84.64
3:	*E. coli* 3	99.16	100.00	100.00	98.97	100.00	99.03	99.61	84.64	84.64	84.64	84.64	84.64	84.64
4:	*E. coli* 4	99.81	98.97	98.97	100.00	98.97	99.49	99.23	84.57	84.57	84.57	84.57	84.57	84.57
5:	*E. coli* 5	99.16	100.00	100.00	98.97	100.00	99.03	99.61	84.64	84.64	84.64	84.64	84.64	84.64
6:	*E. coli* 6	99.29	99.03	99.03	99.49	99.03	100.00	99.29	85.09	85.09	85.09	85.09	85.09	85.09
7:	*E. coli* 7	99.16	99.61	99.61	99.23	99.61	99.29	100.00	84.77	84.77	84.77	84.77	84.77	84.77
8:	*A. baumannii* 1	84.51	84.64	84.64	84.57	84.64	85.09	84.77	100.00	99.93	99.93	99.93	99.93	99.93
9:	*A. baumannii* 2	84.51	84.64	84.64	84.57	84.64	85.09	84.77	99.93	100.00	100.00	100.00	100.00	100.00
10:	*A. baumannii* 3	84.51	84.64	84.64	84.57	84.64	85.09	84.77	99.93	100.00	100.00	100.00	100.00	100.00
11:	*A. baumannii* 4	84.51	84.64	84.64	84.57	84.64	85.09	84.77	99.93	100.00	100.00	100.00	100.00	100.00
12:	*A. baumannii* 5	84.51	84.64	84.64	84.57	84.64	85.09	84.77	99.93	100.00	100.00	100.00	100.00	100.00
13:	*A. baumannii* 6	84.51	84.64	84.64	84.57	84.64	85.09	84.77	99.93	100.00	100.00	100.00	100.00	100.00

b

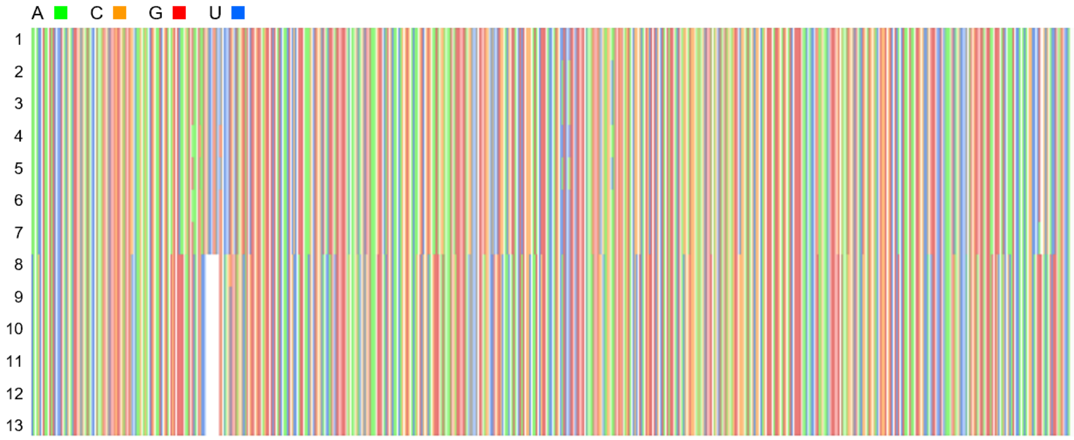

Supplementary Figure 7. Sequence alignment of *E. coli* and *A. baumannii* 16S rRNA. a, Percent Identity Matrix comparing the seven *E. coli* 16S rRNA sequences with the seven *A. baumannii* 16S rRNA sequences. **b**, Visual representation of the sequence alignment generated using Clustal2.1.

Supplementary Tables

Supplementary Table 1. MS2 RNA oligo pool.

Name	Sequence
M_1	TGGGTGGTAA CTAGCCAAGC AGCTAGTTAC CAAATCGGGA GAATCCCGGG
M_2	TCCTCTCTTT AGGGGGAGGT CCCTGGGAAG AAGCCCGCCC ACCTTTCGGT
M_3	GGAGCCGGAC CGCTTTCGCA CCCGTGCTCT TTCGAGCACA CCCACCCCGT
M_4	TTACGGGGGT CCCTCGGTCA GCTACCGAGG AGAGCTCGCT GGCCCACACT
M_5	CCTGAGGGAA TGTGGGAACC GGCGTTAGCC ACTCCGAAGT GCGTATAACG
M_6	CGCACGCCGG CGGACTTCAT GCTGTCGGTG ATTTCACCTC CAGTATGGAA
M_7	CCACGCTATG TAGCGACCAC TGTCGTGCTT TTCGCTGAAG AACTTGCGTT
M_8	CTCGAGCGAT ACGAGCAAGA CGGAAACCCG AGGTACGGGT ATCCGCGAGC
M_9	AGCCGCCCGT ACGGAGTCTT GGTGTATACC GAGACTGCCG TAGGCGGGCT
M_10	GACTACGTAG TAGTCGGCAG CGAGGTCCGT CCCACCGTTG AACATCGTTG
M_11	GCACCTGGGA GGTGTGCCGT ACCCACACCT TATAGAGGCG TGGATCTGAC
M_12	ATACCTCCGA CAACTCCCCA ACCCCGTAGC CGATTTAATA TCAGCATCAG
M_13	GGCGAAGAGA TTGTCAACAG GTTTCTTGAT GTAAAACGGT TTGACATCGA
M_14	CACCACGGTA AAAGTGCGCG CCGCAGCTCT CGCGAAAGAG CCCGGACACG
M_15	AACGTTTTAC GAAGATTCGG TTTAAAACCG TAGTAGGCAA GTGCCTCTAG
M_16	CACACGGGGT GCAATCTCAC TGGGACATAT AATATCGTCC CCGTAGATGC
M_17	CTATGGTTCC GGCGTTACCA AAATGGATTT GGGTCGCTTT GACTATTGCC
M_18	CAGAATATCA TGGACTCTAG CTCAAATGTG AACCCATTTC CCATTGTGGA
M_19	AAATAGTTCC CATCGTATCG TCTCGCCATC TACGATTCCG TAGTGTGAGC
M_20	GGATACGATC GAGATATGAA TATAGCTCTG GTGGGAGAAA ACTCCACACC
M_21	AGGCGATCGG AGATGGAATC GGATGCAGAC GATAAGTCTA TCGTCGCAAG
M_22	CGAACCATCT ACGCTGCCCT GCTGAGCCAG ACGCTGGTTG ATCGATTGAT
M_23	CATTCAGGTC TATACCAACG GATTTGAGCC GGCGTCTGAT GAAAGCACCG
M_24	ACCCCTTTCT GGAGGTACAT ATTCATATCA GGCTCCTTAC
M_25	AGGCAGCCCG ATCTATTTTA TTATTCTTCG GAACTGTAAA
M_26	CACTCCGTTC CCTACAACGA GCCTAAATTC ATATGACT
M_27	CGTTATAGCG GACCGCGTGT CTGATCCACG GCGCACAT
M_28	TGGTCTCGGA CCAATAGAGC CGCTCTCAGA GCGCGGGG
M_29	GGTAACGGTT GCTTGTTCAG CGAACTTCTT GTAAGGCG
M_30	CTGCATCCTG CAACTTGTGC CCCATAGGAG CACCGTTG
M_31	GAGAACGTGC ATTGCCCAAA CAACGTCGAT CGGTAGCC
M_32	AGAGAGGAGG TTGCCAATAA GGCTACGGAT GCTGGTTT
M_33	GTAAAACATC CGGATCCCAT GACAAGGATT TGTCATGT
M_34	AAGAAACCTT CTCTATTTAT CTGACCGCGA TCACCATT
M_35	CGCCTCCCGT AGCTTAGCGA TAGCTAAGGT ACGACGGGTC
M_36	GCCTCGTCAT TACCAGAACC TAAGGTCGGA TGCTTTGTGA
M_37	GCAATTCGTC CCTTAAGTAA GCAATTGCTG TAAAGTCGTC
M_38	ACTGTGCGGA TCACCGCTTC CAGTAGCGAC AG

M_39	AAGCAATTGA TTGGTAAATT TCGAGAGAAA GATCGCGA
M_40	GGAAGATCAA TACATAAAGA GTTGAACTTC TTTGTTGT
M_41	CTTCGACATG GGTAATCCTC ATGTTTGAAT GGCCGGCG
M_42	TCTATTAGTA GATGCCGGAG TTTGCTGCGA TTGCTGAG
M_43	GGAATCGGGT TTCCATCTTT TAGGAGACCT TGCATTGC
M_44	CTTAACAATA AGCTCGCAGT CGGAATTCGT AGCGAAAA
M_45	TTGGAATGGT TAGTTCCATA TTTAAGTACG AACGCCAT
M_46	GCGGCTACAG GAAGCTCTAC ACCACCAACA GTCTGGGT
M_47	TGCCACTTTA GGCACCTCGA CTTTGATGGT GTATTTGC
M_48	GATTCTGCGC AGAGCTCTGA CGAACGCTAC AGGTTACT
M_49	TTGTAAGCCT GTGAACGCGA GTTAGAGCTG ATCCATTC
M_50	AGCGACCCCG TTAGCGAAGT TGCTTGGGGC GACAGTCA
M_51	CGTCGCCAGT TCCGCCATTG TCGACGAGAA CGAACTGAGT
M_52	AAAGTTAGAA GCCATGCTTC AAACTCCGGT TGAGGGCTCT
M_53	ATCTAGAGAG CCGTTGCCTG ATTAATGCTA ACGCATCTAA
M_54	GGTATGGACC ATCGAGAAAG GAGACTTTAC GT
M_55	ACGCGCCAGT TGTTGGCCAT ACGGATTGTA CCCCTCGA
M_56	TGCATGGCTG AGATTTGGGC CTTAGCAGTG CCCTGTCT
M_57	CTCCACAGTC CTCCCGTAGG GAGCGTCAAC GCTTATGA
M_58	TGGACTCACC CGTTATTACG TCAGTAACTG TTCCTGAC
M_59	ATGTAGGAGC ATCCCACGGG GGCCGTAAGG CCCTCGAG
M_60	CATGTTACCT ACAGGTAGGA GCCAGTCGAC AACGAATG
M_61	AGAAAGGCAC CTTTTCCCAC ACTATACCTA GTGGGTTC
M_62	AAGATACCTA GAGACGACAA CCATGCCAAA CGTGCATC
M_63	GTTTATGTAA AACCATATCA CGATACGTCG CGATATGT
M_64	TGCACGTTGT CTGGAAGTTT GCAGCTGGAT ACGACAGACG
M_65	GCCATCTAAC TTGATGTTAG TACCGACCTG ACGTACGGCT
M_66	CTCATAGGAA GAAACTCTTG AAGGTGAACC TTCGTAAGCA
M_67	TCTCATATGC ACCCTGGATA TCACTCATTA GT
M_68	GGTAACCAAC CGAACTGCAA CTCCAACCAC CTGCCGGC
M_69	CACGTGTTTT GATCGAAACT TTCGATCTTC GTTTAGGG
M_70	CAAGGTAGCG GAGCGCCTGG CGCCAATTAC CGCGACGA
M_71	GCGGCAGTGT ACGCCTTCAC GAGCGCAATG GTTTGCGT
M_72	CGCGACTTGT GAGGCTGTCG ACCTGGCCTC TGCTAAAG
M_73	CAACACCAAG GTTAAAATTA CCCTGGGTGA CCTTTTGC
M_74	AGGACTTCGG TCGACGCCCG GTTCGCAACG TTCTGCGG
M_75	CACTTCGATG TAAGTCAAGT TTTGGCTTAC AGGGAAGA
M_76	GGCTGTAGCA GGAGCGTGCG TCGAGGGAGA AGCCGAAA
M_77	CCGGCTTTCT CCTCGTACGG GCGACCCCAC GATGACCCAC TTCGCTTGTA G
M_78	GCACCTTGAT CTATCGATGT GACACTTAAC GCCCCCCGTG AATACGGAGA
M_79	GGGGTAGTGC CACTGTTTCG TTTTGGCCCC AGTCGAGTTA AAACGACCGG
M_80	GAGTCCAGTT CGAACGATAT TTTAAAGAGA ATGAGTTATC TTCAGTCTCA
M_81	CCGTCCGCGT AAACGCGAAC GGAGGGGACG AAGGTCTCGT TCTCCCTATC

M_82	AAGGGTACTA AAAGCTCGCA CAGGTCAAAC CTCCTAGGAA TGGAATTCCG
M_83	GCTACCTACA GCGATAGCCA TGGTAGCGTC TCGCTAAAGA CATTAAAAAT
M_84	GGCATTAGCT CGACAGGAAG TTGAGCAGGA CCCCGAAAGG GGTCCCACCC

Supplementary Table 2. Dumbbell oligos used to form the reference structure on MS2 carrier, replacing staples 64-66 in the oligo pool. The dumbbell motifs unbound to the carrier scaffold are marked in red.

Name	Sequence
MD1	TGCACGTTGT TCCTCTTTTGAGGAACAAGTTTTCTTGT CTGGAAGTTT
MD2	GCTGCTGGAT TCCTCTTTTGAGGAACAAGTTTTCTTGT ACGACACACG
MD3	GCCATCTAAC TCCTCTTTTGAGGAACAAGTTTTCTTGT TTGATGTTAG
MD4	TACCGACCTG TCCTCTTTTGAGGAACAAGTTTTCTTGT ACGTACGGCT
MD5	CTCATAGGAA TCCTCTTTTGAGGAACAAGTTTTCTTGT GAAACTCTTG
MD6	AAGGTGAACC TCCTCTTTTGAGGAACAAGTTTTCTTGT TTCGTAAGCA

Supplementary Table 3. Oligos used to form loop structures on MS2 carrier. "P" in the names refers to oligos that are fully complementary to the MS2 scaffold, and "O" refers to overhangs. The numbers in the names of these oligos denote the identifiers of the staples that they replace in the oligo pool. And "MH" refers to hairpin-like nanolatches, with "m" suggesting mutations in the sequences. The pairing regions of overhangs and nanolatches are marked in red. The parts of strands that are complementary to the target sites on MS2 RNA are marked in blue. Mutated or modified nucleotides are highlighted in yellow. Fluorophores are highlighted in green.

Name	Sequence
Loop 1 (formed at staples 47-51 in the oligo pool; 10 nt pairing area+8 nt competitive area)	
MP51	CGTCGCCAGT TCCGCCATTG TCGACG
MP52	GAGTAAAG TTAGAA GCCATGCTTC AAACTCCGGT TGAGGGCTCT
MO47	AAAGCACCTAGCTCAC TTTGCCACTT TAGGCACCTC GACTTTGATG GTGTATTTGC
MH	GTGAGCTAGGTGCTTT T AGAACGAACTGAGTAAAG CACC
MHm	GTGAGCTAGGTGCTTT T AGAACGAAATGAGTAAAG CACC
Optimization of the loop design	
Loop 2 (8 nt pairing area+10 nt competitive area)	
MP51a	CGTCGCCAGT TCCGCCATTG TCGACGAG
MP52a	GAGTAAAGTT AGAA GCCATGCTTC AAACTCCGGT TGAGGGCTCT
MH2	GTGAGCTAGGTGAACT T AACGAACTGAGTAAAGTT CACC
MH2m	GTGAGCTAGGTGAACT T AACGAAATGAGTAAAGTT CACC
Loop 3 (10 nt pairing area+10 nt competitive area)	
MP51	CGTCGCCAGT TCCGCCATTG TCGAGG
MP52a	GAGTAAAGTT AGAA GCCATGCTTC AAACTCCGGT TGAGGGCTCT
MH3	GTGAGCTAGGTGAACT T AGAACGAACTGAGTAAAGTT CACC
MH3m	GTGAGCTAGGTGAACT T AGAACGAAATGAGTAAAGTT CACC
Loop 4 (10 nt pairing area+6 nt competitive area)	
MP51	CGTCGCCAGT TCCGCCATTG TCGACG
MP52b	GAGTAA AGTTAGAA GCCATGCTTC AAACTCCGGT TGAGGGCTCT
MH4	GTGAGCTAGGTGTTAC T AGAACGAACTGAGTAA CACC
MH1_4m	GTGAGCTAGGTGTTAC T AGAACGAAATGAGTAA CACC
Loop 5 (12 nt pairing area+8 nt competitive area)	
MP51b	CGTCGCCAGT TCCGCCATTG TCGA
MP52	GAGTAAAG TTAGAA GCCATGCTTC AAACTCCGGT TGAGGGCTCT
MH5	GTGAGCTAGGTGCTTT T CGAGAACGAACTGAGTAAAG CACC
MH5m	GTGAGCTAGGTGCTTT T CGAGAACGAAATGAGTAAAG CACC
Loop 6 (8 nt pairing area+8 nt competitive area)	
MP51a	CGTCGCCAGT TCCGCCATTG TCGACGAG
MP52	GAGTAAAG TTAGAA GCCATGCTTC AAACTCCGGT TGAGGGCTCT
MH6	GTGAGCTAGGTGCTTT T AACGAACTGAGTAAAG CACC
MH6m	GTGAGCTAGGTGCTTT T AACGAAATGAGTAAAG CACC
Nanolatches with nucleotide mutations of different types and positions from MH.	
MH	GTGAGCTAGGTGCTTT T AGAACGAACTGAGTAAAG CACC
MHm1	GTGAGCTAGGTGCTTT T AGAAAGAACTGAGTAAAG CACC

MHm2	GTGAGCTAGGTGCTTT T AGAACGAAATGAGTAAAG CACC
MHm3	GTGAGCTAGGTGCTTT T AGAACGAACTGATTAAAG CACC
MHm4	GTGAGCTAGGTGCTTT T AGAAAGAAATGAGTAAAG CACC
MHm5	GTGAGCTAGGTGCTTT T AGAACGAAACTGAGTAAAG CACC
MHm6	GTGAGCTAGGTGCTTT T AGAACGAA-TGAGTAAAG CACC
Oligos to study the influence of 5mC modifications on nanolatches	
MP51m	CGTCGCCAGT TCCGCCATTG
MP52m	CGAACTGA GTAAAGTTAGAA GCCATGCTTC AAACTCCGGT TGAGGGCTCT
MO47m	ATATATTATTTAACTCAC TTTGCCACTT TAGGCACCTC GACTTTGATG GTGTATTTGC
MHC0	GTGAGTTAAATAATATAT G TCGACGAGAACGAACTGA ATATATTATT
MH5mC1	GTGAGTTAAATAATATAT G TCGA(5mC)GAGAACGAACTGA ATATATTATT
MH5mC2	GTGAGTTAAATAATATAT G TCGA(5mC)GAGAA(5mC)GAACTGA ATATATTATT
MH5mC3	GTGAGTTAAATAATATAT G TCGA(5mC)GAGAA(5mC)GAA(5mC)TGA ATATATTATT
MH5mC4	GTGAGTTAAATAATATAT G T(5mC)GA(5mC)GAGAA(5mC)GAA(5mC)TGA ATATATTATT
Oligos to study the influence of inosines on nanolatches	
MP51	CGTCGCCAGT TCCGCCATTG TCGACG
MP52	GAGTAAAG TTAGAA GCCATGCTTC AAACTCCGGT TGAGGGCTCT
MO47m	ATATATTATTTAACTCAC TTTGCCACTT TAGGCACCTC GACTTTGATG GTGTATTTGC
MHI1	GTGAGTTAAATAATATAT T AGAACGAACTGAGTAAAG ATATATTATT
MHI1	GTGAGTTAAATAATATAT T AGAACGAICTGAGTAAAG ATATATTATT
MHI2	GTGAGTTAAATAATATAT T AGAACGAICTGIGTAAAG ATATATTATT
MHI3	GTGAGTTAAATAATATAT T AGAICGAICTGIGTAAAG ATATATTATT
MHI4	GTGAGTTAAATAATATAT T AGAICGAICTGIGTAIAG ATATATTATT
Oligos to study the influence of MeC modifications on nanolatches	
MP51m	CGTCGCCAGT TCCGCCATTG
MP52m	CGAACTGA GTAAAGTTAGAA GCCATGCTTC AAACTCCGGT TGAGGGCTCT
MO47m	ATATATTATTTAACTCAC TTTGCCACTT TAGGCACCTC GACTTTGATG GTGTATTTGC
MHC0	GTGAGTTAAATAATATAT G TCGACGAGAACGAACTGA ATATATTATT
MHMeC1	GTGAGTTAAATAATATAT G TCGA(MeC)GAGAACGAACTGA ATATATTATT
MHMeC2	GTGAGTTAAATAATATAT G TCGA(MeC)GAGAA(MeC)GAACTGA ATATATTATT
MHMeC3	GTGAGTTAAATAATATAT G TCGA(MeC)GAGAA(MeC)GAA(MeC)TGA ATATATTATT
MHMeC4	GTGAGTTAAATAATATAT G T(MeC)GA(MeC)GAGAA(MeC)GAA(MeC)TGA ATATATTATT
Oligos for the validation of loop formation by gel electrophoresis and fluorescence experiment	
C1	FAM-AAAGCACCTAGCTCAC TT TGCCACTTTA GGCACCTCGA CTTTGATGGT GTATTTGCGA
C2	TTCTGCGCAG AGCTCTGACG AACGCTACAG GTTACTTTGT AAGCCTGTGA ACGCGAGTTA GAGCTGATC
C3	CATTCAGCGA CCCCGTTAGC GAAGTTGCTT GGGGCGACAG TCACGTCGCC AGTTCCGCCA TTGTCGACG-BHQ1
C4	GAGTAAAG TTAGAAGCCA TGCTTCAAAC TCCGGTTGAG
L1	CTCAACCGGA GTTTGAAGCA TGGCTTCTAA CTTTACTCAGTTCGTTCT CGTCGACAAT GGCGGAACTG GCGACGTGAC TGTCGCCCCA
L2	AGCAACTTCG CTAACGGGGT CGCTGAATGG ATCAGCTCTA ACTCGCGTTC ACAGGCTTAC AAAGTAACC

L3	TGTAGCGTTC GTCAGAGCTC TGCGCAGAAT CGCAAATACA CCATCAAAGT CGAGGTGCCT AAAGTGGCA
MT	GTGAGCTAGGTGCTTT T TTTTTTTTTTTTTTTTTTT CACC

Supplementary Table 4. *E. coli* 16S rRNA oligo pool used for the discrimination with *S.* Typhi.

Name	Sequence
E_1	TGATCCAACC GCAGGTTCCC CTACGGTTAC
E_2	CTTGTTACGA CTTCACCCCA GTCATGAATC ACAAAGTGGT
E_3	AAGCGCCCTC CCGAAGGTTA AGCTACCTAC TTCTTTTGCA
E_4	ACCCACTCCC ATGGTGTGAC GGGCGGTGTG TACAAGGCCC
E_5	GGGAACGTAT TCACCGTGGC ATTCTGATCC ACGATTACTA
E_6	GCGATTCCGA CTTCATGGAG TCGAGTTGCA CACTCCAATC
E_7	CGGACTACGA CGCACTTTAT GAGGTCCGCT TGCTCTCGCG
E_8	AGGTCGCTTC TCTTTGTATG CGCCATTGTA GCACGTGTGT
E_9	AGCCCTGGTC GTAAGGGCCA TGATGACTTG ACGTCATCCC
E_10	CACCTTCCTC CAGTTTATCA CTGGCAGTCT CCTTTGAGTT CCCGGCCGGA
E_11	CCGCTGGCAA CAAAGGATAA GGGTTGCGCT CGTTGCGGGA CTTAACCCAA
E_12	CATTTCACAA CACGAGCTGA CGACAGCCAT GCAGCACCTG TCTCACGGTT
E_13	CCCGAAGGCA CATTCTCATC TCTGAAAACT TCCGTGGATG TCAAGACCAG
E_14	GTAAGGTTCT TCGCGTTGCA TCGAATTAAA CCACATGCTC CACCGCTTGT
E_15	GCGGGCCCCC GTCAATTCAT TTGAGTTTTA ACCTTGCGGC
E_16	CGTACTCCCC AGGCGGTCGA CTTAACGCGT TAGCTCCGGA
E_17	AGCCACGCCT CAAGGGCACA ACCTCCAAGT CGACATCGTT
E_18	TACGGCGTGG ACTACCAGGG TATCTAATCC TGTTTGCTCC
E_19	CCACGCTTTC GCACCTGAGC GTCAGTCTTC GTCCAGGGGG
E_20	CCGCCTTCGC CACCGGTATT CCTCCAGATC TCTACGCATT
E_21	TCACCGCTAC ACCTGGAATT CTACCCCCCT CTACGAGACT CAAGCTTGCC
E_22	AGTATCAGAT GCAGTTCCCA GGTTGAGCCC GGGGATTTCA CATCTGACTT
E_23	AACAAACCGC CTGCGTGCGC TTTACGCCCA GTAATTCCGA TTAACGCTTG
E_24	CACCCTCCGT ATTACCGCGG CTGCTGGCAC GGAGTTAGCC GGTGCTTCTT
E_25	CTGCGGGTAA CGTCAATGAG CAAAGGTATT AACTTTACTC CCTTCCTCCC CGCTGAAAGT
E_26	ACTTTACAAC CCGAAGGCCT TCTTCATACA CGCGGCATGG
E_27	CTGCATCAGG CTTGCGCCCA TTGTGCAATA TTCCCCACTG
E_28	CTGCCTCCCG TAGGAGTCTG GACCGTGTCT CAGTTCCAGT
E_29	GTGGCTGGTC ATCCTCTCAG ACCAGCTAGG GATCGTCGCC
E_30	TAGGTGAGCC GTTACCCCAC CTACTAGCTA ATCCCATCTG GGCACATCCG
E_31	ATGGCAAGAG GCCCGAAGGT CCCCCTCTTT GGTCTTGCGA
E_32	CGTTATGCGG TATTAGCTAC CGTTTCCAGT AGTTATCCCC
E_33	CTCCATCAGG CAGTTTCCCA GACATTACTC ACCCGTCCGC
E_34	CACTCGTCAG CAAAGAAGCA AGCTTCTTCC TGTTACCGTT CGACTTGCAT
E_35	GTGTTAGGCC TGCCGCCAGC GTTCAATCTG AGCCATGATC AAACTCTTCA ATT

Supplementary Table 5. ***S.*** **Typhi 16S rRNA oligo pool used for the discrimination with** ***E. coli*****.**

Name	Sequence
T_1	AAGGAGG TGATCCAACC GCAGGTTCCC CTACGGTTAC
T_2	CTTGTTACGA CTTCACCCCA GTCATGAATC ACAAAGTGGT
T_3	AAGCGCCCTC CCGAAGGTTA AGCTACCTAC TTCTTTTGCA
T_4	ACCCACTCCC ATGGTGTGAC GGGCGGTGTG TACAAGGCCC
T_5	GGGAACGTAT TCACCGTGGC ATTCTGATCC ACGATTACTA
T_6	GCGATTCCGA CTTCATGGAG TCGAGTTGCA CACTCCAATC
T_7	CGGACTACGA CGCACTTTAT GAGGTCCGCT TGCTCTCGCG
T_8	AGGTCGCTTC TCTTTGTATG CGCCATTGTA GCACGTGTGT
T_9	AGCCCTGGTC GTAAGGGCCA TGATGACTTG ACGTCATCCC
T_10	CACCTTCCTC CAGTTTATCA CTGGCAGTCT CCTTTGAGTT CCCGGCCGGA
T_11	CCGCTGGCAA CAAAGGATAA GGGTTGCGCT CGTTGCGGGA CTTAACCCAA
T_12	CATTTCACAA CACGAGCTGA CGACAGCCAT GCAGCACCTG TCTCACAGTT
T_13	CCCGAAGGCA CCAATCCATC TCTGGAAAGT TCTGTGGATG TCAAGACCAG
T_14	GTAAGGTTCT TCGCGTTGCA TCGAATTAAA CCACATGCTC CACCGCTTGT
T_15	GCGGGCCCCC GTCAATTCAT TTGAGTTTTA ACCTTGCGGC
T_16	CGTACTCCCC AGGCGGTCTA CTTAACGCGT TAGCTCCGGA
T_17	AGCCACGCCT CAAGGGCACA ACCTCCAAGT AGACATCGTT
T_18	TACGGCGTGG ACTACCAGGG TATCTAATCC TGTTTGCTCC
T_19	CCACGCTTTC GCACCTGAGC GTCAGTCTTT GTCCAGGGGG
T_20	CCGCCTTCGC CACCGGTATT CCTCCAGATC TCTACGCATT
T_21	TCACCGCTAC ACCTGGAATT CTACCCCCCT CTACAAGACT CAAGCCTGCC
T_22	AGTTTCGAAT GCAGTTCCCA GGTTGAGCCC GGGGATTTCA CATCCGACTT
T_23	GACAGACCGC CTGCGTGCGC TTTACGCCCA GTAATTCCGA TTAACGCTTG
T_24	CACCCTCCGT ATTACCGCGG CTGCTGGCAC GGAGTTAGCC GGTGCTTCTT
T_25	CTGCGGGTAA CGTCAATTGC TGCGGTTATT AACCACAACA CCTTCCTCCC CGCTGAAAGT
T_26	ACTTTACAAC CCGAAGGCCT TCTTCATACA CGCGGCATGG
T_27	CTGCATCAGG CTTGCGCCCA TTGTGCAATA TTCCCCACTG
T_28	CTGCCTCCCG TAGGAGTCTG GACCGTGTCT CAGTTCCAGT
T_29	GTGGCTGGTC ATCCTCTCAG ACCAGCTAGG GATCGTCGCC
T_30	TTGGTGAGCC GTTACCTCAC CAACAAGCTA ATCCCATCTG GGCACATCTG
T_31	ATGGCAAGAG GCCCGAAGGT CCCCCTCTTT GGTCTTGCGA
T_32	CGTTATGCGG TATTAGCCAC CGTTTCCAGT AGTTATCCCC
T_33	CTCCATCAGG CAGTTTCCCA GACATTACTC ACCCGTCCGC
T_34	CACTCGTCAG CAAAGCAGCA AGCTGCTTCC TGTTACCGTT CGACTTGCAT
T_35	GTGTTAGGCC TGCCGCCAGC GTTCAATCTG AGCCATGATC AAACTCT

Supplementary Table 6. Oligos used for the discrimination between *E. coli* and *S.* Typhi 16S rRNA.

Name	Sequence
Dumbbell reference (replacing staples 7-9)	
ETD1	CGGACTACGA TCCTCTTTTGAGGAACAAGTTTTCTTGT CGCACTTTAT
ETD2	GAGGTCCGCT TCCTCTTTTGAGGAACAAGTTTTCTTGT TGCTCTCGCG
ETD3	AGGTCGCTTC TCCTCTTTTGAGGAACAAGTTTTCTTGT TCTTTGTATG
ETD4	CGCCATTGTA TCCTCTTTTGAGGAACAAGTTTTCTTGT GCACGTGTGT
ETD5	AGCCCTGGTC TCCTCTTTTGAGGAACAAGTTTTCTTGT GTAAGGGCCA
ETD6	TGATGACTTG TCCTCTTTTGAGGAACAAGTTTTCTTGT ACGTCATCCC
Loop on *E. coli* 16S rRNA (formed at staples 16-20)	
ETO20	CCGCCTTCGC CACCGGTATT CCTCCAGATC TCTACGCATT CACTCGATCCATCCAG
ETP15	GCGGGCCCCC GTCAATTCAT TTGAGTTTTA ACCTTGCGGC CGTACTCC CCAGGCGG
EP17	GCGT TAGCTCCGGA AGCCACGCCT CAAGGGCACA ACCTCCAAGT CGACATCGTT
EH16	CCAT CCAGGCGG TCGACTTAAC T CTGGATGGATCGAGTG
Loop on *S.* Typhi 16S rRNA (formed at staples 16-20)	
ETO20	CCGCCTTCGC CACCGGTATT CCTCCAGATC TCTACGCATT CACTCGATCCATCCAG
ETP15	GCGGGCCCCC GTCAATTCAT TTGAGTTTTA ACCTTGCGGC CGTACTCC CCAGGCGG
TP17	GCGT TAGCTCCGGA AGCCACGCCT CAAGGGCACA ACCTCCAAGT AGACATCGTT
TH16	CCAT CCAGGCGG TCTACTTAAC T CTGGATGGATCGAGTG

Supplementary Table 7. *Salmonella* 16S rRNA oligo pool used for the quantification of *S.* Typhi and *S.* Enteritidis. Dumbbell oligos for the two reference structures are included in the oligo pool at staples 18-23 and 29-34, respectively.

Name	Sequence
TE_1	GGTAAGGAGG TGATCCAACC GCAGGTTCCC CTACGGTTAC
TE_2	CTTGTTACGA CTTCACCCCA GTCATGAATC ACAAAGTGGT
TE_3	AAGCGCCCTC CCGAAGGTTA AGCTACCTAC TTCTTTTGCA
TE_4	ACCCACTCCC ATGGTGTGAC GGGCGGTGTG TACAAGGCCC
TE_5	GGGAACGTAT TCACCGTGGC ATTCTGATCC ACGATTACTA
TE_6	GCGATTCCGA CTTCATGGAG TCGAGTTGCA GACTCCAATC
TE_7	CGGACTACGA CGCACTTTAT GAGGTCCGCT TGCTCTCGCG
TE_8	AGGTCGCTTC TCTTTGTATG CGCCATTGTA GCACGTGTGT
TE_9	AGCCCTGGTC GTAAGGGCCA TGATGACTTG ACGTCATCCC
TE_10	CACCTTCCTC CAGTTTATCA CTGGCAGTCT CCTTTGAGTT
TE_11	CCCGGCCGGA CCGCTGGCAA CAAAGGATAA GGGTTGCGCT CGTTGCGGGA CTTAACCCAA
TE_12	CATTTCACAA CACGAGCTGA CGACAGCCAT GCAGCACCTG TCTCACAGTT CCCGAAGGCA CCAATCCATC
TE_13	TCTGTGGATG TCAAGACCAG GTAAGGTTCT TCGCGTTGCA TCGAATTAAA CCACATGCTC CACCGCTTGT
TE_14	GCGGGCCCCC GTCAATTCAT TTGAGTTTTA ACCTTGCGGC
TE_15	CGTACTCCCC AGGCGGTCTA CTTAACGCGT TAGCTCCGGA
TE_16	AGCCACGCCT CAAGGGCACA ACCTCCAAGT AGACATCGTT
TE_17	TACGGCGTGG ACTACCAGGG TATCTAATCC TGTTTGCTCC CCACGCTTTC GCACCTGAGC
TE_18	GTCAGTCTTT TCCTCTTTTGAGGAACAAGTTTTCTTGT GTCCAGGGGG
TE_19	CCGCCTTCGC TCCTCTTTTGAGGAACAAGTTTTCTTGT CACCGGTATT
TE_20	CCTCCAGATC TCCTCTTTTGAGGAACAAGTTTTCTTGT TCTACGCATT
TE_21	TCACCGCTAC TCCTCTTTTGAGGAACAAGTTTTCTTGT ACCTGGAATT
TE_22	CTACCCCCCT TCCTCTTTTGAGGAACAAGTTTTCTTGT CTACAAGACT
TE_23	CAAGCCTGCC TCCTCTTTTGAGGAACAAGTTTTCTTGT AGTTTCGAAT
TE_24	GCAGTTCCCA GGTTGAGCCC GGGGATTTCA CATCCGACTT
TE_25	GACAGACCGC CTGCGTGCGC TTTACGCCCA GTAATTCCGA TTAACGCTTG
TE_26	CACCCTCCGT ATTACCGCGG CTGCTGGCAC GGAGTTAGCC GGTGCTTCTT
TE_27	CTGCGGGTAA CGTCAATTGC TGCGGTTATT AACCACAACA CCTTCCTCCC CGCTGAAAGT
TE_28	ACTTTACAAC CCGAAGGCCT TCTTCATACA CGCGGCATGG
TE_29	CTGCATCAGG TCCTCTTTTGAGGAACAAGTTTTCTTGT CTTGCGCCCA
TE_30	TTGTGCAATA TCCTCTTTTGAGGAACAAGTTTTCTTGT TTCCCCACTG
TE_31	CTGCCTCCCG TCCTCTTTTGAGGAACAAGTTTTCTTGT TAGGAGTCTG
TE_32	GACCGTGTCT TCCTCTTTTGAGGAACAAGTTTTCTTGT CAGTTCCAGT
TE_33	GTGGCTGGTC TCCTCTTTTGAGGAACAAGTTTTCTTGT ATCCTCTCAG
TE_34	ACCAGCTAGG TCCTCTTTTGAGGAACAAGTTTTCTTGT GATCGTCGCC
TE_35	TTGGTGAGCC GTTACCTCAC CAACAAGCTA ATCCCATCTG GGCACATCTG
TE_36	ATGGCAAGAG GCCCGAAGGT CCCCCTCTTT GGTCTTGCGA
TE_37	CGTTATGCGG TATTAGCCAC CGTTTCCAGT AGTTATCCCC

TE_38	CTCCATCAGG CAGTTTCCCA GACATTACTC ACCCGTCCGC
TE_39	CACTCGTCAG CAAAGCAGCA AGCTGCTTCC TGTTACCGTT CGACTTGCAT
TE_40	GTGTTAGGCC TGCCGCCAGC GTTCAATCTG AGCCATGATC AAACTCT

Supplementary Table 8. Oligos used for the quantification of *S.* Typhi and *S.* Enteritidis. Loop structures are formed at staples 10-13 on both carriers.

Name	Sequence
TEO10	TGGACATGCTTGTGTG CACCTTCCTC CAGTTTATCA CTGGCAGTCT CCTTTGAGTT
TH13	CACACAAGCATGTCCA T TCTGGAAAGT TCTGTGGA CATG
EH13	CACACAAGCATGTCCA T TCTGGATTCT TCTGTGGA CATG

Supplementary Table 9. ***E. coli*** **16S rRNA oligo pool used for the detection of m^5C on 16S rRNA.**

Name	Sequence
EC_1	GGTAAGGAGG TGATCCAACC GCAGGTTCCC CTACGGTTAC CT
EC_2	TGTTACGACT TCACCCCAGT CATGAATCAC AAAGTGGTAA
EC_3	GCGCCCTCCC GAAGGTTAAG CTACCTACTT CTTTTGCAAC CCACTCCCATGG
EC_4	GGTGTGTACA AGGCCCGGGA ACGTATTCAC CGTGGCATTC TGATCCACGAT
EC_5	TACTAGCGAT TCCGACTTCA TGGAGTCGAG TTGCAGACTC
EC_6	CAATCCGGAC TACGACGCAC TTTATGAGGT CCGCTTGCTC
EC_7	TGTGTAGCCC TGGTCGTAAG GGCCATGATG ACTTGACGTC
EC_8	ATCCCCACCT TCCTCCAGTT TATCACTGGC AGTCTCCTTT
EC_9	GAGTTCCCGG CCGGACCGCT GGCAACAAAG GATAAGGGTT
EC_10	GCGCTCGTTG CGGGACTTAA CCCAACATTT CACAACACGA
EC_11	GCTGACGACA GCCATGCAGC ACCTGTCTCA CGGTTCCCGA
EC_12	AGGCACATTC TCATCTCTGA AAACTTCCGT GGATGTCAAG
EC_13	ACCAGGTAAG GTTCTTCGCG TTGCATCGAA TTAAACCACA
EC_14	TGCTCCACCG CTTGTGCGGG CCCCCGTCAA TTCATTTGAG
EC_15	TTTTAACCTT GCGGCCGTAC TCCCCAGGCG GTCGACTTAA
EC_16	CGCGTTAGCT CCGGAAGCCA CGCCTCAAGG GCACAACCTC
EC_17	CAAGTCGACA TCGTTTACGG CGTGGACTAC CAGGGTATCT
EC_18	AATCCTGTTT GCTCCCCACG CTTTCGCACC TGAGCGTCAG
EC_19	TCTTCGTCCA GGGGGCCGCC TTCGCCACCG GTATTCCTCC
EC_20	AGATCTCTAC GCATTTCACC GCTACACCTG GAATTCTACC
EC_21	CCCCTCTACG AGACTCAAGC TTGCCAGTAT CAGATGCAGT
EC_22	TCCCAGGTTG AGCCCGGGGA TTTCACATCT GACTTAACAA
EC_23	ACCGCCTGCG TGCGCTTTAC GCCCAGTAAT TCCGATTAAC
EC_24	GCTTGCACCC TCCGTATTAC CGCGGCTGCT GGCACGGAGT
EC_25	TAGCCGGTGC TTCTTCTGCG GGTAACGTCA ATGAGCAAA
EC_26	GGTATTAACT TTACTCCCTT CCTCCCCGCT GAAAGTACTT
EC_27	TACAACCCGA AGGCCTTCTT CATACACGCG GCATGGCTGC
EC_28	ATCAGGCTTG TCCTCTTTTGAGGAACAAGTTTTCTTGT CGCCCATTGT
EC_29	GCAATATTCC TCCTCTTTTGAGGAACAAGTTTTCTTGT CCACTGCTGC
EC_30	CTCCCGTAGG TCCTCTTTTGAGGAACAAGTTTTCTTGT AGTCTGGACC
EC_31	GTGTCTCAGT TCCTCTTTTGAGGAACAAGTTTTCTTGT TCCAGTGTGG
EC_32	CTGGTCATCC TCCTCTTTTGAGGAACAAGTTTTCTTGT TCTCAGACCA
EC_33	GCTAGGGATC TCCTCTTTTGAGGAACAAGTTTTCTTGT GTCGCCTTGG
EC_34	TGAGCCGTTA CCCCACCAAC AAGCTAATCC CATCTGGGCA
EC_35	CATCCGATGG CAAGAGGCCC GAAGGTCCCC CTCTTTGGTC
EC_36	TTGCGACGTT ATGCGGTATT AGCTACCGTT TCCAGTAGTT
EC_37	ATCCCCCTCC ATCAGGCAGT TTCCCAGACA TTACTCACCC
EC_38	GTCCGCCACT CGTCAGCAAA GAAGCAAGCT TCTTCCTGTT ACCGTTCGAC
EC_39	TTGCATGTGT TAGGCCTGCC GCCAGCGTTC AATCTGAGCC ATGATCAAAC TCTTCAATTT

Supplementary Table 10. ***A. baumannii*** **16S rRNA oligo pool used for the detection of m^5C on 16S rRNA.**

Name	Sequence
AC_1	TAAGGAGGTG ATCCAGCCGC AGGTTCCCCT ACGGCTACCT
AC_2	TGTTACGACT TCACCCCAGT CATCGGCCAC ACCGTGGTAA
AC_3	CCGCCCTCTT TGCAGTTAGG CTAGCTACTT CTGGTGCAAC AAACTCCCATGG
AC_4	GGTGTGTACA AGGCCCGGGA ACGTATTCAC CGCGGCATTC TGATCCGCGAT
AC_5	TACTAGCGAT TCCGACTTCA TGGAGTCGAG TTGCAGACTC
AC_6	CAATCCGGAC TACGATCGGC TTTTTGAGAT TAGCATCACA
AC_7	TGTGTAGCCC TGGCCGTAAG GGCCATGATG ACTTGACGTC
AC_8	GTCCCCGCCT TCCTCCAGTT TGTCACTGGC AGTATCCTTA
AC_9	AAGTTCCCAT CCGAAATGCT GGCAAGTAAG GAAAAGGGTT
AC_10	GCGCTCGTTG CGGGACTTAA CCCAACATCT CACGACACGA
AC_11	GCTGACGACA GCCATGCAGC ACCTGTATCT AGATTCCCGA
AC_12	AGGCACCAAT CCATCTCTGG AAAGTTTCTA GTATGTCAAG
AC_13	GCCAGGTAAG GTTCTTCGCG TTGCATCGAA TTAAACCACA
AC_14	TGCTCCACCG CTTGTGCGGG CCCCCGTCAA TTCATTTGAG
AC_15	TTTTAGTCTT GCGACCGTAC TCCCCAGGCG GTCTACTTAT
AC_16	CGCGTTAGCT GCGCCACTAA AGCCTCAAAG GCCCCAACGG
AC_17	CTAGTAGACA TCGTTTACGG CATGGACTAC CAGGGTATCT
AC_18	AATCCTGTTT GCTCCCCATG CTTTCGTACC TCAGCGTCAG
AC_19	TATTAGGCCA GATGGCTGCC TTCGCCATCG GTATTCCTCC
AC_20	AGATCTCTAC GCATTTCACC GCTACACCTG GAATTCTACC
AC_21	ATCCTCTCCC ATACTCTAGC TCACCAGTAT CGAATGCAAT
AC_22	TCCCAAGTTA AGCTCGGGGA TTTCACATCC GACTTAATAA
AC_23	GCCGCCTACG CACGCTTTAC GCCCAGTAAA TCCGATTAAC
AC_24	GCTCGCACCC TCTGTATTAC CGCGGCTGCT GGCACAGAGT
AC_25	TAGCCGGTGC TTATTCTGCG AGTAACGTCC ACTATCCCTA
AC_26	GGTATTAACT AGAGTAGCCT CCTCCTCGCT TAAAGTGCTT
AC_27	TACAACCATA AGGCCTTCTT CACACACGCG GCATGGCTGG
AC_28	ATCAGGGTTC TCCTCTTTTGAGGAACAAGTTTTCTTGT CCCCCATTGT
AC_29	CCAATATTCC TCCTCTTTTGAGGAACAAGTTTTCTTGT CCACTGCTGC
AC_30	CTCCCGTAGG TCCTCTTTTGAGGAACAAGTTTTCTTGT AGTCTGGGCC
AC_31	GTGTCTCAGT TCCTCTTTTGAGGAACAAGTTTTCTTGT CCCAGTGTGG
AC_32	CGGATCATCC TCCTCTTTTGAGGAACAAGTTTTCTTGT TCTCAGACCC
AC_33	GCTACAGATC TCCTCTTTTGAGGAACAAGTTTTCTTGT GTCGCCTTGG
AC_34	TAGGCCTTTA CCCCACCAAC TAGCTAATCC GACTTAGGCT
AC_35	CATCTATTAG CGCAAGGTCC GAAGATCCCC TGCTTTCTCC
AC_36	CGTAGGACGT ATGCGGTATT AGCATCCCTT TCGAGATGTT
AC_37	GTCCCCCACT AATAGGCAGA TTCCTAAGCA TTACTCACCC
AC_38	GTCCGCCGCT AGGTCCAGTA GCAAGCTACC TTCCCCCGCT CGACTTGCAT
AC_39	GTGTTAAGCC TGCCGCCAGC GTTCAATCTG AGCCATGATC AAACTCTTCA GTTA

Supplementary Table 11. Oligos used for the detection of m^{5}C in o6S rRNA of *E. coli* and *A. baumannii*. Loop structures are formed at staples 3-7 on both carriers.

Name	Sequence
EO7	TCGCGAGGTC GCTTCTCTTT GTATGCGCCA TTGTAGCACG CACTCGATCCATTCCC
AO7	TCGCTGTGTA GCAACCCTTT GTACCGACCA TTGTAGCACG CACTCGATCCATTCCC
EAH3	CCAT TCCCATGG TGTGACGGGC T GGGAATGGATCGAGTG
RT-PCR primers	
ERH1	TTTATGAGGT CCGCTTGCTC TCGCGAGGTC GCTTCTC
ERH2	TAAGGAGGTG ATCCAACCGC AGGTTCCCCT ACGGTTA
EPO1	GTGGATCAGA ATGCCACGGT
EPO2	CCTTGTTACG ACTTCACCCC A
EPB1	GTGTGTTGTA GTTTGGATTG GAGT
EPB2	ACACCCTCCC AAAAATTAAA CTACC
ARH1	TTTTGAGATT AGCATCACAT CGCTGTGTAG CAACCCT
ARH2	CCTACGGCTA CCTTGTTACG ACTTCACCCC AGTCATC
APO1	TCCATGAAGT CGGAATCGCT
APO2	ACCGCCCTCT TTGCAGTTAG
APB1	AGTTGATTGT AGTTTGGATT GGAGT
APB2	AACCACACCA TAATAACCAC CCT

Supplementary Notes

1. Nanopore Sensing Principle

To illustrate how our carrier designs result in multi-level drops in the nanopore current trace, it is necessary to provide an explanation of the principle of single-molecule nanopore sensing. The current response of a nanopore sensor, usually called a resistive pulse, is induced when a translocation event temporarily blocks the pathway of ions through the nanopore, thereby changing the nanopore resistance. The side containing analyte molecules is defined as the *cis* side of the nanopore, while the side at which the analytes finally arrive after translocation is the *trans* side. We simplify the local analyte to a nanosphere with a diameter of d, and x is the distance of its center from the *cis* opening of the nanopore, as shown in Supplementary Fig. 8. When an applied voltage drives the carrier backbone into the sensing area, d is defined by the diameter of the characteristic double-helix structure of the RNA/DNA hybrid and remains basically the same. The nanopore current drops as x becomes smaller until the backbone occupies the entire sensing area and the current trace reaches the first-level platform. When the secondary nanostructure on the carrier reaches the sensing area, d significantly increases, resulting in a further decrease in the nanopore current. The current drops to its lowest point when the synergistic effect of d and x reaches its maximum. Then, as the carrier exits the sensing area, the nanopore resistance changes in reverse until the current trace returns to the original baseline. Based on this principle, the magnitude, duration, and frequency of the multi-level resistance pulses can reveal a wealth of useful information about the properties of the carrier and the secondary nanostructures on it.

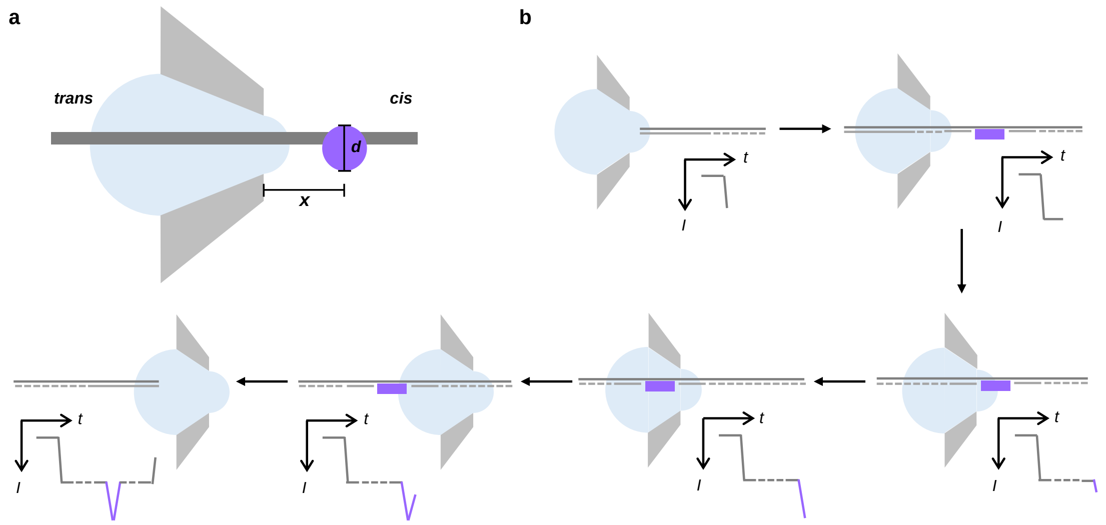

Supplementary Figure 8. Principle of multi-level nanopore current signal generation. a, Schematic illustration of a carrier translocating through the sensing area of the nanopore. **b**, Detailed process of how the current trace is affected as a carrier is driven through the nanopore. The entry of the bare carrier scaffold into the nanopore produces the first-level current drop. When the secondary structure starts to pass through the pore, the second-level current drop appears. The current trace reaches the minimum when the secondary structure arrives at the center of the sensing area and occupies the largest possible volume. As the secondary structure begins to flow out of the sensitive

tip region, the current returns to its original level.

An important thing to mention is that, the carrier strand's conformation during translocation through the nanopore is a crucial factor in successful nanopore readout. When captured at one end, the strand can pass through in either a linear, single-file manner or a folded conformation. In certain cases, RNA/DNA molecules can even become entangled and form knots. Supplementary Fig. 9 illustrates linear, folded, and knotted translocations of MS2 carriers featuring a six-dumbbell reference structure (green) and a latched loop at the target site (blue). The current traces clearly demonstrate that a linear conformation significantly enhances the ability to distinguish the spikes produced by each structure. In contrast, folds and knots can cause structures to be simultaneously positioned at similar locations within the pore. Although some spikes may still be discernible in these cases, inferring the RNA/DNA structures from the current signal becomes much more challenging. To minimize confounding factors, only linear events with clearly observable spikes were considered for analysis in this work. For further clarification, we define events as forward when the targeted side of the carrier passes through the nanopore first. Conversely, events are considered backward when the bare carrier backbone translocates through the pore before the barcode side.

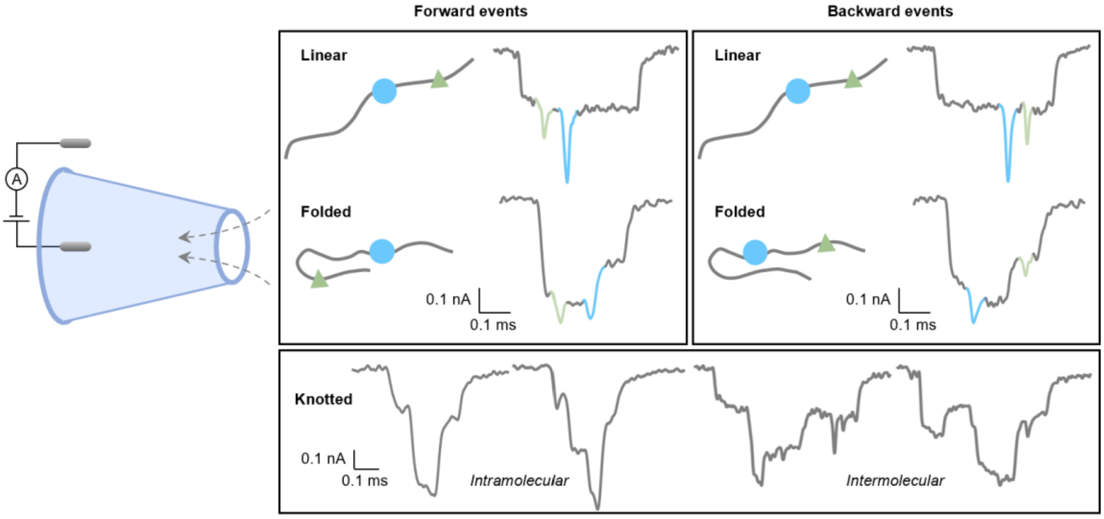

Supplementary Figure 9. Representative current traces of linear versus folded and knotted translocations of MS2 carrier through nanopores. The MS2 carrier design follows that shown in Fig. 2, featuring a reference structure (green) at one side of the carrier and a target site in the middle region for loop formation. We classify events as "forward" when the reference-containing side of the carrier enters the nanopore first, and "backward" when the unmodified carrier backbone translocates before the reference-containing terminus.

2. Modeling and Calculations

2.1 Two-state energy model

The experimental data presented in Fig. 3b-d provides strong support for the efficacy of the RNA-SCAN approach. However, to gain a deeper understanding and rationalize the observed effects, it is valuable to consider a theoretical model. We developed a simplified energetic model of the RNA-SCAN system. This model considers two distinct energy states corresponding to the loop formation: “latched” (l) and “unlatched” (u) (Fig. 3e). The energy difference between these two states can be expressed as:

$$\Delta G_0 = G_l - G_u$$

In a similar vein, we also identified “latched” (l_m) and “unlatched” (u_m) energy levels for modified sequences. The presence of modifications introduces an important variable into our energetic landscape. They can either strengthen or weaken the stability of the duplex between the nanolatch and the RNA scaffold, thereby shifting the “latched” energy level, while we assume that the “unlatched” state of the loop remains at the same energy level. This assumption is based on the premise that in the unlatched state, the modified nucleotides are not engaged in base-pairing interactions and thus do not significantly alter the system’s energy. Given these considerations, the energy difference for modified sequences can be expressed as:

$$\Delta G_1 = G_{l\_m} - G_{u\_m}$$

To quantify the effect of modifications on the system’s stability, we defined the change in free energy due to the modification as:

$$\Delta\Delta G_1 = \Delta G_1 - \Delta G_0$$

In this formulation, a negative $\Delta\Delta G_1$ value indicates stabilization of the system by the modification, while a positive value suggests destabilization. To relate these energy changes to observable quantities, we utilized the fundamental relationship between the equilibrium constant, K, and the standard free energy change, ΔG°:

$$K = \exp(\frac{-\Delta G^{\circ}}{RT})$$

where R is the universal gas constant (8.314 J/(mol·K)), and T is the absolute temperature in Kelvin. This equation allows us to predict how changes in free energy will affect the equilibrium of the system. Applying this principle to our modified system, we can express the equilibrium constant K_1 of the modified state as:

$$K_1 = K_0 \exp(\frac{-\Delta\Delta G_1^{\circ}}{RT})$$

Notably, our analysis in this study is grounded in the nearest-neighbor model[1-4], which posits that only adjacent base pairs significantly influence the free energy of duplex formation. This model provides a simplified assumption that allows us to treat the effects of multiple modifications as additive, given that our target sequences do not contain adjacent modified bases. Based on this principle, we could express the equilibrium constant K_2 for a system with two modifications as:

$$K_2 = K_0 \exp(\frac{-\Delta\Delta G_2^{\circ}}{RT})$$

Extending this concept, we could generalize the equation for n modifications:

$$K_n = K_0 \exp(\frac{-(n)\Delta\Delta G_{(n)}^{\circ}}{RT})$$

where n is the number of modifications and $\Delta\Delta G_{(n)}^{\circ}$ represents the average value for the stabilization or destabilization effect of the modifications. To facilitate analysis, we took the natural logarithm of both sides:

$$\ln(K_n) = \frac{-(n)\Delta\Delta G_{(n)}^{\circ}}{RT} + \ln(K_0)$$

This transformation allowed us to plot $\ln(K_n)$ against n for all types of nucleotide modifications studied in this work (Fig. 3f). The resulting linear relationships demonstrate good agreement between our experimental data and the theoretical model, validating our approach as a robust first approximation. The strong correlation observed in these plots not only supports the additivity assumption of our model but also provides a quantitative tool for predicting the effects of multiple modifications. This insight could prove valuable in designing RNA-SCAN systems with enhanced sensitivity or specificity for particular modification patterns.

2.2 The nearest-neighbor model

The nearest-neighbor (NN) model, originally developed by Crothers et al.[3] and Tinoco et al.[4], has become one of the most widely used methods for predicting the thermostability of Watson-Crick-Franklin nucleic acid duplexes. This model is predicated on the assumption of a two-state melting behavior for nucleic acid duplexes. According to this approach, the thermodynamic parameters (ΔH°, ΔS°, and ΔG°) for nucleic acid duplex formation can be decomposed into three primary components:

1. Helix Initiation: This represents the free energy change associated with the formation of the first base pair in the double helix. It accounts for the entropic cost of bringing two strands together and initiating the helical structure.

2. Helix Propagation: This is calculated as the sum of contributions from each subsequent base pair. The model posits that the stability of a given base pair is influenced primarily by its immediate neighbors, hence the term "nearest-neighbor".

3. Mixing Entropy: This term accounts for the additional entropy change specific to self-complementary strands. It reflects the increased number of ways in which self-complementary molecules can associate compared to non-self-complementary ones.

Since its inception, the NN model has undergone significant refinement and expansion. Numerous research groups have contributed to the development and validation of NN parameters for various types of nucleic acid duplexes, including dsDNA, dsRNA, and RNA/DNA hybrids. These parameters now enable accurate predictions of duplex stability across a wide range of sequences and conditions. However, it's important to note that while the NN model is highly effective for standard Watson-Crick-Franklin base pairs, its application to non-canonical structures or modified nucleotides often requires careful consideration and, in some cases, empirical corrections. Therefore, in the context of our study, the NN model provides a theoretical framework for understanding and predicting the effects of nucleotide modifications on duplex stability rather than offering accurate analysis values.

2.3 Calculation of nucleotide modifications

Given the limited data available on how modifications affect the stability of RNA/DNA duplexes, we turned our attention to investigating the effects of modifications on RNA bases within dsRNA duplexes and on DNA bases within dsDNA duplexes. Considering the established stability hierarchy of:

$$dsRNA > RNA/DNA > dsDNA$$

we would anticipate that our experimental changes in free energy ($\Delta\Delta G^{\circ}_{(n)}$) align with values reported in literature, albeit with some adjustments due to the hybrid nature of our system. And acknowledging that our experiments were conducted at 25°C, we recalibrated the literature thermodynamic data, which was obtained at 37°C, using the formula:

$$\Delta\Delta G^{\circ} = \Delta\Delta H^{\circ} - T\Delta\Delta S^{\circ}$$

where:

$$\Delta\Delta H^{\circ} = \Delta H^{\circ}_{1} - \Delta H^{\circ}_{0}$$
$$\Delta\Delta S^{\circ} = \Delta S^{\circ}_{1} - \Delta S^{\circ}_{0}$$

which assumes constant enthalpy (ΔH°) and entropy (ΔS°) across the temperature range of interest. Apart from the influence of temperature, we also found that certain modifications show significant sequence-dependent destabilization effects. For example, the conversion of adenosine (A) to inosine (I) has been reported to cause destabilization ranging from 1.32 to 3.85 kcal/mol depending on the surrounding sequence context[5]. In such instances, we opted for a more precise calculation of destabilization using the NN model, tailored to each specific modification, instead of relying on a general average.

To illustrate the application of the NN model in our analysis, consider the following example sequence:

5'	G	C	A	A	C	3'
3'	C	G	T	T	G	5'

For each base pair, except for the terminal base pairs, the NN model considers base stacking interactions with two adjacent base-pairs and thus ΔG°_{o} of the above duplex can be written as:

$$\Delta G^{\circ}_{0} = \Delta G^{\circ}_{initiation} + \Delta G^{\circ}\begin{pmatrix} G & C \\ C & G \end{pmatrix} + \Delta G^{\circ}\begin{pmatrix} C & A \\ G & T \end{pmatrix} + \Delta G^{\circ}\begin{pmatrix} A & A \\ T & T \end{pmatrix} + \Delta G^{\circ}\begin{pmatrix} A & C \\ T & G \end{pmatrix}$$

where $\Delta G^{\circ}_{initiation}$ is the energy penalty associated with duplex formation, which accounts for the entropic cost of bringing two strands together. And $\Delta G^{\circ}\begin{pmatrix} W & Y \\ X & Z \end{pmatrix}$ is known as the NN parameter for a particular base pair (W-X) adjacent to another base pair (Y-Z)[6]. This formulation allows us to break down the overall stability of the duplex into contributions from each nearest-neighbor interaction along the sequence. Similarly, when a different nucleotide or modification (take the I modification as example, highlighted in red) is introduced into the sequence:

5'	G	C	I	A	C	3'
3'	C	G	T	T	G	5'

we have:

$$\Delta G_1^\circ = \Delta G_{initiation}^\circ + \Delta G^\circ \begin{pmatrix} G & C \\ C & G \end{pmatrix} + \Delta G^\circ \begin{pmatrix} C & I \\ G & T \end{pmatrix} + \Delta G^\circ \begin{pmatrix} I & A \\ T & T \end{pmatrix} + \Delta G^\circ \begin{pmatrix} A & C \\ T & G \end{pmatrix}$$

And so, we get:

$$\Delta\Delta G_1^\circ = \Delta G_1^\circ - \Delta G_0^\circ = \Delta G^\circ \begin{pmatrix} C & I \\ G & T \end{pmatrix} + \Delta G^\circ \begin{pmatrix} I & A \\ T & T \end{pmatrix} - \Delta G^\circ \begin{pmatrix} C & A \\ G & T \end{pmatrix} - \Delta G^\circ \begin{pmatrix} A & A \\ T & T \end{pmatrix}$$

$\Delta\Delta G_1^\circ$ is assumed as constant and independence of salt concentration. And NN values are averaged over all the A to I modification sites to give $\Delta\Delta G_{(n)}^\circ$.The enthalpies and entropies of duplexes are predicted in a parallel way:

$$\Delta\Delta H_1^\circ = \Delta H_1^\circ - \Delta H_0^\circ = \Delta H^\circ \begin{pmatrix} C & I \\ G & T \end{pmatrix} + \Delta H^\circ \begin{pmatrix} I & A \\ T & T \end{pmatrix} - \Delta H^\circ \begin{pmatrix} C & A \\ G & T \end{pmatrix} - \Delta H^\circ \begin{pmatrix} A & A \\ T & T \end{pmatrix}$$

$$\Delta\Delta S_1^\circ = \Delta S_1^\circ - \Delta S_0^\circ = \Delta S^\circ \begin{pmatrix} C & I \\ G & T \end{pmatrix} + \Delta S^\circ \begin{pmatrix} I & A \\ T & T \end{pmatrix} - \Delta S^\circ \begin{pmatrix} C & A \\ G & T \end{pmatrix} - \Delta S^\circ \begin{pmatrix} A & A \\ T & T \end{pmatrix}$$

Literature values of $\Delta\Delta G_{(n)}^\circ$

Unfortunately, due to the absence of comprehensive thermodynamic data for MeC modifications in the literature, we were unable to include it in our quantitative comparison. This gap in our knowledge highlights the pressing need for further research into the thermodynamic properties of MeC-modified nucleic acids. Despite this limitation, we were able to proceed with our analysis using available data for other modifications. Specifically, we found reliable literature values for the thermodynamic changes associated with the conversion of C to 5mC/m^{5}C, and the conversion of A to I (Supplementary Table 12). These data allowed us to make meaningful comparisons and draw insights into the effects of these particular modifications on duplex stability.

Supplementary Table 12. Adjustment of $\Delta\Delta G^\circ$ to 25°C from reported values at 37°C. For 5mC in dsDNA[7], literature conditions are 100 mM KCl, 10 mM K_2HPO_4, 1 mM K_2EDTA, pH 7.0. For m5C in dsRNA[8], ΔH° and ΔS° are absent, and the $\Delta\Delta G^\circ$ is calculated ab initio using molecular dynamics/quantum mechanical simulations at 37°C. For inosine in dsDNA[9], the literature conditions are 1 M NaCl, 10 mM sodium cacodylate, 0.5 mM Na_2EDTA, pH 7.0. For inosine in dsRNA[5], literature conditions are 1 M NaCl, 20 mM sodium cacodylate, 0.5 mM Na_2EDTA, pH 7.0.

Modification	Type of Duplex	$\Delta\Delta H^\circ$(kcal/mol)	$\Delta\Delta S^\circ$(cal/mol)	$<\Delta\Delta G^\circ>$ (kcal/mol)
C -> 5mC	dsDNA[7]	-1.63	-4.63	-0.24
	dsRNA[8]	N/A	N/A	0.41
A -> I	dsDNA[9]	-3.13	-9.54	1.33
	dsRNA[5]	-4.34	-21.84	2.17

NN modelling of inosine modifications

For the inosine-modified nanolatches, we conducted a detailed analysis of the relevant NN pairs (Supplementary Table 13) and their corresponding thermodynamic data (Supplementary Tables 14 and 15). We notice that two of the four inosine bases in our nanolatch design shared identical surrounding sequences. That is to say, the modified sequence context of Number 1 appeared twice.

This repetition provides a valuable opportunity to assess the consistency and sequence-dependence of inosine's impact on duplex stability.

Supplementary Table 13. Relevant NN pairs for each sequence of interest on inosine-modified nanolatches.

Number	Sequence of Interest	Unmodified Sequence	1st NN Modified	2nd NN Modified	1st NN Unmodified	2nd NN Unmodified
1	$\begin{pmatrix} A & I & C \\ U & U & G \end{pmatrix}$	$\begin{pmatrix} A & A & C \\ U & U & G \end{pmatrix}$	$\begin{pmatrix} A & I \\ U & U \end{pmatrix}$	$\begin{pmatrix} I & C \\ U & G \end{pmatrix}$	$\begin{pmatrix} A & A \\ U & U \end{pmatrix}$	$\begin{pmatrix} A & C \\ U & G \end{pmatrix}$
2	$\begin{pmatrix} G & I & G \\ C & U & C \end{pmatrix}$	$\begin{pmatrix} G & A & G \\ C & U & C \end{pmatrix}$	$\begin{pmatrix} G & I \\ C & U \end{pmatrix}$	$\begin{pmatrix} I & G \\ U & C \end{pmatrix}$	$\begin{pmatrix} G & A \\ C & U \end{pmatrix}$	$\begin{pmatrix} A & G \\ U & C \end{pmatrix}$
3	$\begin{pmatrix} A & I & A \\ U & U & U \end{pmatrix}$	$\begin{pmatrix} A & A & A \\ U & U & U \end{pmatrix}$	$\begin{pmatrix} A & I \\ U & U \end{pmatrix}$	$\begin{pmatrix} I & A \\ U & U \end{pmatrix}$	$\begin{pmatrix} A & A \\ U & U \end{pmatrix}$	$\begin{pmatrix} A & A \\ U & U \end{pmatrix}$

Supplementary Table 14. Thermodynamic data of the NN pairs listed in Supplementary Table 13, measured on dsDNA in 1 M NaCl, 10 mM sodium cacodylate, 0.5 mM Na_2EDTA, pH 7.0 at 25°C[9].

Number	1st NN		2nd NN		$\Delta\Delta G_1^\circ$(kcal/mol)
	$\Delta\Delta H^\circ$(kcal/mol)	$\Delta\Delta S^\circ$(cal/mol)	$\Delta\Delta H^\circ$(kcal/mol)	$\Delta\Delta S^\circ$(cal/mol)	
1	0.49	-0.70	-4.30	-12.10	0.01
2	-3.50	-10.60	0.10	-1.00	0.06
3	0.49	-0.70	-0.80	-4.30	1.18

Supplementary Table 15. Thermodynamic data of the NN pairs listed in Supplementary Table 13, measured on dsRNA in 1 M NaCl, 20 mM sodium cacodylate, 0.5 mM Na_2EDTA, pH 7.0 at 25°C[5]. Note that the values of ΔH° and $\Delta\Delta G_1^\circ$ are in kcal/mol and the values of ΔS° are in cal/mol.

Number	1st NN Modified		2nd NN Modified		1st NN Unmodified		2nd NN Unmodified		$\Delta\Delta G_1^\circ$
	ΔH°	ΔS°	ΔH°	ΔS°	ΔH°	ΔS°	ΔH°	ΔS°	
1	11.68	36.30	11.56	-34.00	-6.82	-19.00	-11.40	-29.5	1.48
2	9.81	27.40	13.38	39.30	-12.44	-32.50	-10.48	-27.1	1.85
3	11.68	36.30	8.22	27.90	-6.82	-19.00	-6.82	-19.00	1.55

From the calculations detailed in the tables, we derived two key values of $\Delta\Delta G_{(n)}^\circ$ in this study, which were calculated by averaging the NN values over the four A to I modification sites:

$$\Delta\Delta G_{(n)}^\circ = \left(2 \times \Delta\Delta G_1^\circ(\#1) + \Delta\Delta G_1^\circ(\#2) + \Delta\Delta G_1^\circ(\#3)\right) \div 4$$

For A to I conversions in dsDNA, $\Delta\Delta G_{(n)}^\circ = 0.31$ kcal/mol; for A to I conversions in dsRNA, $\Delta\Delta G_{(n)}^\circ = 1.59$ kcal/mol.

Comparison of literature, NN model, and measured $\Delta\Delta G^{\circ}_{(n)}$ values

Having summarized thermodynamic data from literature and the NN model as above, we further collected gradients from linear fitting results of $\ln(K_n)$ against *n* plots in Fig. 3f, and calculated $\Delta\Delta G^{\circ}_{(n)}$ from:

$$\ln(K_n) = \frac{-(n)\Delta\Delta G^{\circ}_{(n)}}{RT} + \ln(K_0)$$

A comparison between $\Delta\Delta G^{\circ}_{(n)}$ values is shown in Supplementary Table 16.

Supplementary Table 16. Comparison of literature, NN model, and measured $\Delta\Delta G^{\circ}_{(n)}$ values of modified nanolatches. NN values are calculated at 25°C for each modification. $< \Delta\Delta G^{\circ} >$ values are also adjusted to 25°C to keep consistency with our experimental conditions.

Modification	Type of duplex	Literature $< \Delta\Delta G^{\circ} >$ (kcal/mol)	NN $\Delta\Delta G^{\circ}_{(n)}$ (kcal/mol)	Gradient from plots in Fig. 3f	Measured $\Delta\Delta G^{\circ}_{(n)}$ (kcal/mol)
C to 5mC/m^{5}C	dsDNA	-0.24 [7]	N/A	0.22	-0.13 ± 0.003
	dsRNA	0.41 [8]	N/A		
A to I	dsDNA	1.33 [6,9]	0.31	-1.35	0.80 ± 0.10
	dsRNA	2.17 [5]	1.59		

Our experimental measurements of the free energy change $\Delta\Delta G^{\circ}_{(n)}$ for 5-methylcytosine fall within the expected range based on literature values for both dsRNA and dsDNA duplexes. This consistency not only validates our experimental approach but also reinforces the reliability of our results. In contrast, the measured $\Delta\Delta G^{\circ}_{(n)}$ value for inosine modifications presents an intriguing discrepancy. While it aligns with the range predicted by NN models, it diverges from previously reported literature values. It suggests that the local sequence context plays a more significant role in determining the energetic impact of inosine than previously recognized, highlighting the need for more nuanced models of nucleotide modifications.

Notably, our analysis of 5-methylcytosine reveals a particularly interesting phenomenon: the average $\Delta\Delta G^{\circ}_{(n)}$ values indicate that this modification could either stabilize or destabilize the molecular structure, depending on its conformational state. Specifically, the stabilizing or destabilizing effect appears to be contingent on whether the molecule more closely resembles the B-form structure typical of dsDNA or the A-form structure characteristic of dsRNA. This observation highlights the complex interplay between chemical modifications and nucleic acid structure, and emphasizes the need for careful consideration of structural context when predicting the impact of modifications on duplex stability.

These findings contribute to our understanding of how chemical modifications influence the thermodynamic properties of nucleic acid duplexes and underscore the importance of considering both sequence context and structural conformation in predicting and interpreting the effects of such modifications.

2.4 Extension of NN model to nucleotide mutations

Expanding upon our analysis of nucleotide modifications, we further extended the NN model to encompass nucleotide mutations. To validate the applicability of the NN model in this context, we calculated the $\Delta\Delta G^{\circ}_{(n)}$ values for each type of nucleotide mutations present in our MHm1-MHm4 nanolatches and compared them to established NN literature values. The relevant NN pairs for each modification site and their corresponding calculated $\Delta\Delta G^{\circ}_{1}$ values are presented in Supplementary Tables 17 and 18.

Supplementary Table 17. Relevant NN pairs for each sequence of interest on nanolatches with mismatches. Nanolatches MHm1-MHm3 have mismatches Number 1-3 respectively, while MHm4 has both Number 1 and 2 mismatches.

Number	Sequence of Interest	Unmodified Sequence	1st NN Modified	2nd NN Modified	1st NN Unmodified	2nd NN Unmodified
1	$\begin{pmatrix} A & A & G \\ U & G & C \end{pmatrix}$	$\begin{pmatrix} A & C & G \\ U & G & C \end{pmatrix}$	$\begin{pmatrix} A & A \\ U & G \end{pmatrix}$	$\begin{pmatrix} A & G \\ G & C \end{pmatrix}$	$\begin{pmatrix} A & C \\ U & G \end{pmatrix}$	$\begin{pmatrix} C & G \\ G & C \end{pmatrix}$
2	$\begin{pmatrix} A & A & T \\ U & G & A \end{pmatrix}$	$\begin{pmatrix} A & C & T \\ U & G & A \end{pmatrix}$	$\begin{pmatrix} A & A \\ U & G \end{pmatrix}$	$\begin{pmatrix} A & T \\ G & A \end{pmatrix}$	$\begin{pmatrix} A & C \\ U & G \end{pmatrix}$	$\begin{pmatrix} C & T \\ G & A \end{pmatrix}$
3	$\begin{pmatrix} A & T & T \\ U & C & A \end{pmatrix}$	$\begin{pmatrix} A & G & T \\ U & C & A \end{pmatrix}$	$\begin{pmatrix} A & T \\ U & C \end{pmatrix}$	$\begin{pmatrix} T & T \\ C & A \end{pmatrix}$	$\begin{pmatrix} A & G \\ U & C \end{pmatrix}$	$\begin{pmatrix} G & T \\ C & A \end{pmatrix}$

Supplementary Table 18. Thermodynamic data of the NN pairs listed in Supplementary Table 17, measured on RNA/DNA hybrid duplexes in 1 M NaCl, 10 mM Na_2HPO_4, 0.5 mM Na_2EDTA, pH 7.0 at 25°C[10]. Note that the values of ΔH° and $\Delta\Delta G^{\circ}_{1}$ are in kcal/mol and the values of ΔS° are in cal/mol.

Number	1st NN Modified		2nd NN Modified		1st NN Unmodified		2nd NN Unmodified		$\Delta\Delta G^{\circ}_{1}$
	ΔH°	ΔS°	ΔH°	ΔS°	ΔH°	ΔS°	ΔH°	ΔS°	
1	-21.91	-75.00	-2.47	-7.20	-5.33	-12.70	-14.06	-37.20	4.64
2	-21.91	-75.00	-10.21	-34.80	-5.33	-12.70	-10.78	-29.30	4.20
3	2.57	5.00	-1.90	-9.20	-9.29	-26.10	-7.15	-18.70	5.01

Given that we had only two data points per nanolatch, a linear fitting approach was not feasible. However, we were able to extract a measured $\Delta\Delta G^{\circ}$ for each mismatch using the following equation:

$$\Delta\Delta G^{\circ} = -RT \ln(\frac{K_1}{K_0})$$

The error (δ) is then calculated via error propagation:

$$\delta(\Delta\Delta G^{\circ}) = -RT \sqrt{\left(\frac{\delta K_1}{K_1}\right)^2 + \left(\frac{\delta K_0}{K_0}\right)^2}$$

And as the equilibrium constant is related to the number of positive events by:

$$K = \frac{p}{100 - p}$$

where p denotes the number of positive events in the first 100 linear events for each nanopore measurement, the error in K can be expressed as:

$$\delta K = \frac{100 * \delta p}{(p - 100)^2}$$

where δp is the standard error of the mean for the positive event measurements. We summarize the results below.

Supplementary Table 19. Comparison of NN model and measured $\Delta\Delta G^{\circ}_{(n)}$ values of mutant nanolatches. For MHm4, it is assumed that its destabilization effects from two mismatches is additive.

Name of Nanolatch	NN $\Delta\Delta G^{\circ}_{(n)}$ (kcal/mol)	Measured $\Delta\Delta G^{\circ}_{(n)}$ (kcal/mol)
MHm1	4.64	1.53 ± 0.12
MHm2	4.20	1.79 ± 0.16
MHm3	5.01	0.84 ± 0.06
MHm4	8.84	2.45 ± 0.30

Here, the measured $\Delta\Delta G^{\circ}_{(n)}$ values appear to deviate from the NN values by a factor of 2-3, suggesting that the mismatches are less destabilizing than anticipated. This discrepancy can be attributed to the fact that NN parameters are derived from melting experiments involving short, double-stranded oligos of approximately 10 bp in length, which differ significantly from the complex nanolatch structure under investigation. Another factor contributing to the discrepancy may be the omission of salt effects, given the substantial difference between the buffer conditions used in our experiments (0.25 mM $MgCl_2$ and 0.5X TE) and those employed in the literature (1 M NaCl). Tan and Chen[11] have demonstrated that salt concentration can significantly influence the stability of duplexes. In addition to the factors mentioned above, the observed discrepancies can be explained by considering the location of the mismatches in MHm1, MHm2, and MHm4. These mismatches are situated near the junction where the nanolatch binds to the overhang, close to the end of the helix formed by the nanolatch and the RNA scaffold. Mismatches in this region are more stable by approximately 0.5 kcal/mol, potentially due to the increased flexibility towards the helix terminus[12], which allows for better accommodation of non-Watson-Crick-Franklin base pairing. Moreover, in the case of MHm4, literature indicates that two closely spaced mismatches have less than additive destabilizing effects[12], accounting for the 1.0 kcal/mol difference compared to the sum of the individual destabilizing effects of MHm1 and MHm2. In contrast, the mismatch in MHm3 is in the competitive region, which does not penalize the initial binding of the nanolatch to the complementary region.

2.5 Thermodynamic analysis of nucleotide mutations using NUPACK

When examining nucleotide mutations, we have alternative options beyond the NN model for analyzing their influence on the thermodynamics of RNA-nanolatch interactions. NUPACK[13], a growing software suite for the analysis and design of nucleic acid structures, has been widely adopted in the emerging fields of molecular programming and nucleic acid nanotechnology. We selected it as a complementary analysis tool to our NN model calculations for nucleotide mutations in MHm1-MHm6.

For our simulations, we used sequences from the target site on MS2 RNA and the corresponding parts of nanolatches. The temperature was set at 25°C to match our room-temperature nanopore measurements. Strand concentrations were set at 0.3 nM, consistent with the carrier concentration during the final-step incubation and nanopore measurements. As NUPACK cannot simulate RNA/DNA hybrids, we examined both dsRNA and dsDNA scenarios for each sequence, as shown in Supplementary Table 20. The thermodynamic properties of RNA/DNA hybrids are expected to fall between those of dsRNA and dsDNA.

Supplementary Table 20 reveals that all nanolatches with nucleotide mutations exhibit a smaller absolute value of free energy than the original MH sequence when forming duplexes with the MS2 RNA target sequence. While we present values for MHm3, we exclude it from comparing with other mutations as its nucleotide substitution occurs in the competitive area unlike other mutations that occur in the pairing area. In our competitive reaction design, nucleotide variations in the competitive area have reduced influence on RNA scaffold-nanolatch interactions because such mutations only affect the competition between the nanolatch and the complementary oligo, while preserving the critical initial base pairing between the nanolatch and RNA scaffold that is fundamental for loop formation. Therefore, comparing MHm3 with mutations in the pairing area based solely on thermodynamic values would not provide meaningful insights into their relative effects on detection efficiency.

Among the other nanolatches, NUPACK calculations reveal that MHm5 exhibits a slightly higher average absolute free energy value when considering both dsRNA and dsDNA formations, suggesting stronger overall potential for loop latching with the RNA scaffold. This computational prediction aligns well with our experimental observations in Fig. 2c, where MHm5 shows the highest positive event ratio among all mismatched nanolatches (excluding MHm3) across three independent experiments. MHm1, MHm2, and MHm6 demonstrate comparable free energy values for their base-pairing interactions with the target RNA sequence, which corresponds to their similar positive event ratios in nanopore measurements. As predicted by thermodynamic principles, MHm4, with its two-nucleotide mismatch, shows the smallest absolute free energy value, indicating the least stable loop formation with MS2 RNA - a prediction confirmed by its consistently low positive event ratio in Fig. 2c. The NUPACK-calculated thermodynamic values align well with our nanopore measurement results for different mutated nanolatches.

Supplementary Table 20. Thermodynamic simulations of interactions between the target MS2 RNA sequence and nanolatches MH and MHm1-MHm6 using NUPACK. Nucleotide variations are marked in red. Minimum free energy (MFE) structures are directly adapted from NUPACK outputs. Structural formations are examined in both dsRNA and dsDNA scenarios to approximate RNA/DNA hybrid behavior.

Nanolatch Name	Base pairing sequence at target site	NUPACK simulation MFE structure	Free energy (kcal/mol)
MH	RNA:UCUUGCUUGACUCAUUUC<br>Nanolatch: AGAACGAACTGAGTAAAG	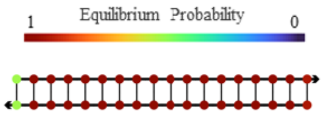	dsRNA: -34.66<br>dsDNA: -26.75
MHm1	RNA: UCUUGCUUGACUCAUUUC<br>Nanolatch: AGAA**A**GAACTGAGTAAAG	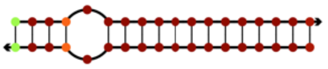	dsRNA: -28.44<br>dsDNA: -22.49
MHm2	RNA: UCUUGCUUGACUCAUUUC<br>Nanolatch: AGAACGAA**A**TGAGTAAAG	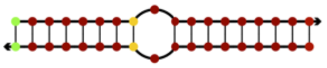	dsRNA: -27.87<br>dsDNA: -23.41
MHm3	RNA: UCUUGCUUGACUCAUUUC<br>Nanolatch: AGAACGAACTGA**T**TAAAG	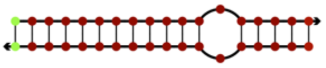	dsRNA: -27.87<br>dsDNA: -22.21
MHm4	RNA: UCUUGCUUGACUCAUUUC<br>Nanolatch: AGAA**A**GAA**A**TGAGTAAAG	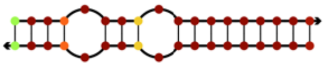	dsRNA: -21.66<br>dsDNA: -19.14
MHm5	RNA: UCUUGCUU GACUCAUUUC<br>Nanolatch: AGAACGAA**A**CTGAGTAAAG	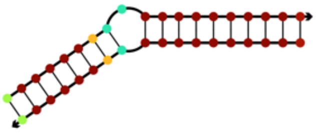	dsRNA: -30.59<br>dsDNA: -22.91
MHm6	RNA: UCUUGCUUGACUCAUUUC<br>Nanolatch: AGAACGAA**--**TGAGTAAAG	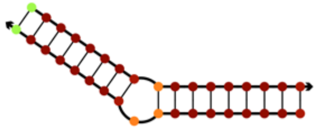	dsRNA: -27.03<br>dsDNA: -20.79

3. Nanopore Measurement Details

3.1 Verification of the RNA-SCAN design.

The green spikes refer to the reference structure while the blue spikes suggest latched loops.

Example "loop unlatched" events

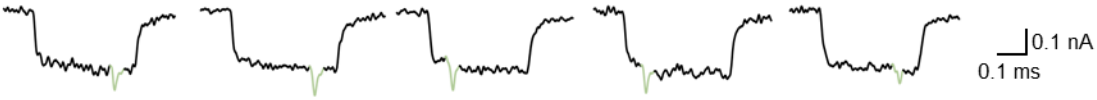

Example "loop latched" events

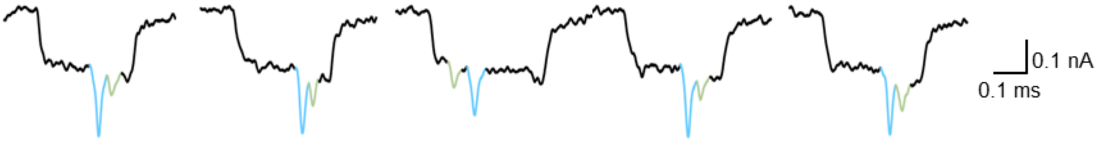

3.2 Dependence of positive event ratio on the concentration of nanolatch.

Example positive events

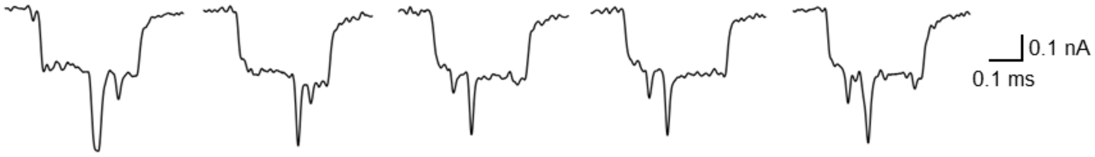

Example negative events

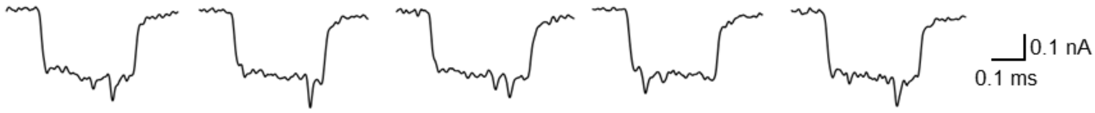

(1) c(MH nanolatch):c(MS2 carrier) = 1:1

a. Positive Event Ratio = 8%; $d(\text{nm}) \approx I_{600mV}(\text{nA}) = 10.7$

b. Positive Event Ratio = 7%; $d(\text{nm}) \approx I_{600mV}(\text{nA}) = 9.7$

c. Positive Event Ratio = 10%; $d(\text{nm}) \approx I_{600mV}(\text{nA}) = 10.2$

(2) c(MH nanolatch):c(MS2 carrier) = 5:1

a. Positive Event Ratio = 19%; $d(\text{nm}) \approx I_{600mV}(\text{nA}) = 6.9$

b. Positive Event Ratio = 23%; $d(\text{nm}) \approx I_{600mV}(\text{nA}) = 7.4$

c. Positive Event Ratio = 25%; $d(\text{nm}) \approx I_{600mV}(\text{nA}) = 8.4$

(3) c(MH nanolatch):c(MS2 carrier) = 10:1

a. Positive Event Ratio = 46%; $d(\text{nm}) \approx I_{600mV}(\text{nA}) = 9.0$

b. Positive Event Ratio = 44%; $d(\text{nm}) \approx I_{600mV}(\text{nA}) = 11.9$

c. Positive Event Ratio = 48%; $d(\text{nm}) \approx I_{600mV}(\text{nA}) = 10.5$

(4) c(MH nanolatch):c(MS2 carrier) = 15:1

a. Positive Event Ratio = 49%; $d(\text{nm}) \approx I_{600mV}(\text{nA}) = 7.5$

b. Positive Event Ratio = 41%; $d(\text{nm}) \approx I_{600mV}(\text{nA}) = 7.7$

c. Positive Event Ratio = 44%; $d(\text{nm}) \approx I_{600mV}(\text{nA}) = 9.3$

(5) c(MH nanolatch):c(MS2 carrier) = 20:1

a. Positive Event Ratio = 42%; $d(\text{nm}) \approx I_{600mV}(\text{nA}) = 9.6$

b. Positive Event Ratio = 41%; $d(\text{nm}) \approx I_{600mV}(\text{nA}) = 9.2$

c. Positive Event Ratio = 45%; $d(\text{nm}) \approx I_{600mV}(\text{nA}) = 10.4$

3.3 Dependence of positive event ratio on the lengths of pairing area and competitive area at the target site. (c(Nanolatch):c(MS2 carrier) = 10:1)

Example positive events

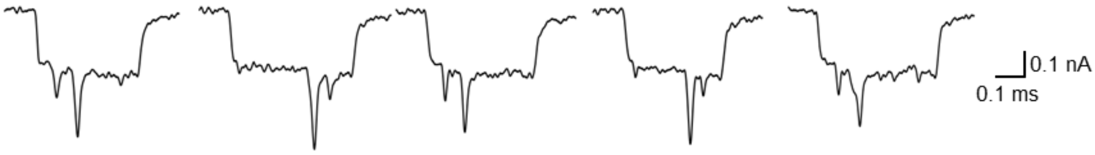

Example negative events

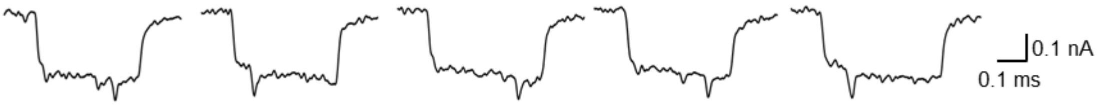

(1) Loop 1 (no mutation; 10 nt pairing area+8 nt competitive area)

a. Positive Event Ratio = 46%; d(nm)≈I_{600mV}(nA) = 9.0

b. Positive Event Ratio = 44%; d(nm)≈I_{600mV}(nA) = 11.9

c. Positive Event Ratio = 48%; d(nm)≈I_{600mV}(nA) = 10.5

(2) Loop 1(single-nucleotide substitution; 10 nt pairing area+8 nt competitive area)

a. Positive Event Ratio = 6%; d(nm)≈I_{600mV}(nA) = 11.2

b. Positive Event Ratio = 3%; d(nm)≈I_{600mV}(nA) = 10.4

c. Positive Event Ratio = 3%; d(nm)≈I_{600mV}(nA) = 11.2

(3) Loop 1_2 (no mutation; 8 nt pairing area+10 nt competitive area)

a. Positive Event Ratio = 22%; d(nm)≈I_{600mV}(nA) = 9.4

b. Positive Event Ratio = 24%; d(nm)≈I_{600mV}(nA) = 11.1

c. Positive Event Ratio = 23%; d(nm)≈I_{600mV}(nA) = 11.6

(4) Loop 1_2 (single-nucleotide substitution; 8 nt pairing area+10 nt competitive area)

a. Positive Event Ratio = 3%; d(nm)≈I_{600mV}(nA) = 9.2

b. Positive Event Ratio = 7%; d(nm)≈I_{600mV}(nA) = 8.6

c. Positive Event Ratio = 4%; d(nm)≈I_{600mV}(nA) = 9.6

(5) Loop 1_3 (no mutation; 10 nt pairing area+10 nt competitive area)

a. Positive Event Ratio = 22%; d(nm)≈I_{600mV}(nA) = 7.7

b. Positive Event Ratio = 18%; d(nm)≈I_{600mV}(nA) = 7.8

c. Positive Event Ratio = 23%; d(nm)≈I_{600mV}(nA) = 10.2

(6) Loop 1_3 (single-nucleotide substitution; 10 nt pairing area+10 nt competitive area)

a. Positive Event Ratio = 9%; d(nm)≈I_{600mV}(nA) = 7.3

b. Positive Event Ratio = 10%; d(nm)≈I_{600mV}(nA) = 12.5

c. Positive Event Ratio = 11%; d(nm)≈I_{600mV}(nA) = 7.3

(7) Loop 1_4 (no mutation; 10 nt pairing area+6 nt competitive area)

a. Positive Event Ratio = 37%; d(nm)≈I_{600mV}(nA) = 6.9

b. Positive Event Ratio = 37%; d(nm)≈I_{600mV}(nA) = 8.4

c. Positive Event Ratio = 43%; d(nm)≈I_{600mV}(nA) = 9.1

(8) Loop 1_4 (single-nucleotide substitution; 10 nt pairing area+6 nt competitive area)

a. Positive Event Ratio = 6%; d(nm)≈I_{600mV}(nA) = 9.0

b. Positive Event Ratio = 3%; d(nm)≈I_{600mV}(nA) = 8.8

c. Positive Event Ratio = 5%; d(nm)≈I_{600mV}(nA) = 8.4

(9) Loop 1_5 (no mutation; 12 nt pairing area+8 nt competitive area)

a. Positive Event Ratio = 28%; d(nm)≈I_{600mV}(nA) = 8.9

b. Positive Event Ratio = 35%; d(nm)≈I_{600mV}(nA) = 10.1

c. Positive Event Ratio = 32%; d(nm)≈I_{600mV}(nA) = 10.1

(10)Loop 1_5 (single-nucleotide substitution; 12 nt pairing area+8 nt competitive area)

a. Positive Event Ratio = 6%; d(nm)≈I_{600mV}(nA) = 7.8

b. Positive Event Ratio = 3%; d(nm)≈I_{600mV}(nA) = 11.3

c. Positive Event Ratio = 4%; d(nm)≈I_{600mV}(nA) = 7.2

(11)Loop 1_6 (no mutation; 8 nt pairing area+8 nt competitive area)

a. Positive Event Ratio = 22%; d(nm)≈I_{600mV}(nA) = 6.9

b. Positive Event Ratio = 15%; d(nm)≈I_{600mV}(nA) = 8.0

c. Positive Event Ratio = 19%; d(nm)≈I_{600mV}(nA) = 7.8

(12)Loop 1_6 (single-nucleotide substitution; 8 nt pairing area+8 nt competitive area)

a. Positive Event Ratio = 4%; d(nm)≈I_{600mV}(nA) = 8.1

b. Positive Event Ratio = 5%; d(nm)≈I_{600mV}(nA) = 12.1

c. Positive Event Ratio = 5%; d(nm)≈I_{600mV}(nA) = 10.3

3.4 Dependence of positive event ratio on the type and position of nucleotide mutations on the nanolatch. (c(Nanolatch):c(MS2 carrier) = 10:1)

Example positive events

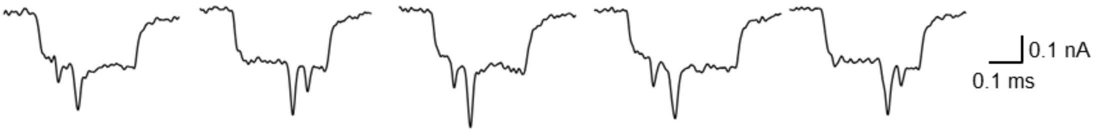

Example negative events

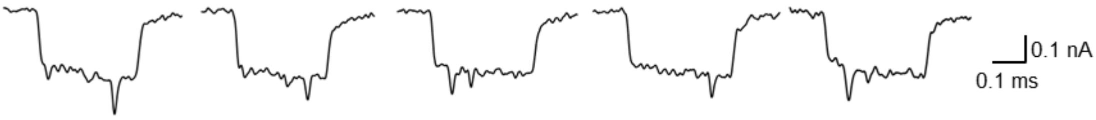

(1) MH; Original sequence

a. Positive Event Ratio = 46%; d(nm)≈I_{600mV}(nA) = 9.0

b. Positive Event Ratio = 44%; d(nm)≈I_{600mV}(nA) = 11.9

c. Positive Event Ratio = 48%; d(nm)≈I_{600mV}(nA) = 10.5

(2) MHm1; Single-nucleotide substitution (pairing area)

a. Positive Event Ratio = 4%; d(nm)≈I_{600mV}(nA) = 10.4

b. Positive Event Ratio = 6%; d(nm)≈I_{600mV}(nA) = 9.5

c. Positive Event Ratio = 8%; d(nm)≈I_{600mV}(nA) = 8.8

(3) MHm2; Single-nucleotide substitution (middle)

a. Positive Event Ratio = 6%; d(nm)≈I_{600mV}(nA) = 11.2

b. Positive Event Ratio = 3%; d(nm)≈I_{600mV}(nA) = 10.4

c. Positive Event Ratio = 3%; d(nm)≈I_{600mV}(nA) = 11.2

(4) MHm3; Single-nucleotide substitution (competitive area)

a. Positive Event Ratio = 17%; d(nm)≈I_{600mV}(nA) = 7.6

b. Positive Event Ratio = 19%; d(nm)≈I_{600mV}(nA) = 10.4

c. Positive Event Ratio = 15%; d(nm)≈I_{600mV}(nA) = 7.6

(5) MHm4; Two-nucleotide substitutions (middle + pairing area)

a. Positive Event Ratio = 2%; d(nm)≈I_{600mV}(nA) = 9.4

b. Positive Event Ratio = 0%; d(nm)≈I_{600mV}(nA) = 8.7

c. Positive Event Ratio = 2%; d(nm)≈I_{600mV}(nA) = 7.5

(6) MHm5; Single-nucleotide insertion (middle)

a. Positive Event Ratio = 7%; d(nm)≈I_{600mV}(nA) = 9.4

b. Positive Event Ratio = 8%; d(nm)≈I_{600mV}(nA) = 9.7

c. Positive Event Ratio = 12%; d(nm)≈I_{600mV}(nA) = 10.3

(7) MHm6; Single-nucleotide deletion (middle)

a. Positive Event Ratio = 7%; d(nm)≈I_{600mV}(nA) = 10.0

b. Positive Event Ratio = 5%; d(nm)≈I_{600mV}(nA) = 8.8

c. Positive Event Ratio = 6%; d(nm)≈I_{600mV}(nA) = 9.7

3.5 Dependence of positive event ratio on the number of 5mC modifications on the nanolatch. (c(Nanolatch):c(MS2 carrier) = 10:1)

Example positive events

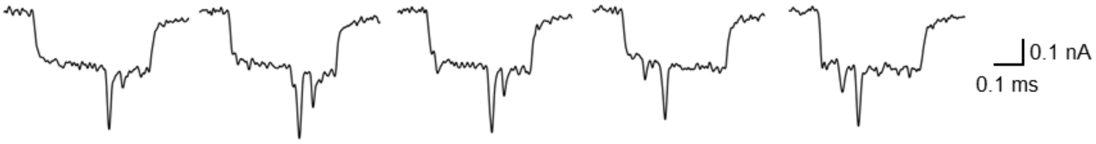

Example negative events

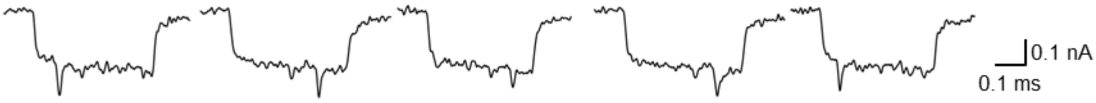

(1) MHC0; Original sequence

a. Positive Event Ratio = 48%; d(nm)≈I_{600mV}(nA) = 9.6

b. Positive Event Ratio = 44%; d(nm)≈I_{600mV}(nA) = 9.3

c. Positive Event Ratio = 51%; d(nm)≈I_{600mV}(nA) = 10.5

(2) MHC0_converted; Original sequence after bisulfite treatment

a. Positive Event Ratio = 1%; d(nm)≈I_{600mV}(nA) = 7.5

b. Positive Event Ratio = 3%; d(nm)≈I_{600mV}(nA) = 7.1

c. Positive Event Ratio = 5%; d(nm)≈I_{600mV}(nA) = 8.2

(3) MH5mC1; Single 5mC modification

a. Positive Event Ratio = 51%; d(nm)≈I_{600mV}(nA) = 7.6

b. Positive Event Ratio = 54%; d(nm)≈I_{600mV}(nA) = 7.7

c. Positive Event Ratio = 55%; d(nm)≈I_{600mV}(nA) = 7.9

(4) MH5mC1_converted; Single 5mC modification after bisulfite treatment

a. Positive Event Ratio = 11%; d(nm)≈I_{600mV}(nA) = 6.8

b. Positive Event Ratio = 7%; d(nm)≈I_{600mV}(nA) = 7.0

c. Positive Event Ratio = 10%; d(nm)≈I_{600mV}(nA) = 6.7

(5) MH5mC2; Two 5mC modifications

a. Positive Event Ratio = 57%; d(nm)≈I_{600mV}(nA) = 8.5

b. Positive Event Ratio = 56%; d(nm)≈I_{600mV}(nA) = 8.6

c. Positive Event Ratio = 61%; d(nm)≈I_{600mV}(nA) = 10.4

(6) MH5mC2_converted; Two 5mC modifications after bisulfite treatment

a. Positive Event Ratio = 14%; d(nm)≈I_{600mV}(nA) = 8.5

b. Positive Event Ratio = 15%; d(nm)≈I_{600mV}(nA) = 8.0

c. Positive Event Ratio = 14%; d(nm)≈I_{600mV}(nA) = 8.2

(7) MH5mC3; Three 5mC modifications

a. Positive Event Ratio = 61%; d(nm)≈I_{600mV}(nA) = 6.6

b. Positive Event Ratio = 64%; d(nm)≈I_{600mV}(nA) = 6.7

c. Positive Event Ratio = 66%; d(nm)≈I_{600mV}(nA) = 6.1

(8) MH5mC3_converted; Three 5mC modifications after bisulfite treatment

a. Positive Event Ratio = 22%; d(nm)≈I_{600mV}(nA) = 7.2

b. Positive Event Ratio = 19%; d(nm)≈I_{600mV}(nA) = 7.2

c. Positive Event Ratio = 18%; d(nm)≈I_{600mV}(nA) = 7.6

(9) MH5mC4; Four 5mC modifications

a. Positive Event Ratio = 67%; d(nm)≈I_{600mV}(nA) = 6.6

b. Positive Event Ratio = 70%; d(nm)≈I_{600mV}(nA) = 6.8

c. Positive Event Ratio = 69%; d(nm)≈I_{600mV}(nA) = 7.0

(10)MH5mC4_converted; Four 5mC modifications after bisulfite treatment

a. Positive Event Ratio = 53%; d(nm)≈I_{600mV}(nA) = 6.7

b. Positive Event Ratio = 48%; d(nm)≈I_{600mV}(nA) = 7.4

c. Positive Event Ratio = 55%; d(nm)≈I_{600mV}(nA) = 7.3

3.6 Dependence of positive event ratio on the number of inosines on the nanolatch.

(c(Nanolatch):c(MS2 carrier) = 10:1)

Example positive events

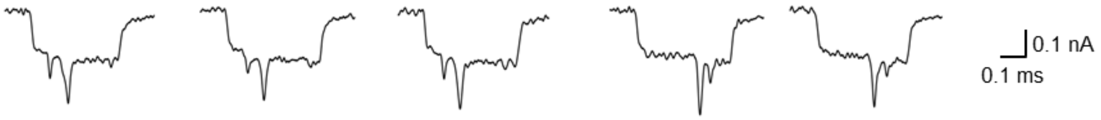

Example negative events

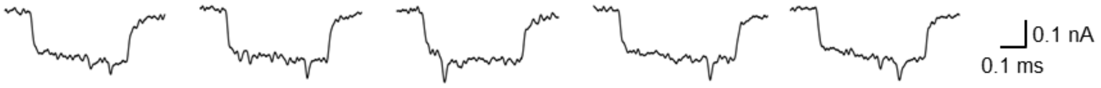

(1) MHI0; Original sequence

a. Positive Event Ratio = 47%; d(nm)$\approx I_{600mV}$(nA) = 8.8

b. Positive Event Ratio = 44%; d(nm)$\approx I_{600mV}$(nA) = 8.8

c. Positive Event Ratio = 50%; d(nm)$\approx I_{600mV}$(nA) = 8.8

(2) MHI1; Single inosine

a. Positive Event Ratio = 17%; d(nm)$\approx I_{600mV}$(nA) = 7.4

b. Positive Event Ratio = 20%; d(nm)$\approx I_{600mV}$(nA) = 8.0

c. Positive Event Ratio = 19%; d(nm)$\approx I_{600mV}$(nA) = 8.1

(3) MHI2; Two inosines

a. Positive Event Ratio = 10%; d(nm)$\approx I_{600mV}$(nA) = 7.5

b. Positive Event Ratio = 11%; d(nm)$\approx I_{600mV}$(nA) = 8.1

c. Positive Event Ratio = 7%; d(nm)$\approx I_{600mV}$(nA) = 8.6

(4) MHI3; Three inosines

a. Positive Event Ratio = 1%; d(nm)$\approx I_{600mV}$(nA) = 8.4

b. Positive Event Ratio = 1%; d(nm)$\approx I_{600mV}$(nA) = 8.0

c. Positive Event Ratio = 2%; d(nm)$\approx I_{600mV}$(nA) = 7.5

(5) MHI4; Four inosines

a. Positive Event Ratio = 2%; d(nm)$\approx I_{600mV}$(nA) = 6.4

b. Positive Event Ratio = 1%; d(nm)$\approx I_{600mV}$(nA) = 6.5

c. Positive Event Ratio = 1%; d(nm)$\approx I_{600mV}$(nA) = 8.6

3.7 Dependence of positive event ratio on the number of MeC modifications on the nanolatch. (c(Nanolatch):c(MS2 carrier) = 10:1)

Example positive events

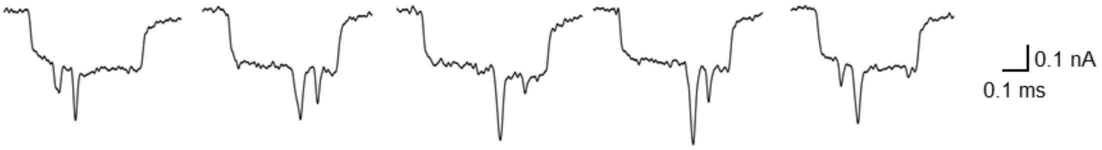

Example negative events

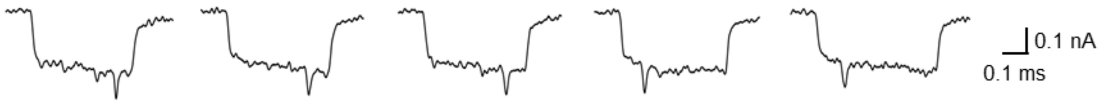

(1) MHC0; Original sequence

a. Positive Event Ratio = 48%; $d(\text{nm}) \approx I_{600mV}(\text{nA}) = 9.6$

b. Positive Event Ratio = 44%; $d(\text{nm}) \approx I_{600mV}(\text{nA}) = 9.3$

c. Positive Event Ratio = 51%; $d(\text{nm}) \approx I_{600mV}(\text{nA}) = 10.5$

(2) MHMeC1; Single MeC modification

a. Positive Event Ratio = 56%; $d(\text{nm}) \approx I_{600mV}(\text{nA}) = 8.4$

b. Positive Event Ratio = 61%; $d(\text{nm}) \approx I_{600mV}(\text{nA}) = 8.1$

c. Positive Event Ratio = 58%; $d(\text{nm}) \approx I_{600mV}(\text{nA}) = 8.1$

(3) MHMeC2; Two MeC modifications

a. Positive Event Ratio = 64%; $d(\text{nm}) \approx I_{600mV}(\text{nA}) = 7.5$

b. Positive Event Ratio = 68%; $d(\text{nm}) \approx I_{600mV}(\text{nA}) = 8.0$

c. Positive Event Ratio = 64%; $d(\text{nm}) \approx I_{600mV}(\text{nA}) = 7.7$

(4) MHMeC3; Three MeC modifications

a. Positive Event Ratio = 72%; $d(\text{nm}) \approx I_{600mV}(\text{nA}) = 8.3$

b. Positive Event Ratio = 74%; $d(\text{nm}) \approx I_{600mV}(\text{nA}) = 7.8$

c. Positive Event Ratio = 77%; $d(\text{nm}) \approx I_{600mV}(\text{nA}) = 7.5$

(5) MHMeC4; Four MeC modifications

a. Positive Event Ratio = 82%; $d(\text{nm}) \approx I_{600mV}(\text{nA}) = 7.5$

b. Positive Event Ratio = 79%; $d(\text{nm}) \approx I_{600mV}(\text{nA}) = 7.5$

c. Positive Event Ratio = 82%; $d(\text{nm}) \approx I_{600mV}(\text{nA}) = 9.3$

3.8 Detection of nucleotide variation between *E. coli* and *S.* Typhi 16S rRNA. (c(Nanolatch):c(16S RNA carrier) = 10:1)

Example positive events

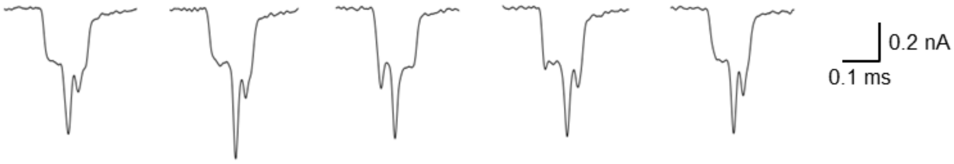

Example negative events

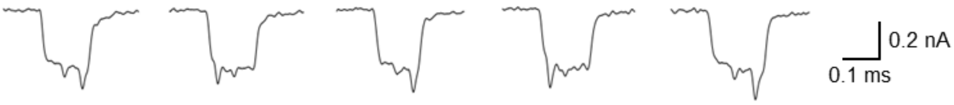

(1) *E. coli* total RNA + *E. coli* oligo pool

a. Positive Event Ratio = 49%; d(nm)≈I_{600mV}(nA) = 7.3

b. Positive Event Ratio = 55%; d(nm)≈I_{600mV}(nA) = 8.6

c. Positive Event Ratio = 55%; d(nm)≈I_{600mV}(nA) = 8.0

(2) *E. coli* total RNA + *S.* Typhi oligo pool

a. Positive Event Ratio = 6%; d(nm)≈I_{600mV}(nA) = 6.3

b. Positive Event Ratio = 9%; d(nm)≈I_{600mV}(nA) = 6.9

c. Positive Event Ratio = 3%; d(nm)≈I_{600mV}(nA) = 6.4

(3) *S.* Typhi total RNA + *S.* Typhi oligo pool

a. Positive Event Ratio = 52%; d(nm)≈I_{600mV}(nA) = 8.7

b. Positive Event Ratio = 51%; d(nm)≈I_{600mV}(nA) = 6.7

c. Positive Event Ratio = 53%; d(nm)≈I_{600mV}(nA) = 6.6

(4) *S.* Typhi total RNA + *E. coli* oligo pool

a. Positive Event Ratio = 6%; d(nm)≈I_{600mV}(nA) = 6.5

b. Positive Event Ratio = 5%; d(nm)≈I_{600mV}(nA) = 6.9

c. Positive Event Ratio = 4%; d(nm)≈I_{600mV}(nA) = 6.8

3.9 Quantification of *S.* Typhi and *S.* Enteritidis based on their nucleotide variations on 16s rRNA. (c(Nanolatch):c(16S RNA carrier) = 10:1)

Example positive events

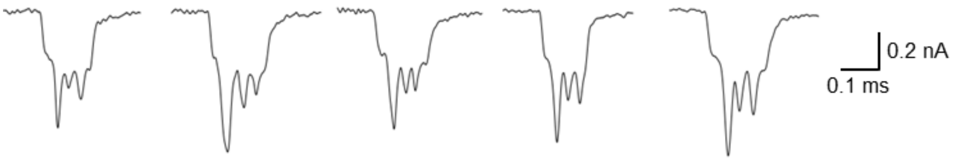

Example negative events

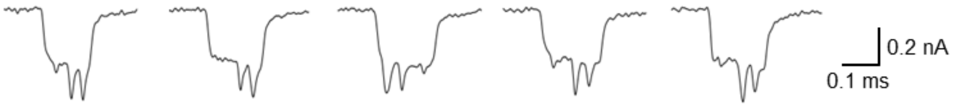

(1) *S.* Typhi total RNA + *S.* Typhi nanolatch

a. Positive Event Ratio = 50%; d(nm)$\approx I_{600mV}$(nA) = 7.5

b. Positive Event Ratio = 46%; d(nm)$\approx I_{600mV}$(nA) = 6.0

c. Positive Event Ratio = 54%; d(nm)$\approx I_{600mV}$(nA) = 8.9

(2) *S.* Typhi total RNA + *S.* Enteritidis nanolatch

a. Positive Event Ratio = 4%; d(nm)$\approx I_{600mV}$(nA) = 8.0

b. Positive Event Ratio = 4%; d(nm)$\approx I_{600mV}$(nA) = 7.0

c. Positive Event Ratio = 1%; d(nm)$\approx I_{600mV}$(nA) = 5.8

(3) (3 *S.* Typhi:1 *S.* Enteritidis) total RNA + *S.* Typhi nanolatch

a. Positive Event Ratio = 36%; d(nm)$\approx I_{600mV}$(nA) = 9.0

b. Positive Event Ratio = 40%; d(nm)$\approx I_{600mV}$(nA) = 7.2

c. Positive Event Ratio = 39%; d(nm)$\approx I_{600mV}$(nA) = 8.0

(4) (3 *S.* Typhi:1 *S.* Enteritidis) total RNA + *S.* Enteritidis nanolatch

a. Positive Event Ratio = 13%; d(nm)$\approx I_{600mV}$(nA) = 5.9

b. Positive Event Ratio = 14%; d(nm)$\approx I_{600mV}$(nA) = 6.6

c. Positive Event Ratio = 16%; d(nm)$\approx I_{600mV}$(nA) = 7.2

(5) (2 *S.* Typhi:2 *S.* Enteritidis) total RNA + *S.* Typhi nanolatch

a. Positive Event Ratio = 21%; d(nm)$\approx I_{600mV}$(nA) = 10.5

b. Positive Event Ratio = 28%; d(nm)$\approx I_{600mV}$(nA) = 8.6

c. Positive Event Ratio = 26%; d(nm)$\approx I_{600mV}$(nA) = 10.0

(6) (2 *S.* Typhi:2 *S.* Enteritidis) total RNA + *S.* Enteritidis nanolatch

a. Positive Event Ratio = 28%; d(nm)$\approx I_{600mV}$(nA) = 6.5

b. Positive Event Ratio = 23%; d(nm)$\approx I_{600mV}$(nA) = 6.2

c. Positive Event Ratio = 23%; d(nm)$\approx I_{600mV}$(nA) = 8.6

(7) (1 *S.* Typhi:3 *S.* Enteritidis) total RNA + *S.* Typhi nanolatch

a. Positive Event Ratio = 14%; d(nm)$\approx I_{600mV}$(nA) = 7.5

b. Positive Event Ratio = 15%; d(nm)$\approx I_{600mV}$(nA) = 7.1

c. Positive Event Ratio = 16%; d(nm)$\approx I_{600mV}$(nA) = 6.9

(8) (1 *S.* Typhi:3 *S.* Enteritidis) total RNA + *S.* Enteritidis nanolatch

a. Positive Event Ratio = 37%; d(nm)$\approx I_{600mV}$(nA) = 7.9

b. Positive Event Ratio = 36%; d(nm)$\approx I_{600mV}$(nA) = 6.5

c. Positive Event Ratio = 42%; d(nm)$\approx I_{600mV}$(nA) = 7.6

(9) *S.* Enteritidis total RNA + *S.* Typhi nanolatch

a. Positive Event Ratio = 1%; d(nm)$\approx I_{600mV}$(nA) = 6.8

b. Positive Event Ratio = 4%; d(nm)$\approx I_{600mV}$(nA) = 6.5

c. Positive Event Ratio = 4%; d(nm)$\approx I_{600mV}$(nA) = 7.4

(10)*S.* Enteritidis total RNA + *S.* Enteritidis nanolatch

a. Positive Event Ratio = 48%; d(nm)$\approx I_{600mV}$(nA) = 8.5

b. Positive Event Ratio = 53%; d(nm)$\approx I_{600mV}$(nA) = 8.1

c. Positive Event Ratio = 56%; d(nm)$\approx I_{600mV}$(nA) = 8.3

3.10 Detection of m^5C on *E. coli* 16S rRNA compared to *A. baumannii* 16S rRNA.

(c(Nanolatch):c(16S RNA carrier) = 10:1)

Example positive events

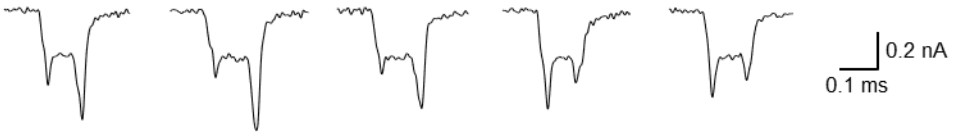

Example negative events

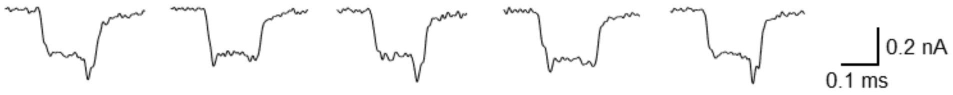

(1) ***E. coli*** **total RNA + EAH nanolatch**

a. Positive Event Ratio = 62%; d(nm)$\approx I_{600mV}$(nA) = 9.6

b. Positive Event Ratio = 67%; d(nm)$\approx I_{600mV}$(nA) = 8.8

c. Positive Event Ratio = 66%; d(nm)$\approx I_{600mV}$(nA) = 6.8

(2) ***A. baumannii*** **total RNA + EAH nanolatch**

a. Positive Event Ratio = 52%; d(nm)$\approx I_{600mV}$(nA) = 8.0

b. Positive Event Ratio = 57%; d(nm)$\approx I_{600mV}$(nA) = 8.0

c. Positive Event Ratio = 57%; d(nm)$\approx I_{600mV}$(nA) = 6.9

4. Nanopore Data Processing

At a commonly used sampling frequency of 1 MHz, 1,000,000 data points must be processed every second, including filtering, event detection, and classification. Supplementary Fig. 10 outlines the analysis pipeline, which processed a raw current trace input using custom-written programs in LabVIEW and Python to produce a list of classified translocation events and calculate the positive event ratio.

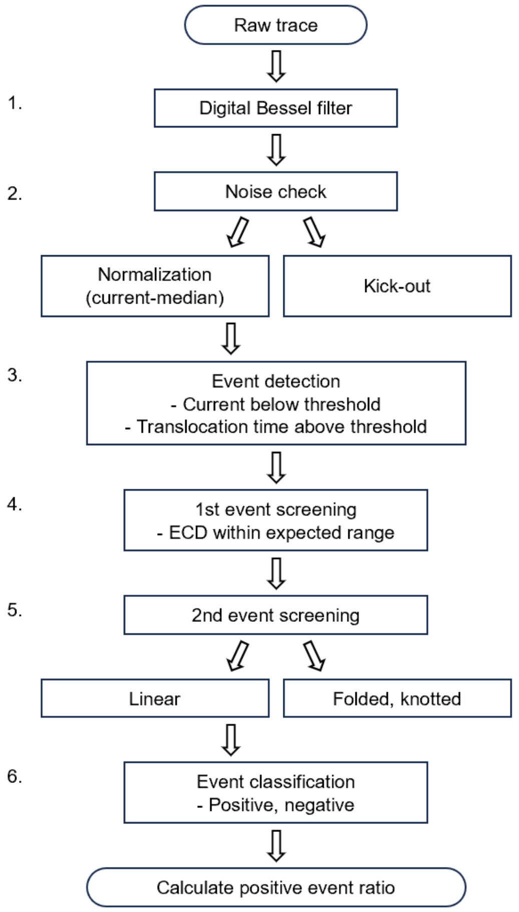

Supplementary Figure 10. Data processing pipeline for calculating positive event ratios. The raw current trace was first passed through a digital Bessel filter to remove high-frequency noise, followed by a noise check to make sure that the RMS noise remained below 7 pA, ensuring high data quality. After normalization by subtracting the median current level, events were selected based on two criteria: a current drop below a predefined threshold and a translocation time exceeding a set threshold. The first event screening was performed based on event charge deficit (ECD) ranges, which were determined by the length of the target carriers, leveraging prior nanopore measurements of similar molecule sizes and nanopore diameters to establish reference values. The second event screening filtered out folded and knotted events, retaining only linear translocation events. Following event selection, events were classified as positive or negative based on their specific nanostructure designs and current signal signatures. Positive event ratios were calculated as the number of positive events divided by total event count.

1. Digital Bessel filter

The raw ionic current data was recorded in three-second intervals. The first step in data processing was to remove high-frequency noise, which was achieved using a 100 kHz internal filter of the Axopatch 200B amplifier (Molecular Devices) and an eight-pole analog low-pass Bessel filter (900CT, Frequency Devices) with a cutoff frequency of 50 kHz.

2. Noise check

The second step involved assessing the noise level in the current trace to determine whether it was low enough for event detection. Typically, the standard deviation of the baseline current trace served as a noise metric. A LabVIEW-based noise assessment enabled the application of a simple threshold (50 pA), beyond which event detection was suspended, and corrective measures were taken, such as temporarily inverting the voltage to dislodge a molecule stuck in the pore (kick-out). If the noise level of the current baseline remained below 7 pA, events were normalized by subtracting the median current level before event detection.

3. Event detection

Once a current segment passed the noise check, it proceeded to the next step: event detection. The detection algorithm identified translocation events as deviations from the median current that fell below a predefined threshold, typically set at -100 pA. The minimum translocation time threshold varied depending on the sample being measured, as it was directly related to the target molecule's length. For the MS2 carriers analyzed in this study, full-length events generally lasted longer than 0.2 ms. For the shorter 16S rRNA carriers, the duration threshold was set to 0.1 ms to ensure that target translocation events were not filtered out.

4. 1st event screening

From the isolated events, the event charge deficit (ECD), which is defined as the difference between the total charge deficit and the expected baseline charge over the event duration, was plotted. Initial ECD screening ranges were set at 20-200 femtocoulomb (fc) for MS2 carriers and 10-100 fc for 16S rRNA carriers, encompassing all possible translocation events. A Kernel Smooth function was then applied to fit the data and determine the center and width of the ECD distribution (Supplementary Figs. 11-12). The ECD analysis provided a reliable method for sizing double-stranded carriers[14]. To ensure data quality, we retained only events within the main distribution of the fitted ECD curve, thereby excluding both carrier fragments (lower ECD range) and intermolecularly knotted carrier complexes (higher ECD range). Since the carriers used in this study varied in length, degradation susceptibility, and nanostructure distribution, it was challenging to establish a universal analysis criterion applicable to all carriers. Therefore, edge values were determined manually by examining events near valleys in bimodal distributions or quartiles in unimodal distributions.

5. 2nd event screening

The second event screening aimed to filter out the unidentifiable translocations and the type "21" events of identifiable carriers, a phenomenon where the carrier molecules exhibit a folded conformation at its entry point into the nanopore, as described by Bell, N.A.W. et al[15,16]. The fundamental principle of our approach to removing folded events is based on the distinct current trace models for linear and folded translocations. The linear event model is characterized by two current steps, corresponding to the molecule's entry and exit from the nanopore. In contrast, the folded model introduces an additional step at the beginning of an event, accounting for the molecule's folded region passing through the nanopore. To filter out folded events, we employed two methods. First, we set a filter requiring that the current within the first 15% of the translocation did not exceed 1.5 times the value of the double-stranded carrier level. As shown in Supplementary Fig. 13, any event exhibiting a type "21" fold conformation would

display an initial current change nearly twice the magnitude of the carrier level. Second, we calculated the percentage of data points in an event that fell outside the range of ± 25 pA relative to the carrier level. If more than 50% of the points exceeded this range, the event was classified as folded and excluded from further analysis. These approaches are supported by previous research[14-19]. Additionally, intramolecular knots formed on the carrier typically feature a current step approximately three times the magnitude of the first-level current drop generated by the carrier backbone. Based on this characteristic, we further filtered out knotted events by removing translocations where the maximum current drop exceeded 2.5 times the current change of the first plateau in the trace. For the carriers studied in this work, we typically observed linear event ratios of around 30% (Supplementary Figs. 11-12), although, in some high-quality measurements, linear event ratios exceeded 50% (Supplementary Fig. 12b, c). It is important to note that the observed variations in linear event ratios between measurements reflect the inherent variability of single-molecule experiments. Datasets were considered valid for further analysis when their linear event ratios fell within established experimental criteria. Moreover, this approach prevents the introduction of sequence-dependent bias when comparing different samples. We observed that minimal nucleotide mutations or modifications did not fundamentally alter the general translocation behavior of long carriers through nanopores, ensuring the consistent applicability of our linear event selection criteria across different samples.

6. Event classification

In the final step of the analysis pipeline, events were classified according to their spike signatures using Python scripts. Since the nanostructures were designed to be positioned at specific locations along the carrier, the secondary spike signals in the current trace were expected to appear within defined ranges during translocation events. For example, if the reference structure was positioned at 776-896 bp along the ~3.6 kb MS2 carrier, corresponding to approximately the 0.22nd quantile, we would expect the small spike associated with the reference structure to appear at around the 22% position in the translocation event as well. To identify these target spikes, we used the "scipy.signal.find_peaks" package in Python, which allowed us to detect spikes within specific regions of the translocation event and facilitated the determination of spike depth for each detected spike. By combining information on spike position and spike depth, we identified spikes corresponding to reference structures and then inferred the expected position of the latched loop signal, if present. To classify an event as positive or negative, we compared the spike depth detected at the loop position against a threshold of 0.55 relative to the first-level current drop. If the depth exceeded this threshold, the event was classified as positive. Otherwise, the event was classified as negative. The positive event ratio was then calculated as "the number of positive events / total event count × 100%".

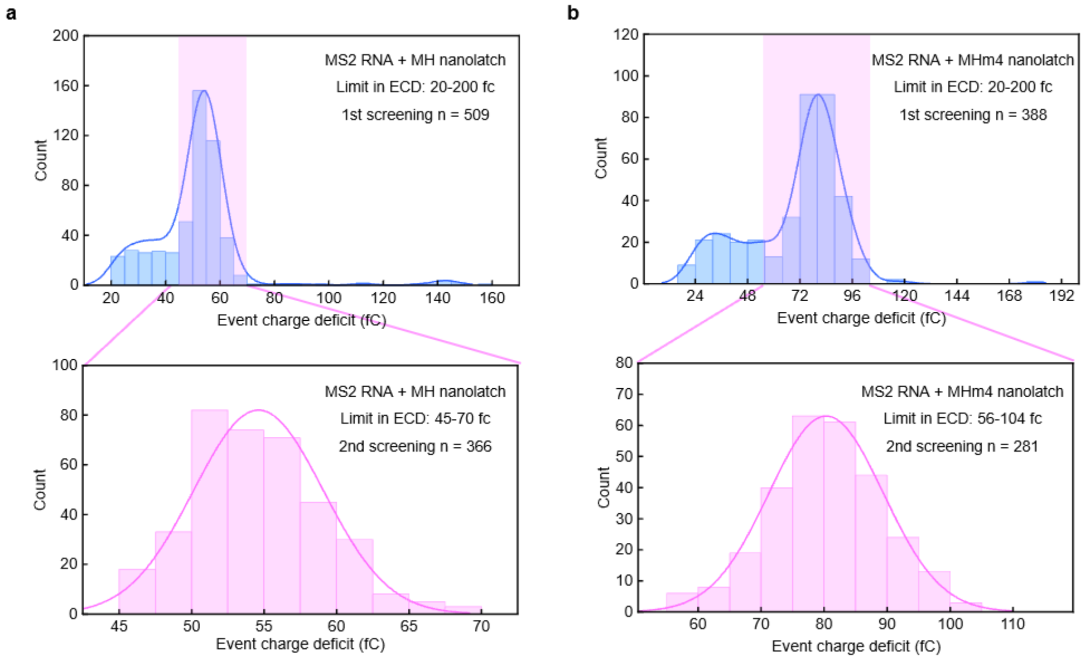

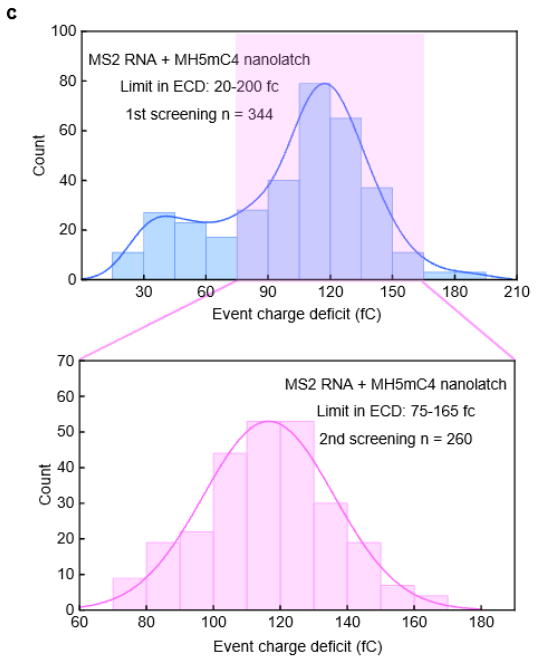

d

RNA target		MS2 RNA		
Nanolatch		MH	MHm4	MH5mC4
I_{600mV}		9.0 nA	9.4 nA	6.6 nA
Nucleotide variation		N/A	Two mutations	Four 5mC modifications
Positive event ratio		46%	2%	70%
1st screening event number		509	388	344
2nd screening event number	Linear	100	100	100
	Folded/ knotted	269	181	160
	Total	366	281	260
Linear event ratio = number of linear events / total number of events in 2nd screening		27%	36%	38%

Supplementary Figure 11. Representative data analysis of MS2 carrier nanopore measurements. Analysis of three distinct measurements illustrates our data processing approach: **a**, Using the fully complementary MH nanolatch yielded a positive event ratio of 46% with a linear event ratio of 27%. **b**, The MHm4 nanolatch containing two nucleotide mutations showed a positive event ratio of 2% with a linear event ratio of 36%. **c**, The MH5mC4 nanolatch incorporating four 5mC modifications demonstrated a positive event ratio of 70% with a linear event ratio of 38%. **d**, Detailed analysis workflow for these three measurements.

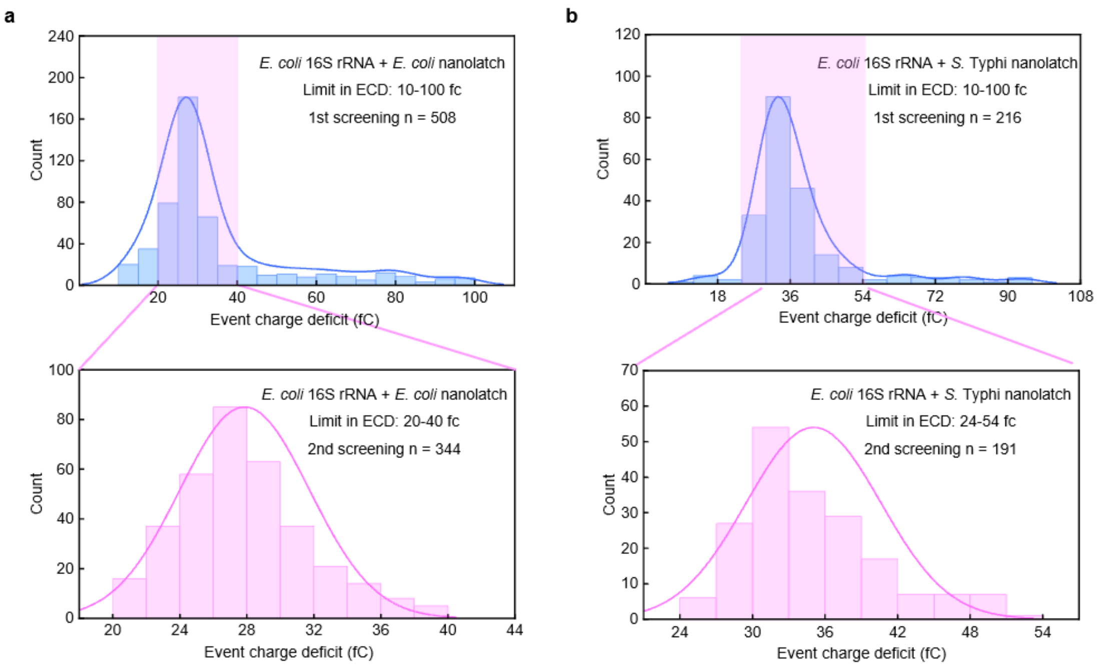

c

RNA target	Nanolatch	I_{600mV}	Nucleotide variation	Positive event ratio	1st screening event number	2nd screening event number			Linear event ratio
						Linear	Folded/knotted	Total	
E. coli 16S rRNA	*E. coli*	7.3 nA	N/A	49%	508	100	244	344	29%
	S. Typhi	6.3 nA	One mutation	6%	216	100	91	191	52%

Supplementary Figure 12. Representative data analysis of *E. coli* 16S rRNA carrier nanopore measurements. a, Analysis using the fully complementary *E. coli* nanolatch demonstrated a positive event ratio of 49% with a linear event ratio of 29%. **b,** The mismatched *S.* Typhi nanolatch yielded a positive event ratio of 6% with a linear event ratio of 52%. **c,** Detailed workflow illustrating the analysis process for both measurements.

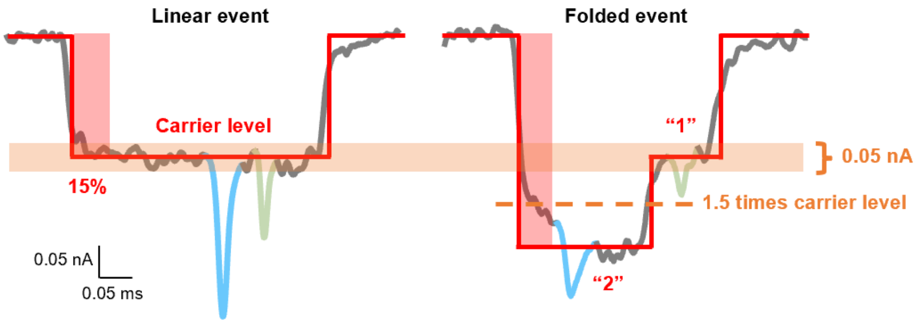

Supplementary Figure 13. Models and selection criteria for linear and folded events in nanopore translocation. Linear events are characterized by two current steps, corresponding to the molecule's entry and exit from the nanopore. Folded events present as a type "21" conformation, introducing an additional step at the event's start, representing the folded region passing through the pore. Selection criteria for linear events are twofold: the current must not exceed 1.5 times the double-stranded carrier level within the first 15% of the translocation, and over 50% of the data points must fall within ± 25 pA of the carrier level.

References

1. Bubienko, E., Cruz, P., Thomason, J.F., & Borer, P.N. Nearest-neighbor effects in the structure and function of nucleic acids. *Prog. Mol. Biol. Transl. Sci.* **30**, 41-90 (1983).
2. Sugimoto, N. et al. Thermodynamic parameters to predict stability of RNA/DNA hybrid duplexes. *Biochemistry* **34**, 11211-11216 (1995).
3. Crothers, D.M. & Zimm, B.H. Theory of the melting transition of synthetic polynucleotides: Evaluation of the stacking free energy. *J. Mol. Biol.* **9**, 1-9 (1964).
4. DeVoe, H. & Tinoco, I. The stability of helical polynucleotides: Base contributions. *J. Mol. Biol.* **4**, 500-517 (1962).
5. Wright, D.J., Rice, J.L., Yanker, D.M. & Znosko, B.M. Nearest neighbor parameters for inosine·uridine pairs in RNA duplexes. *Biochemistry* **46**, 4625-4634 (2007).
6. SantaLucia, J. A unified view of polymer, dumbbell, and oligonucleotide DNA nearest-neighbor thermodynamics. *Proc. Natl. Acad. Sci.* **95**, 1460-1465 (1998).
7. Tsuruta, M., Sugitani, Y., Sugimoto, N. & Miyoshi, D. Combined effects of methylated cytosine and molecular crowding on the thermodynamic stability of DNA duplexes. *Int. J. Mol. Sci.* **22**, 947 (2021).
8. Hopfinger, M.C., Kirkpatrick, C.C. & Znosko, B.M. Predictions and analyses of RNA nearest neighbor parameters for modified nucleotides. *Nucleic Acids Res.* **48**, 8901-8913 (2020).
9. Watkins, N.E. Nearest-neighbor thermodynamics of deoxyinosine pairs in DNA duplexes. *Nucleic Acids Res.* **33**, 6258-6267 (2005).
10. Xiang, T., Feng, H., Xing, X. & Zhang, C. Thermodynamic parameters contributions of single internal mismatches in RNA/DNA hybrid duplexes. *bioRxiv* doi:10.1101/2022.11.25.517909 (2022).
11. Tan, Z.J. & Chen, S.J. Nucleic acid helix stability: Effects of salt concentration, cation valence and size, and chain length. *Biophys. J.* **90**, 1175-1190 (2006).
12. Kierzek, R., Burkard, M.E. & Turner, D.H. Thermodynamics of single mismatches in RNA duplexes. *Biochemistry* **38**, 14214-14223 (1999).
13. NUPACK Web Application. https://docs.nupack.org/
14. Bell, N.A.W., Muthukumar, M. & Keyser, U.F. Translocation frequency of double-stranded DNA through a solid-state nanopore. *Phys. Rev. E* **93**, 022401 (2016).
15. Bell, N.A.W. & Keyser, U.F. Digitally encoded DNA nanostructures for multiplexed, single-molecule protein sensing with nanopores. *Nat. Nanotechnol.* **11**, 645 (2016).
16. Bell, N.A.W. & Keyser, U.F. Specific protein detection using designed DNA carriers and nanopores. *J. Am. Chem. Soc.* **137**, 2035-2041(2015).
17. Ermann, N. et al. (2018). Promoting single-file DNA translocations through nanopores using electro-osmotic flow. *J. Chem. Phys.* **149**, 16 (2018).
18. Ermann, N. Nanopore-Based Readout of Encoded DNA Nanostructures. *Doctoral dissertation, University of Cambridge* (2020).
19. Bošković, F. et al. Nanopore Translocation Reveals Electrophoretic Force on Noncanonical RNA: DNA Double Helix. *ACS Nano* (2024).
