# Supplementary figures and images for: Nanopore detection of single-nucleotide RNA mutations and modifications with programmable nanolatches
Source: Nat Nanotechnol. 2025 Jun 27;20(10):1473–81. doi: 10.1038/s41565-025-01965-6 (PMC12534182; doi:10.1038/s41565-025-01965-6)

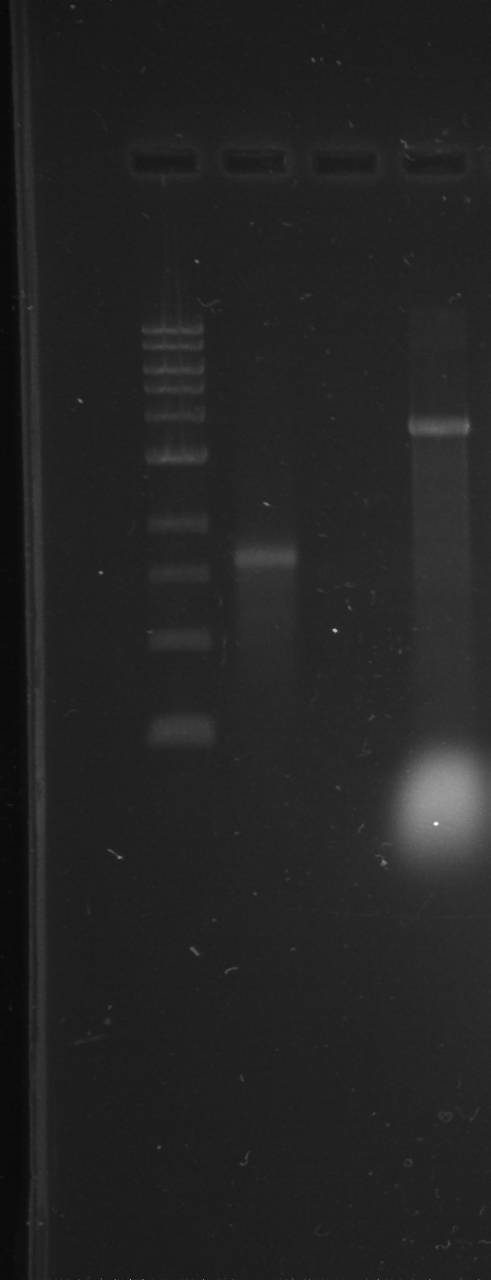

Supplement: Supplementary file 3 — Gel image for Supplementary Fig. 2. [file 41565_2025_1965_MOESM3_ESM.tiff]

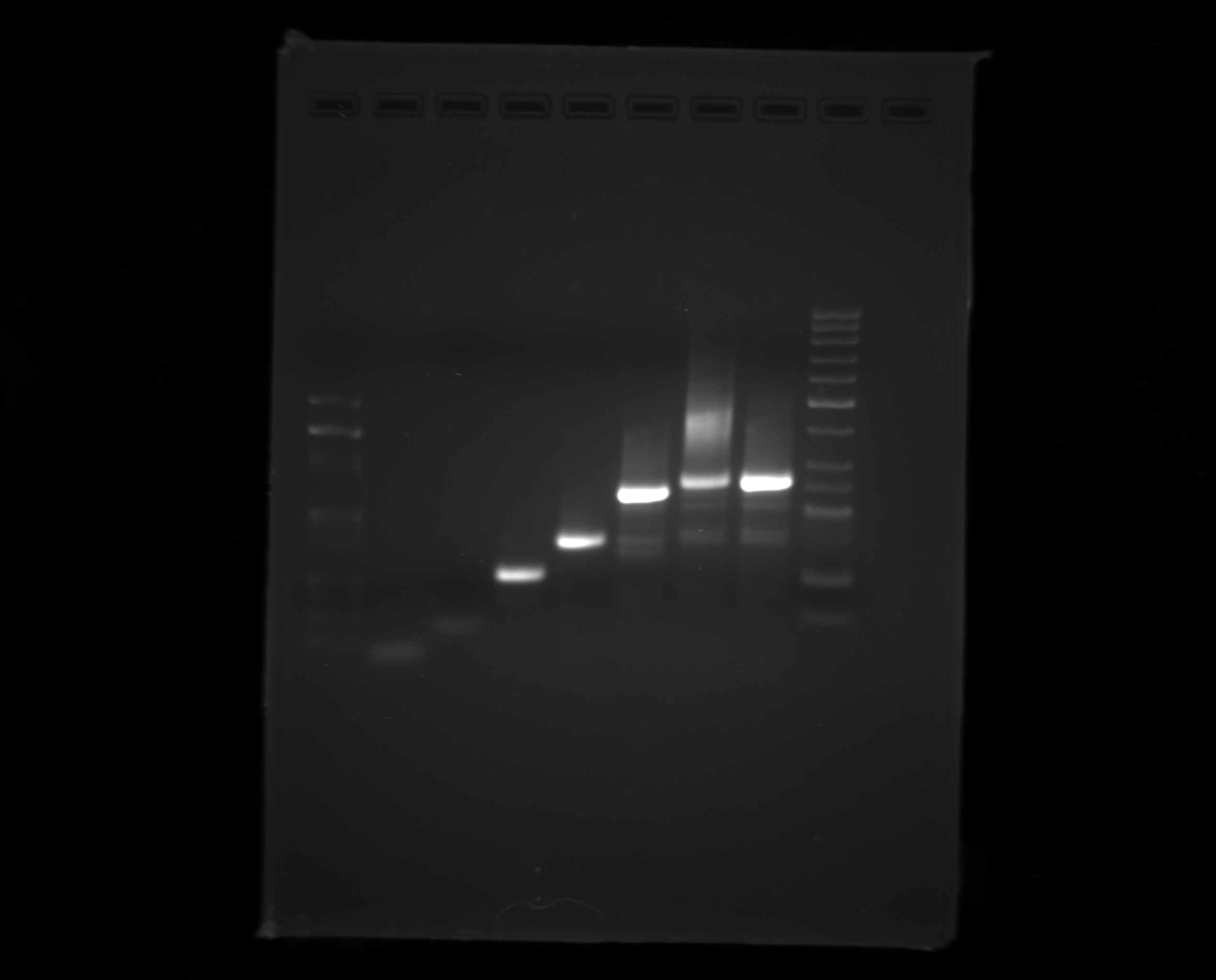

Supplement: Supplementary file 10 — Gel image. [file 41565_2025_1965_MOESM10_ESM.tif]
